# Supplementary material for: Stressors, manifestations and course of COVID-19 related distress among public sector nurses and midwives during the COVID-19 pandemic first year in Tasmania, Australia
Source: PLoS One. 2022 Aug 9;17(8):e0271824. doi: 10.1371/journal.pone.0271824 (PMC9362919; doi:10.1371/journal.pone.0271824)
Supplement: S1 File — (PDF) [file pone.0271824.s001.pdf]

[illegible]

## Demographics

### 1. Employee classification

- ◯ RN
- ◯ MIDWIFE
- ◯ EN
- ◯ AIN
- ◯ Other (please specify)

[ ]

### 2. What department/area do you predominantly work in?

- ☐ COLD AREA (COVID-19 NEGATIVE patients/clients)
- ☐ HOT AREA (COVID-19 POSITIVE patients/clients)

### 3. Which REGION of Tasmania do you work?

- ◯ North
- ◯ North-West
- ◯ South
- ◯ Statewide

### 4. Gender

- ◯ Female
- ◯ Male
- ◯ Other (please specify)

[ ]

### 5. Educational level

- ◯ Undergraduate level
- ◯ University degree - Bachelor
- ◯ Higher degree - Grad Cert or higher
- ◯ Other (please specify)
- ◯ TAFE Diploma
- ◯ Hospital training certificate

[ ]

6. What is your AGE GROUP?

- 18 - 25
- 26 - 30
- 31 - 40
- 41 - 50
- 51 - 60
- 61 - 70
- 71 - 80
- 81+

7. Do you smoke?

- Yes
- No
- Prefer not to answer

[illegible]

*If this survey raises any difficult issues for you contact EAP 1300687327*

**These questions are about your HOME and FAMILY.**

8. To what degree has COVID-19 contributed to your CURRENT home and family stress

| Not at all | Some | Moderately | Extremely | Don't know | Prefer not to answer |
|---|---|---|---|---|---|
| ◯ | ◯ | ◯ | ◯ | ◯ | ◯ |

Comments

9. To what degree do you consider COVID-19 will contribute to your FUTURE home and family stress

| Not at all | Some | Moderately | Extremely | Don't know | Prefer not to answer |
|---|---|---|---|---|---|
| ◯ | ◯ | ◯ | ◯ | ◯ | ◯ |

Comment

10. To what degree has COVID-19 contributed to your CURRENT financial stress

| Not at all | Some | Moderately | Extremely | Don't know | Prefer not to answer |
|---|---|---|---|---|---|
| ◯ | ◯ | ◯ | ◯ | ◯ | ◯ |

Comment

11. To what degree do you consider COVID-19 will contribute to your FUTURE financial stress

| Not at all | Some | Moderately | Extremely | Don't know | Prefer not to answer |
|---|---|---|---|---|---|
| ◯ | ◯ | ◯ | ◯ | ◯ | ◯ |

Comment

[illegible]

*If this survey raises any difficult issues for you contact EAP 1300687327*

[illegible]

**These questions are about COVID-19 exposure.**

12. Have you been EXPOSED to COVID-19?

- Yes
- No
- Unsure
- Prefer not to answer

13. Have YOU been diagnosed with COVID-19 ?

- Yes
- No
- Unsure
- Prefer not to answer

14. Do you MANAGE PATIENTS / CLIENTS diagnosed with COVID-19?

- Yes
- No
- Unsure
- Prefer not to answer

15. Has ANYONE in your FAMILY been diagnosed with COVID-19?

- Yes
- No
- Unsure
- Prefer not to say

16. Have any of your FRIENDS been diagnosed with COVID-19?

- Yes
- No
- Unsure
- Prefer not to answer

17. Have any of your NEIGHBOURS been diagnosed with COVID-19?

- Yes
- No
- Unsure
- Prefer not to answer

18. How confident are you in the validity of the COVID-19 test?

| Not at all | Neutral | Confident | Extremely confident | Prefer not to answer |
|---|---|---|---|---|
| ○ | ○ | ○ | ○ | ○ |

Other

If this survey raises any difficult issues for you contact EAP 1300687327

**These questions are about WORKPLACE SUPPORT.**

19. Are you concerned about adequate access to PPE?

| Not concerned | Moderately concerned | Very concerned | Don't know | Prefer not to answer |
|---|---|---|---|---|
| ○ | ○ | ○ | ○ | ○ |

20. Do you feel you have adequate access to rapid COVID-19 testing if required?

| All of the time | Most of the time | Some of the time | None of the time | Don't know | Prefer not to answer |
|---|---|---|---|---|---|
| ○ | ○ | ○ | ○ | ○ | ○ |

21. Do you feel you have adequate access to up to date information, clear communications and guidelines?

| All the time | Most of the time | Some of the time | Never | Don't know | Prefer not to answer |
|---|---|---|---|---|---|
| ○ | ○ | ○ | ○ | ○ | ○ |

22. Do you feel you have received EXCESS information and communications relating to COVID-19?

| All the time | Most of the time | Some of the time | Never | Don't know | Prefer not to answer |
|---|---|---|---|---|---|
| ○ | ○ | ○ | ○ | ○ | ○ |

23. Do you feel you have been adequately prepared to provide competent care in the event you will be deployed to a new area?

| Definitely | Moderately | Slightly | Not at all | Not applicable | Prefer not to answer |
|---|---|---|---|---|---|
| ○ | ○ | ○ | ○ | ○ | ○ |

24. Do you feel adequately supported by your workplace team (colleagues and line manager)?

| All of the time | Most of the time | Some of the time | Never | Don't know | Prefer not to answer |
|---|---|---|---|---|---|
| ○ | ○ | ○ | ○ | ○ | ○ |

25. Do you feel your workplace has supported you in the provision of food and accommodation as your workplace demands increase?

| All of the time | Most of the time | Some of the time | Never | Not applicable | Don't know | Prefer not to answer |
|---|---|---|---|---|---|---|
| ○ | ○ | ○ | ○ | ○ | ○ | ○ |

26. Do you feel your workplace has supported you in the provision of transportation as your workplace demands increase?

| All of the time | Most of the time | Some of the time | Never | Not applicable | Don't know | Prefer not to answer |
|---|---|---|---|---|---|---|
| ◯ | ◯ | ◯ | ◯ | ◯ | ◯ | ◯ |

## [illegible]

*If this survey raises any difficult issues for you contact EAP 1300687327*

**During the past TWO WEEKS how would you rate your sleep?**

27. Difficulty FALLING asleep

| None | Mild | Moderate | Severe | Very severe |
|---|---|---|---|---|
| ◯ | ◯ | ◯ | ◯ | ◯ |

28. Difficulty STAYING asleep

| None | Mild | Moderate | Severe | Very severe |
|---|---|---|---|---|
| ◯ | ◯ | ◯ | ◯ | ◯ |

29. Problems WAKING UP too early

| None | Mild | Moderate | Severe | Very severe |
|---|---|---|---|---|
| ◯ | ◯ | ◯ | ◯ | ◯ |

30. How SATISFIED/DISSATISFIED are you with your CURRENT sleep pattern?

| Very Satisfied | Satisfied | Moderately Satisfied | Dissatisfied | Very Dissatisfied |
|---|---|---|---|---|
| ◯ | ◯ | ◯ | ◯ | ◯ |

31. How NOTICEABLE to others do you think your sleep problem is in terms of impairing the quality of your life?

| Not at all Noticeable | A Little | Somewhat | Much | Very Much Noticeable |
|---|---|---|---|---|
| ◯ | ◯ | ◯ | ◯ | ◯ |

32. How WORRIED/DISTRESSED are you about your current sleep problem?

| Not at all Worried | A Little | Somewhat | Much | Very Much Worried |
|---|---|---|---|---|
| ◯ | ◯ | ◯ | ◯ | ◯ |

33. To what extent do you consider your sleep problem to INTERFERE with your daily functioning (e.g. daytime fatigue, mood, ability to function at work/daily chores, concentration, memory, mood, etc.) CURRENTLY?

| Not at all Interfering | A Little | Somewhat | Much | Very Much Interfering |
|---|---|---|---|---|
| ◯ | ◯ | ◯ | ◯ | ◯ |

*If this survey raises any difficult issues for you contact EAP 1300687327*

**These questions are about anxiety.**

**How often have you been bothered by any of the following problems over the last TWO WEEKS?**

34. Feeling nervous, anxious or on edge?

| Not at all | Several days | More than half the days | Nearly every day |
|---|---|---|---|
| ○ | ○ | ○ | ○ |

35. Not being able to stop or control worrying?

| Not at all | Several days | More than half the days | Nearly every day |
|---|---|---|---|
| ○ | ○ | ○ | ○ |

36. Worrying too much about different things?

| Not at all | Several days | More than half the days | Nearly every day |
|---|---|---|---|
| ○ | ○ | ○ | ○ |

37. Trouble relaxing?

| Not at all | Several days | More than half the days | Nearly every day |
|---|---|---|---|
| ○ | ○ | ○ | ○ |

38. Being so restless that it is hard to sit still?

| Not at all | Several days | More than half the days | Nearly every day |
|---|---|---|---|
| ○ | ○ | ○ | ○ |

39. Being easily annoyed or irritable?

| Not at all | Several days | More than half the days | Nearly every day |
|---|---|---|---|
| ○ | ○ | ○ | ○ |

40. Feeling afraid as if something awful might happen?

| Not at all | Several days | More than half the days | Nearly every day |
|---|---|---|---|
| ○ | ○ | ○ | ○ |

41. What factors (if any) cause you anxiety and stress related to COVID-19?

MINDHEALTH

*If this survey raises any difficult issues for you contact EAP 1300687327*

**Below is a list of questions related to difficulties people sometimes have after stressful events.**

**Please read each item and then indicate how distressing each difficulty has been for you DURING THE PAST SEVEN DAYS with respect to COVID-19.**

**How much were you distressed or bothered by these difficulties?**

42. Any reminder brought back feelings about it

| not at all | a little bit | moderately | quite a bit | extremely |
|---|---|---|---|---|
| ○ | ○ | ○ | ○ | ○ |

43. I had trouble staying asleep

| not at all | a little bit | moderately | quite a bit | extremely |
|---|---|---|---|---|
| ○ | ○ | ○ | ○ | ○ |

44. Other things kept making me think about it

| not at all | a little bit | moderately | quite a bit | extremely |
|---|---|---|---|---|
| ○ | ○ | ○ | ○ | ○ |

45. I felt irritable and angry

| not at all | a little bit | moderately | quite a bit | extremely |
|---|---|---|---|---|
| ○ | ○ | ○ | ○ | ○ |

46. I avoided letting myself get upset when I thought about it or was reminded of it

| not at all | a little bit | moderately | quite a bit | extremely |
|---|---|---|---|---|
| ○ | ○ | ○ | ○ | ○ |

47. I thought about it when I didn't mean to

| not at all | a little bit | moderately | quite a bit | extremely |
|---|---|---|---|---|
| ○ | ○ | ○ | ○ | ○ |

48. I felt as if it hadn't happened or wasn't real

| not at all | a little bit | moderately | quite a bit | extremely |
|---|---|---|---|---|
| ○ | ○ | ○ | ○ | ○ |

49. I stayed away from reminders about it

| not at all | a little bit | moderately | quite a bit | extremely |
|---|---|---|---|---|

50. Pictures about it popped into my mind

| not at all | a little bit | moderately | quite a bit | extremely |
|---|---|---|---|---|

51. I was jumpy and easily startled

| not at all | a little bit | moderately | quite a bit | extremely |
|---|---|---|---|---|

52. I tried not to think about it

| not at all | a little bit | moderately | quite a bit | extremely |
|---|---|---|---|---|

53. I was aware that I still had a lot of feelings about it, but I didn't deal with them.

| not at all | a little bit | moderately | quite a bit | extremely |
|---|---|---|---|---|

54. My feelings about it were kind of numb

| not at all | a little bit | moderately | quite a bit | extremely |
|---|---|---|---|---|

55. I found myself acting or feeling as though I was back at that time

| not at all | a little bit | moderately | quite a bit | extremely |
|---|---|---|---|---|

56. I had trouble falling asleep

| not at all | a little bit | moderately | quite a bit | extremely |
|---|---|---|---|---|

57. I had waves of strong feelings about it

| not at all | a little bit | moderately | quite a bit | extremely |
|---|---|---|---|---|

58. I tried to remove it from my memory

| not at all | a little bit | moderately | quite a bit | extremely |
|---|---|---|---|---|

59. I had trouble concentrating

| not at all | a little bit | moderately | quite a bit | extremely |
|---|---|---|---|---|
| ○ | ○ | ○ | ○ | ○ |

60. Reminders of it caused me to have physical reactions such as sweating, trouble breathing, nausea or a pounding heart.

| not at all | a little bit | moderately | quite a bit | extremely |
|---|---|---|---|---|
| ○ | ○ | ○ | ○ | ○ |

61. I had dreams about it

| not at all | a little bit | moderately | quite a bit | extremely |
|---|---|---|---|---|
| ○ | ○ | ○ | ○ | ○ |

62. I felt watchful or on-guard

| not at all | a little bit | moderately | quite a bit | extremely |
|---|---|---|---|---|
| ○ | ○ | ○ | ○ | ○ |

63. I tried not to talk about it

| not at all | a little bit | moderately | quite a bit | extremely |
|---|---|---|---|---|
| ○ | ○ | ○ | ○ | ○ |
