# Supplementary figures and images for: Stressors, manifestations and course of COVID-19 related distress among public sector nurses and midwives during the COVID-19 pandemic first year in Tasmania, Australia
Source: PLoS One. 2022 Aug 9;17(8):e0271824. doi: 10.1371/journal.pone.0271824 (PMC9362919; doi:10.1371/journal.pone.0271824)

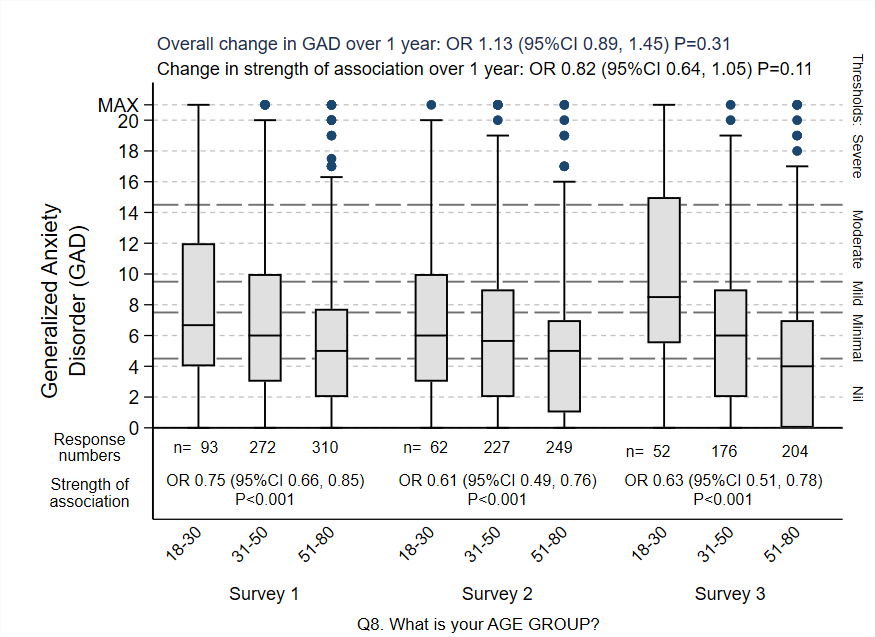

Supplement: S2 File — (ZIP) [file pone.0271824.s002.zip › Age2_GAD7_V3.3_040522.tif]

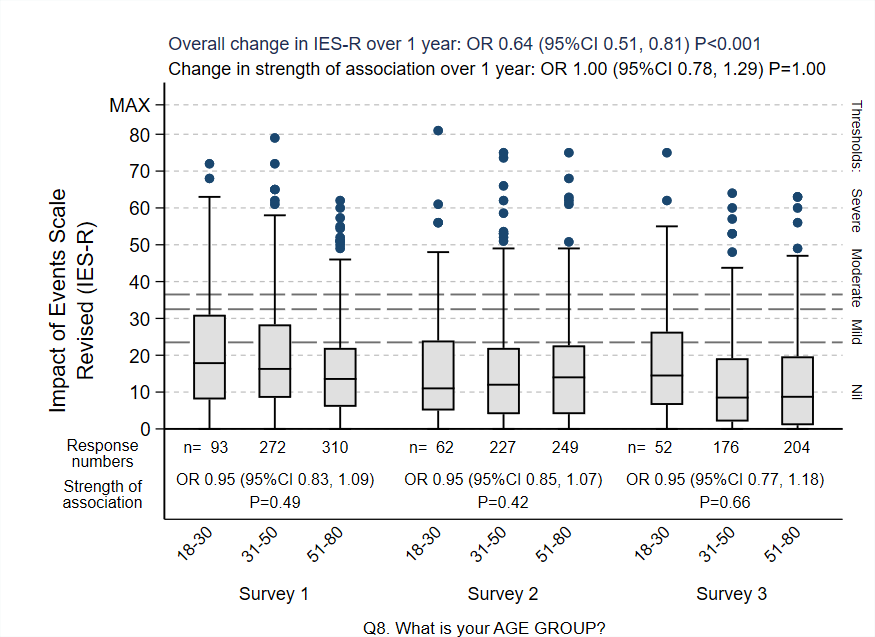

Supplement: S2 File — (ZIP) [file pone.0271824.s002.zip › Age2_IESR22_V3.3_040522.tif]

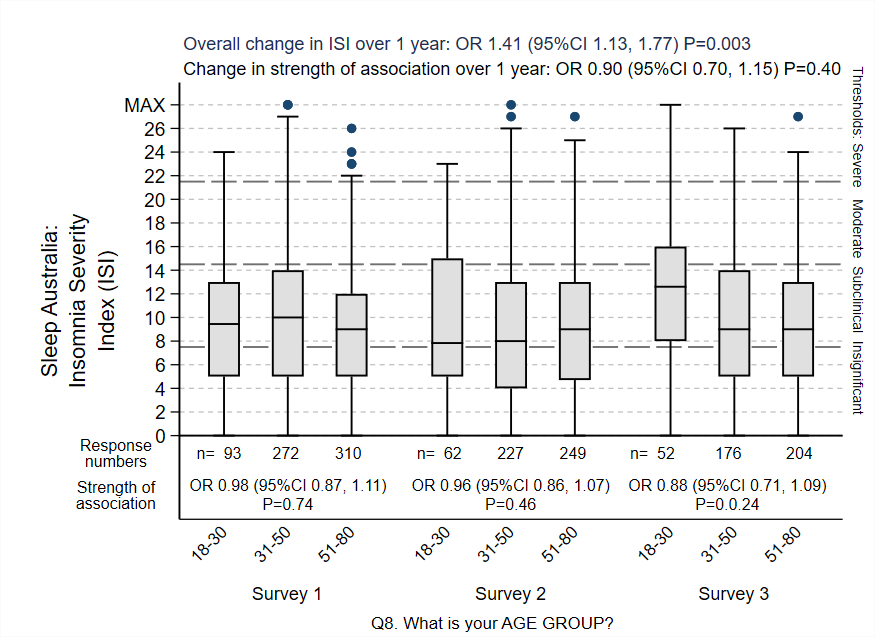

Supplement: S2 File — (ZIP) [file pone.0271824.s002.zip › Age2_ISI7_V3.3_040522.tif]

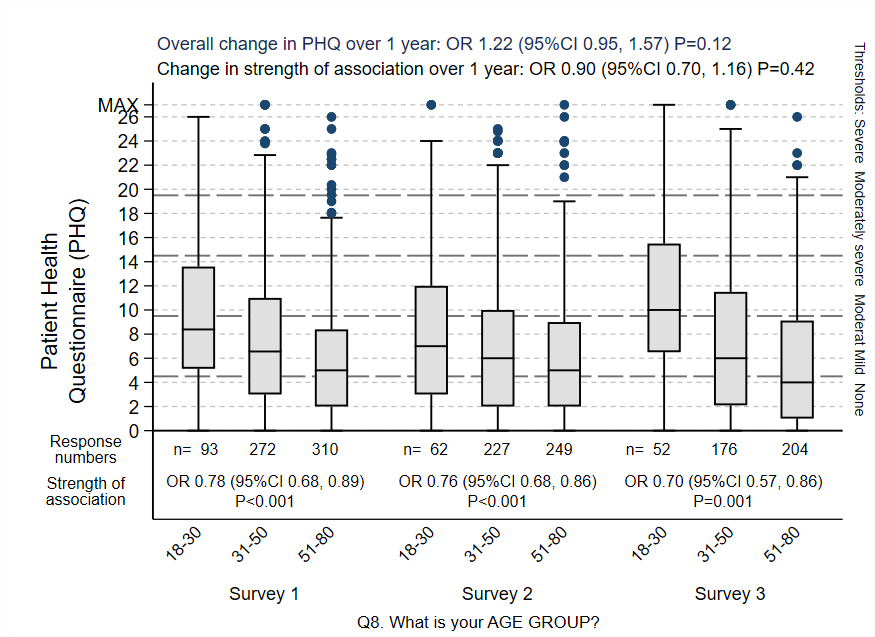

Supplement: S2 File — (ZIP) [file pone.0271824.s002.zip › Age2_PHQ9_V3.3_040522.tif]

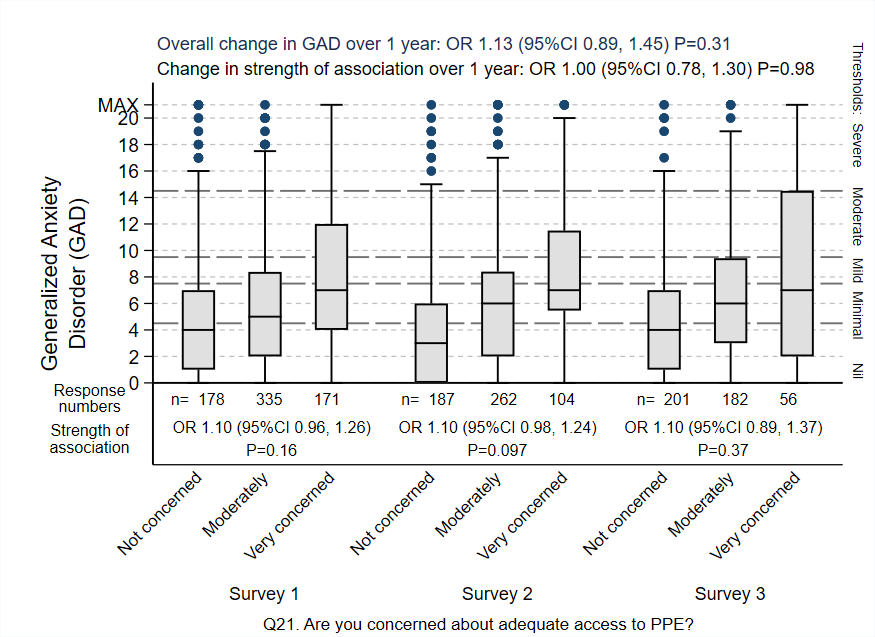

Supplement: S2 File — (ZIP) [file pone.0271824.s002.zip › ConPPE_GAD7_V3.3_040522.tif]

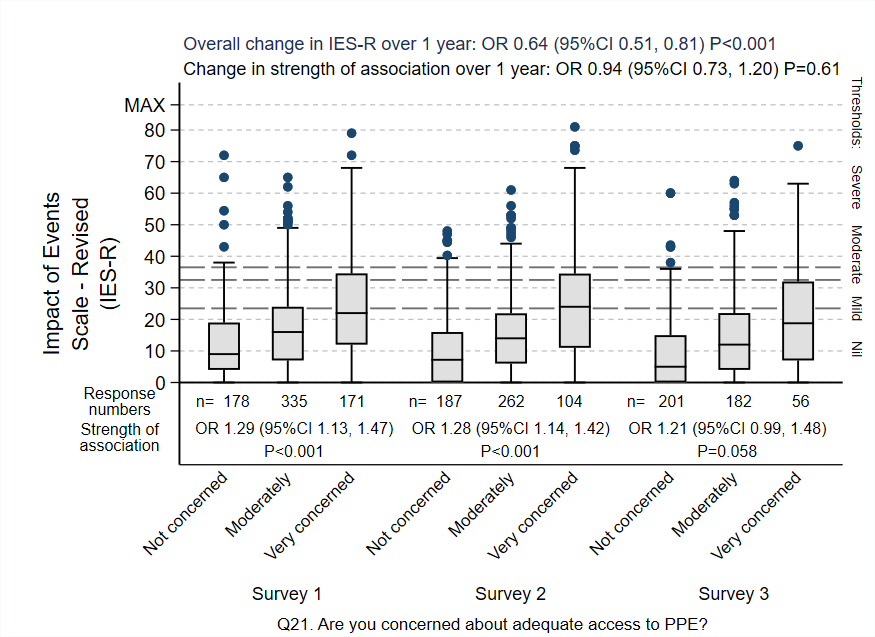

Supplement: S2 File — (ZIP) [file pone.0271824.s002.zip › ConPPE_IESR22_V3.3_040522.tif]

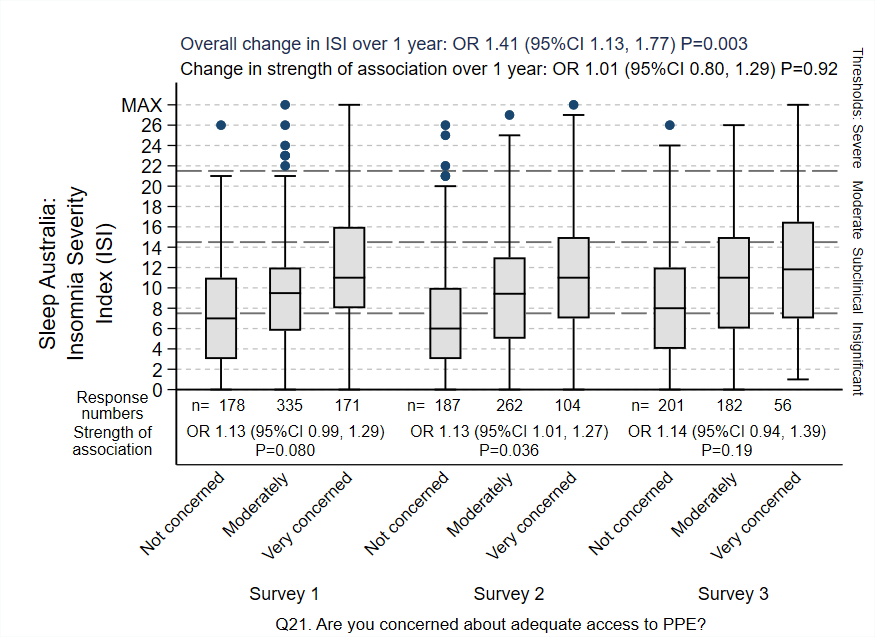

Supplement: S2 File — (ZIP) [file pone.0271824.s002.zip › ConPPE_ISI7_V3.3_040522.tif]

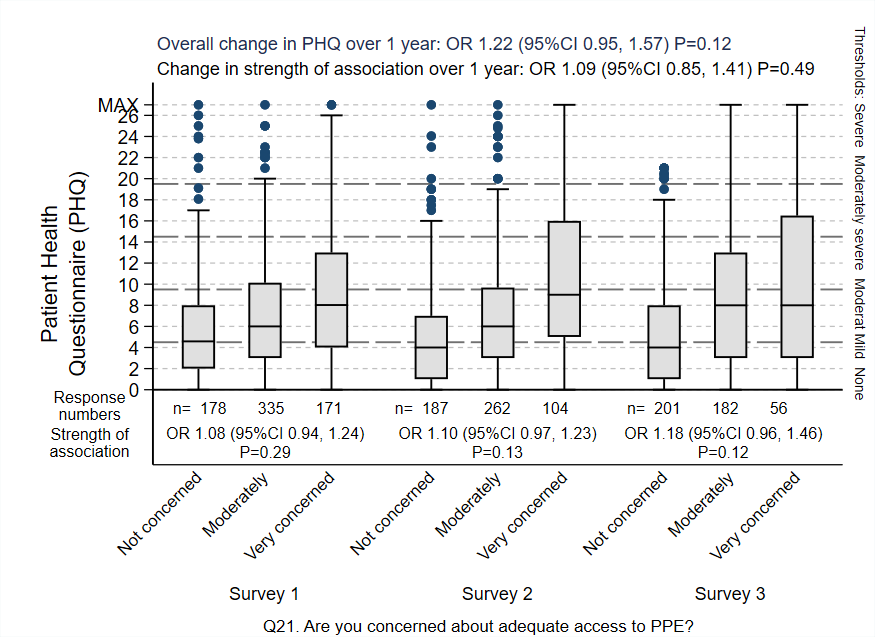

Supplement: S2 File — (ZIP) [file pone.0271824.s002.zip › ConPPE_PHQ9_V3.3_040522.tif]

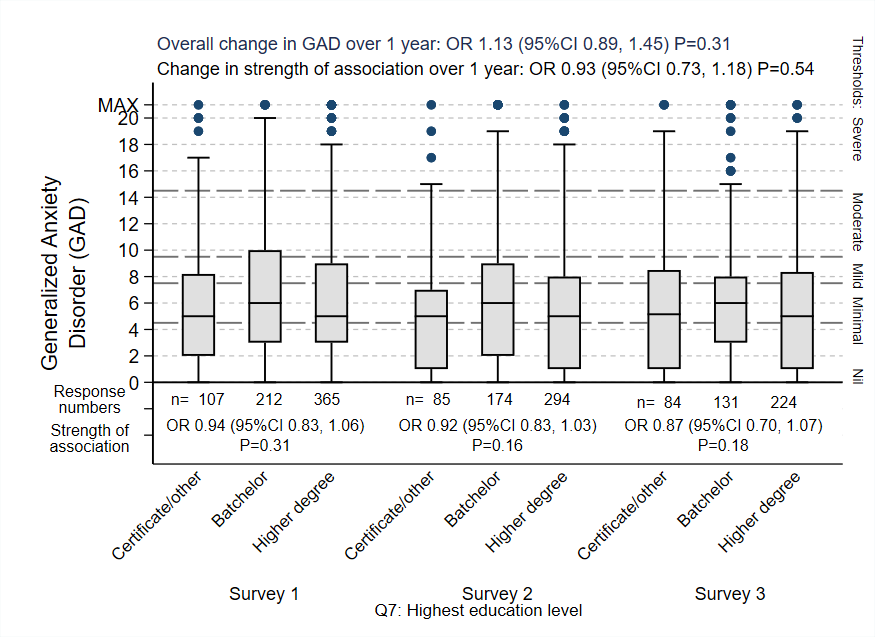

Supplement: S2 File — (ZIP) [file pone.0271824.s002.zip › Educ1_GAD7_V3.3_040522.tif]

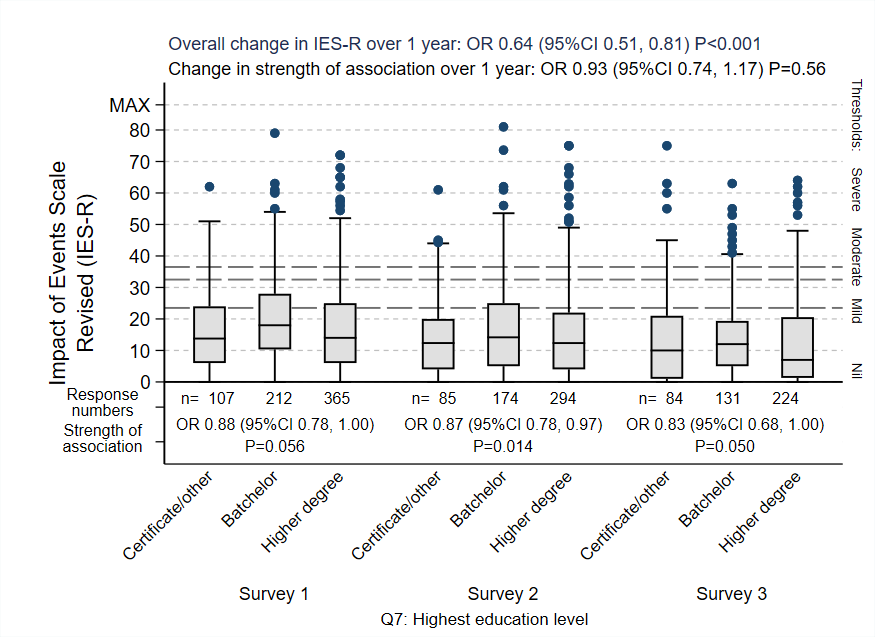

Supplement: S2 File — (ZIP) [file pone.0271824.s002.zip › Educ1_IESR22_V3.3_040522.tif]

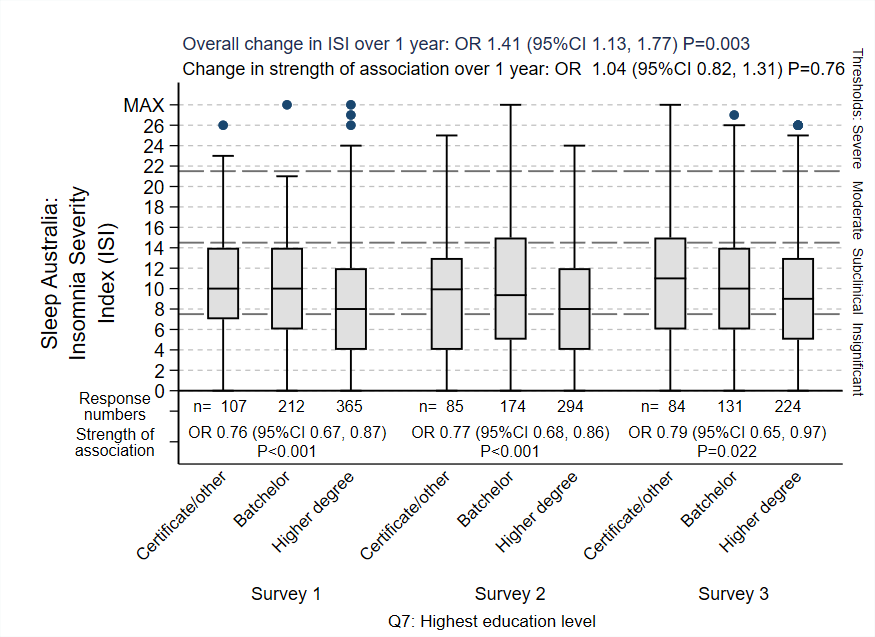

Supplement: S2 File — (ZIP) [file pone.0271824.s002.zip › Educ1_ISI7_V3.3_040522.tif]

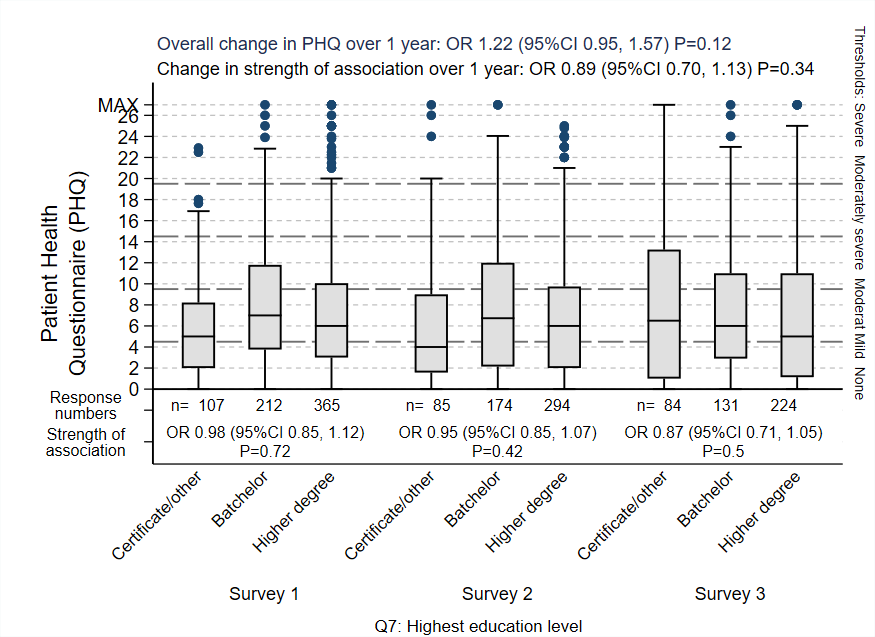

Supplement: S2 File — (ZIP) [file pone.0271824.s002.zip › Educ1_PHQ9_V3.3_040522.tif]

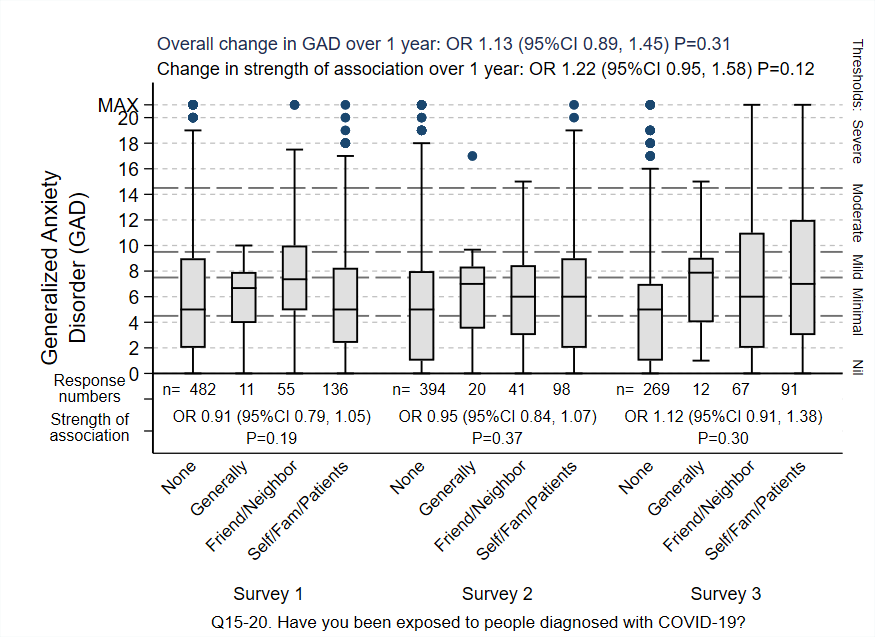

Supplement: S2 File — (ZIP) [file pone.0271824.s002.zip › ExpCv_Any2_GAD7_V3.3_040522.tif]

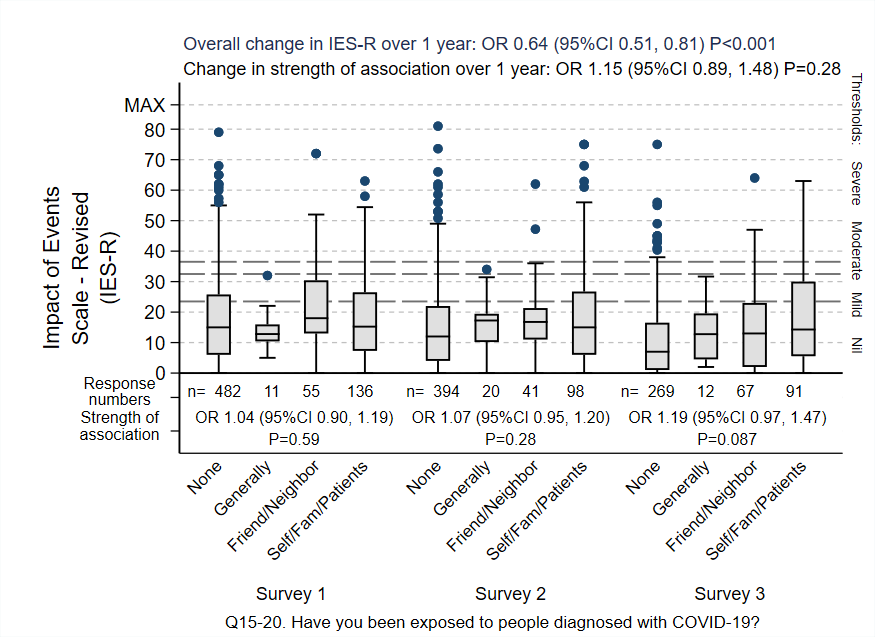

Supplement: S2 File — (ZIP) [file pone.0271824.s002.zip › ExpCv_Any2_IESR22_V3.3_040522.tif]

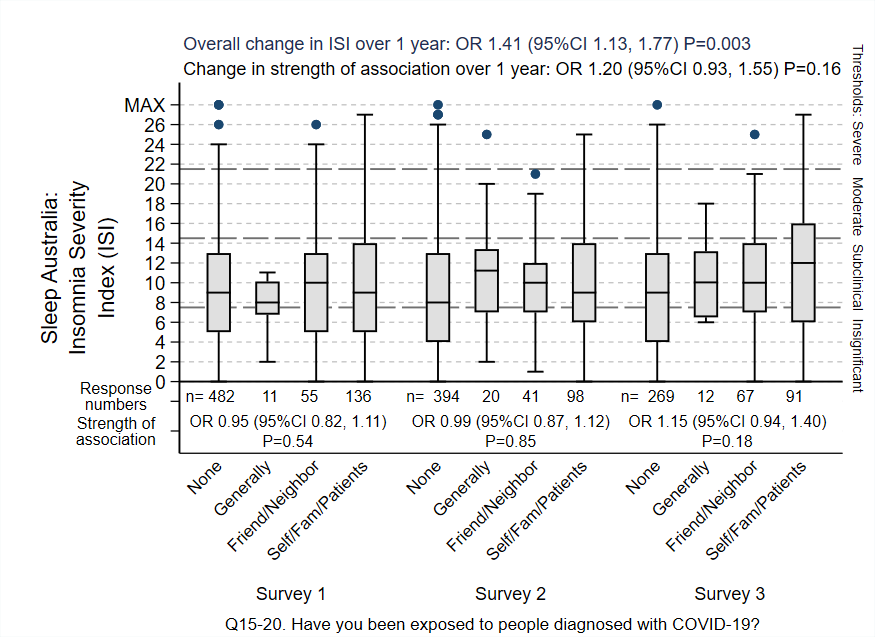

Supplement: S2 File — (ZIP) [file pone.0271824.s002.zip › ExpCv_Any2_ISI7_V3.3_040522.tif]

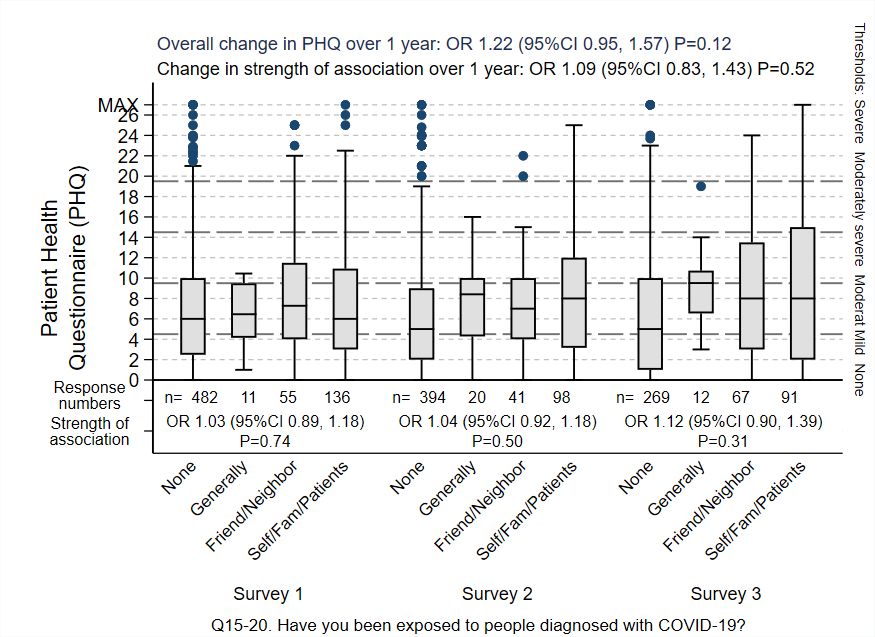

Supplement: S2 File — (ZIP) [file pone.0271824.s002.zip › ExpCv_Any2_PHQ9_V3.3_040522.tif]

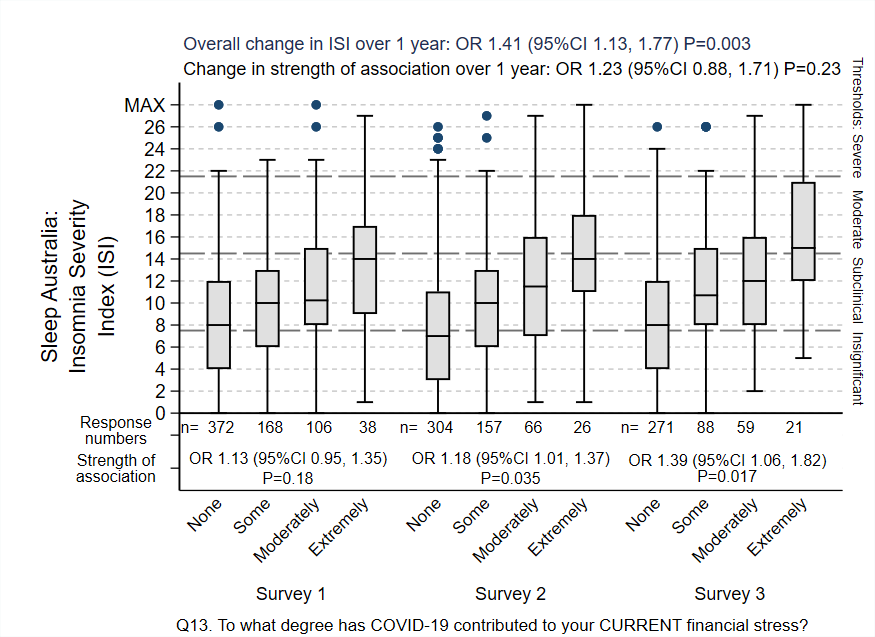

Supplement: S2 File — (ZIP) [file pone.0271824.s002.zip › FnSN13_ISI7_V3.3_040522.tif]

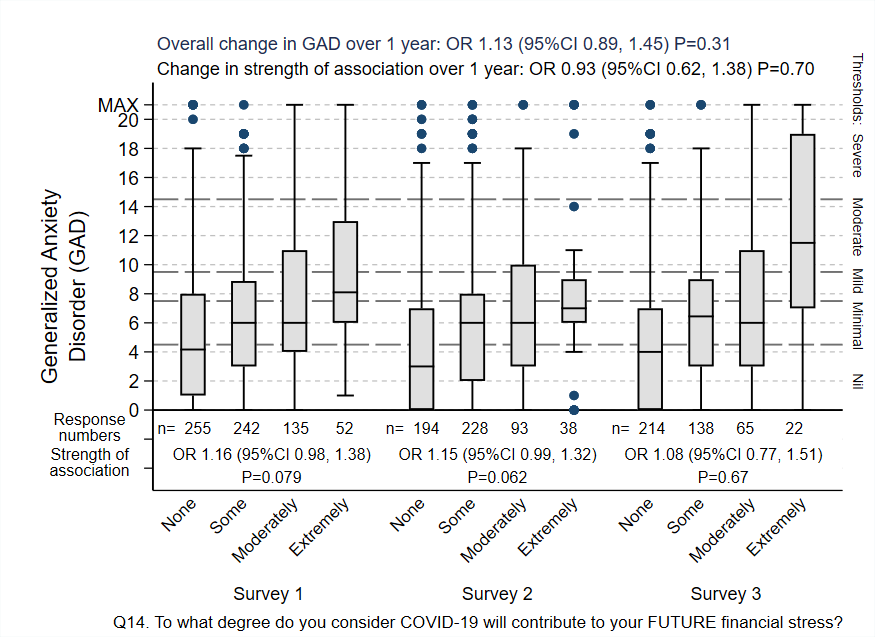

Supplement: S2 File — (ZIP) [file pone.0271824.s002.zip › FnSN14_GAD7_V3.3_040522.tif]

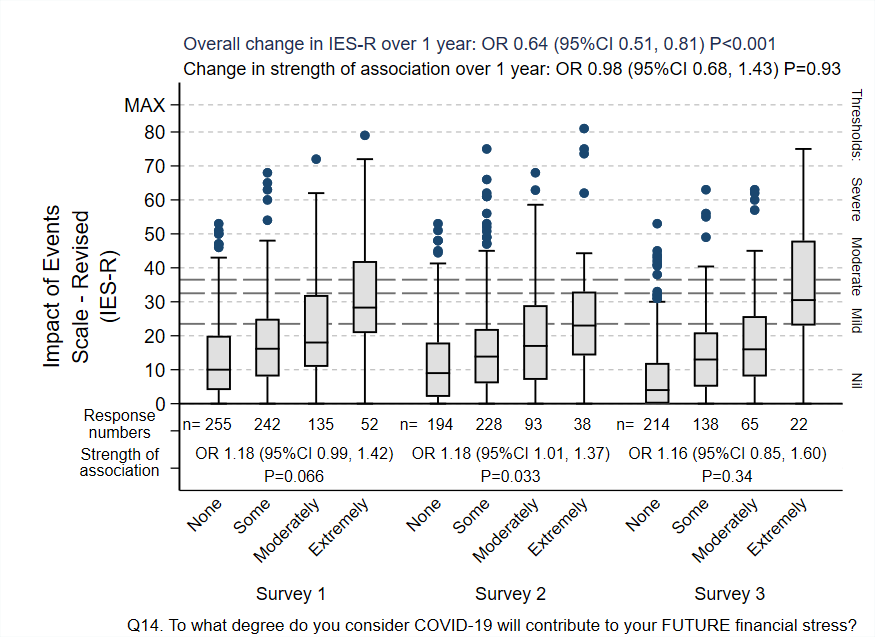

Supplement: S2 File — (ZIP) [file pone.0271824.s002.zip › FnSN14_IESR22_V3.3_040522.tif]

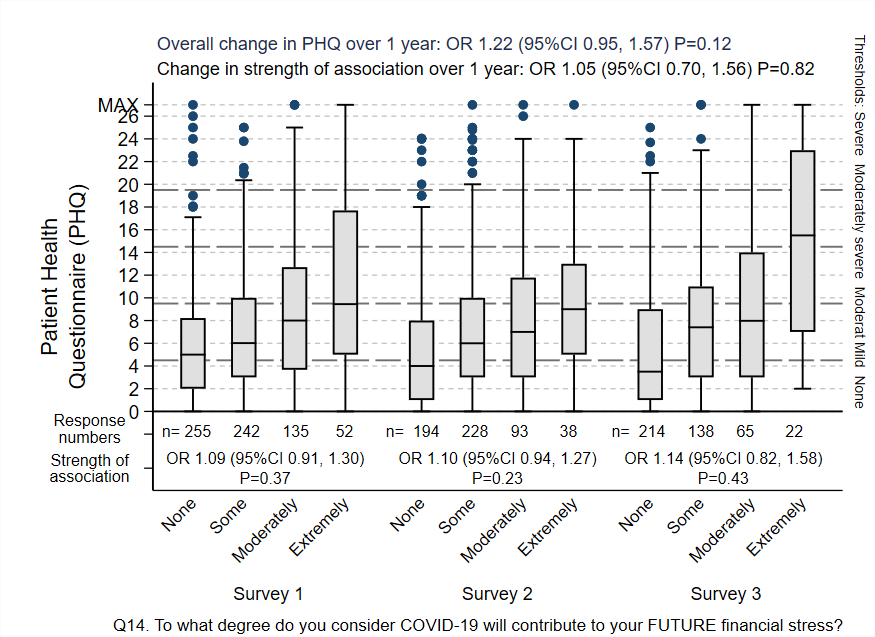

Supplement: S2 File — (ZIP) [file pone.0271824.s002.zip › FnSN14_PHQ9_V3.3_040522.tif]

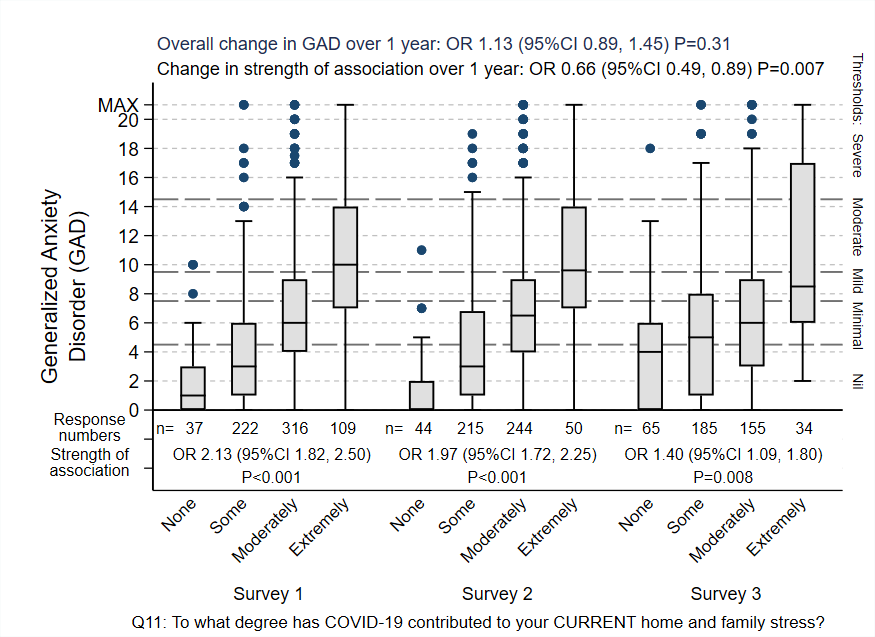

Supplement: S2 File — (ZIP) [file pone.0271824.s002.zip › HFSN11_GAD7_V3.3_040522.tif]

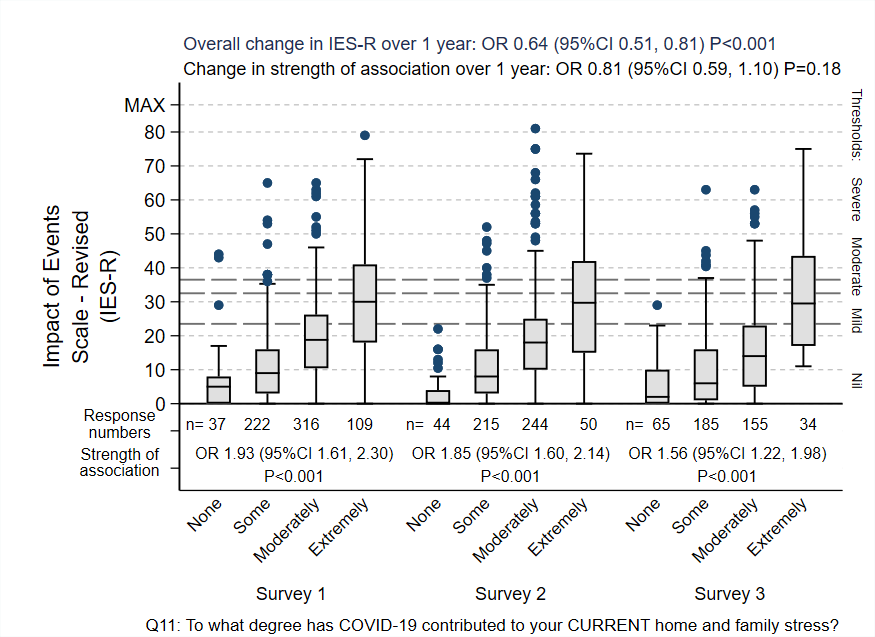

Supplement: S2 File — (ZIP) [file pone.0271824.s002.zip › HFSN11_IESR22_V3.3_040522.tif]

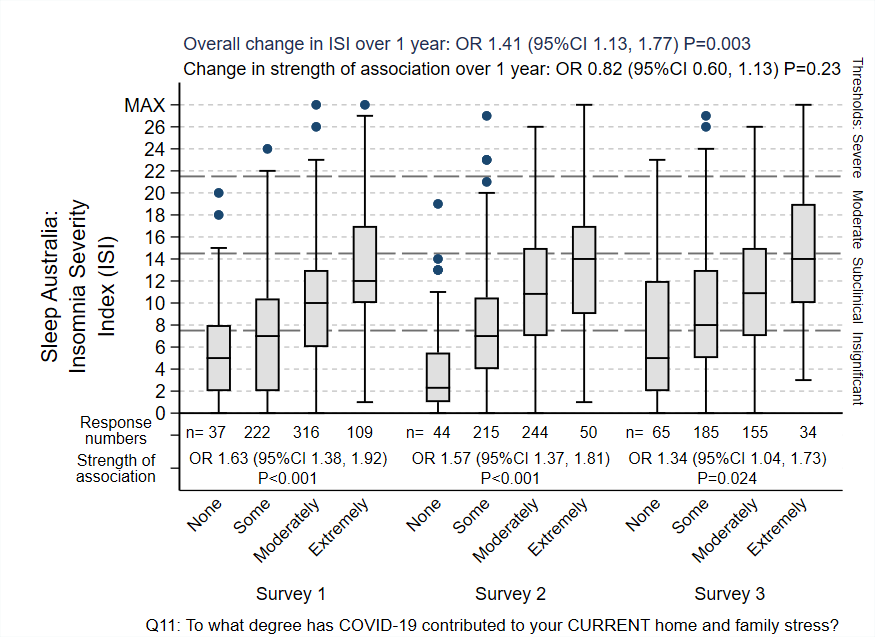

Supplement: S2 File — (ZIP) [file pone.0271824.s002.zip › HFSN11_ISI7_V3.3_040522.tif]

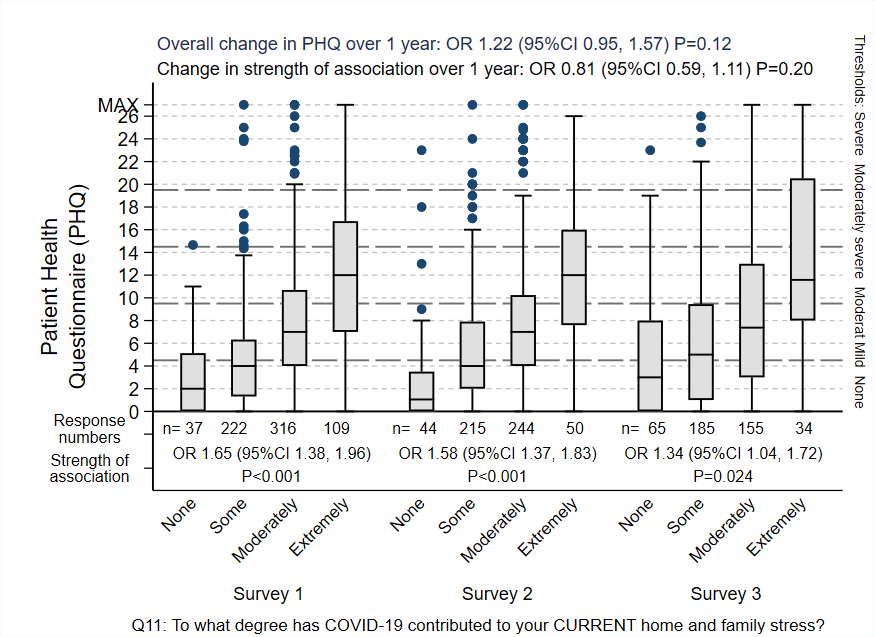

Supplement: S2 File — (ZIP) [file pone.0271824.s002.zip › HFSN11_PHQ9_V3.3_040522.tif]

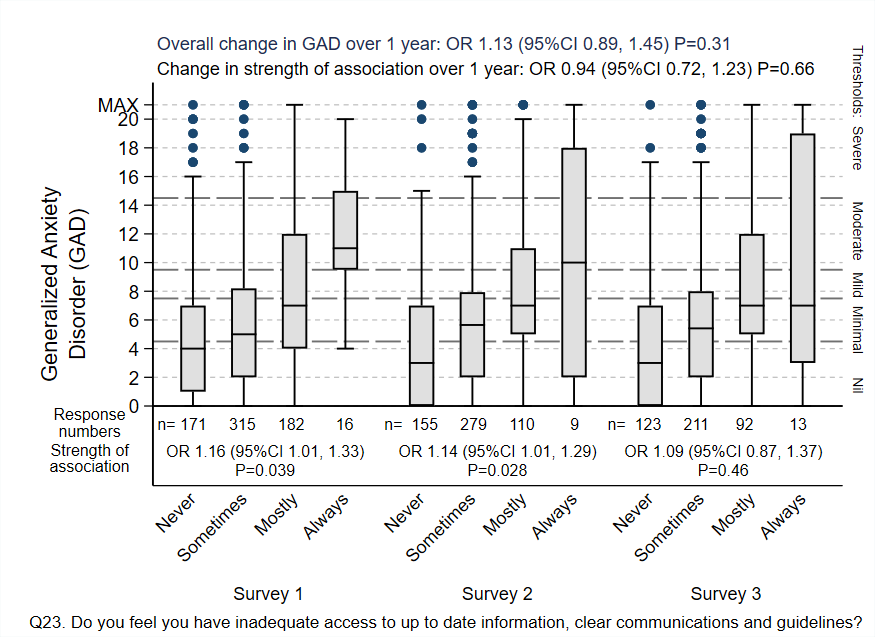

Supplement: S2 File — (ZIP) [file pone.0271824.s002.zip › InAdInfo23_GAD7_V3.3_040522.tif]

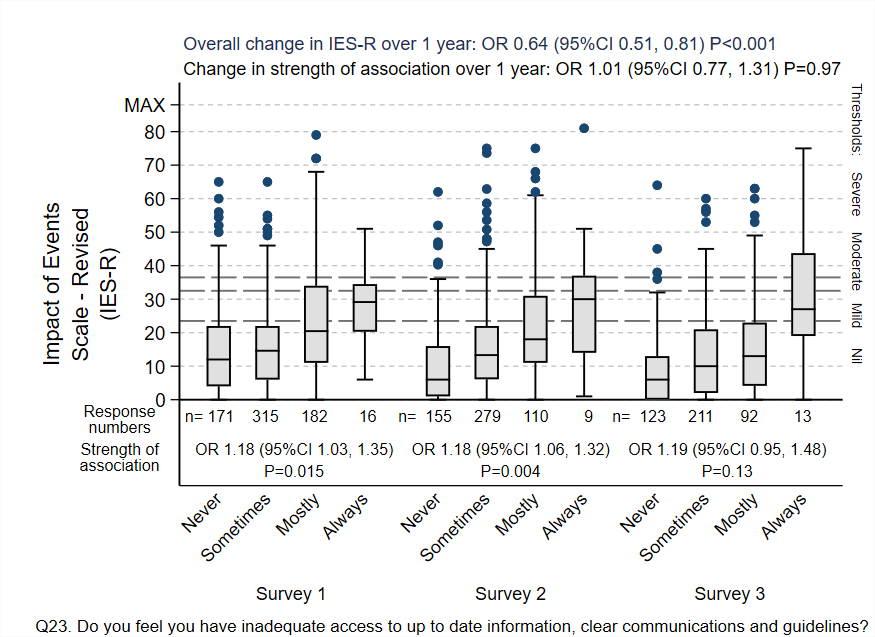

Supplement: S2 File — (ZIP) [file pone.0271824.s002.zip › InAdInfo23_IESR22_V3.3_040522.tif]

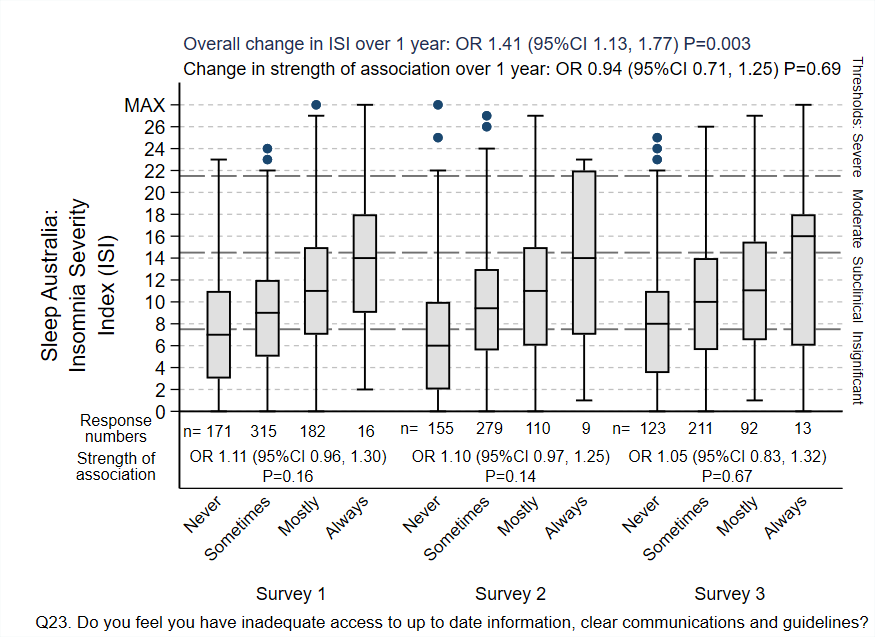

Supplement: S2 File — (ZIP) [file pone.0271824.s002.zip › InAdInfo23_ISI7_V3.3_040522.tif]

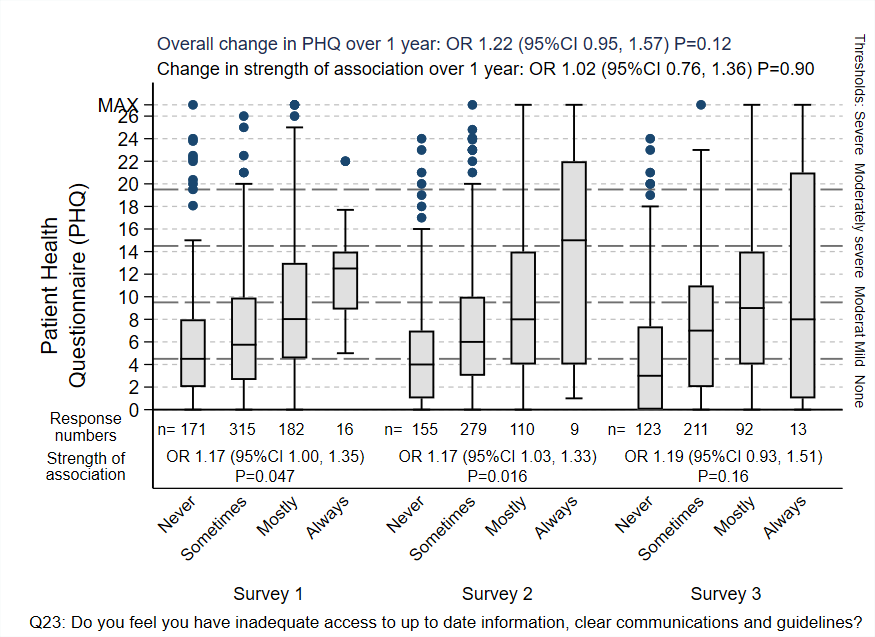

Supplement: S2 File — (ZIP) [file pone.0271824.s002.zip › InAdInfo23_PHQ9_V3.3_040522.tif]

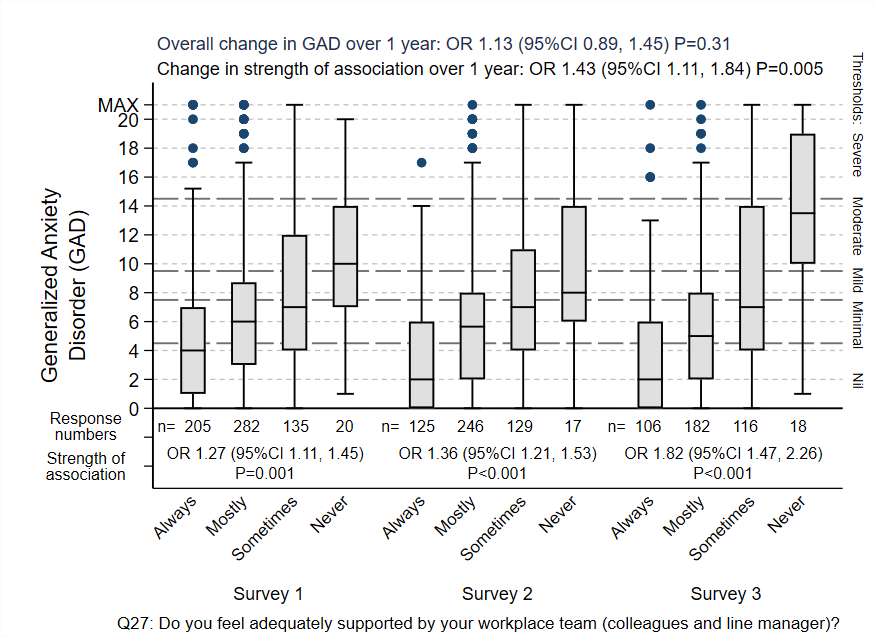

Supplement: S2 File — (ZIP) [file pone.0271824.s002.zip › STeam27_GAD7_V3.3_040522.tif]

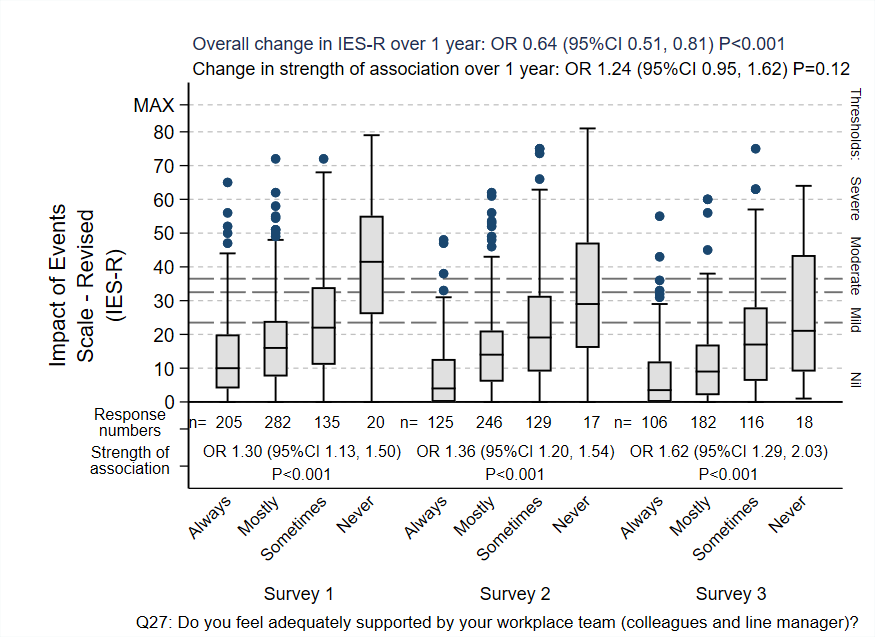

Supplement: S2 File — (ZIP) [file pone.0271824.s002.zip › STeam27_IESR22_V3.3_040522.tif]

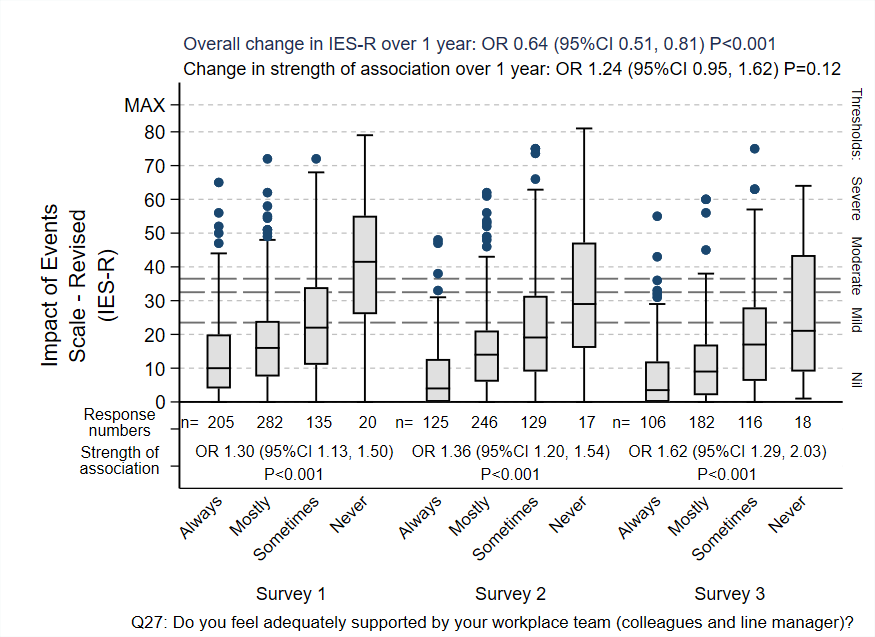

Supplement: S2 File — (ZIP) [file pone.0271824.s002.zip › STeam27_IESR22_V3.4_040522.tif]

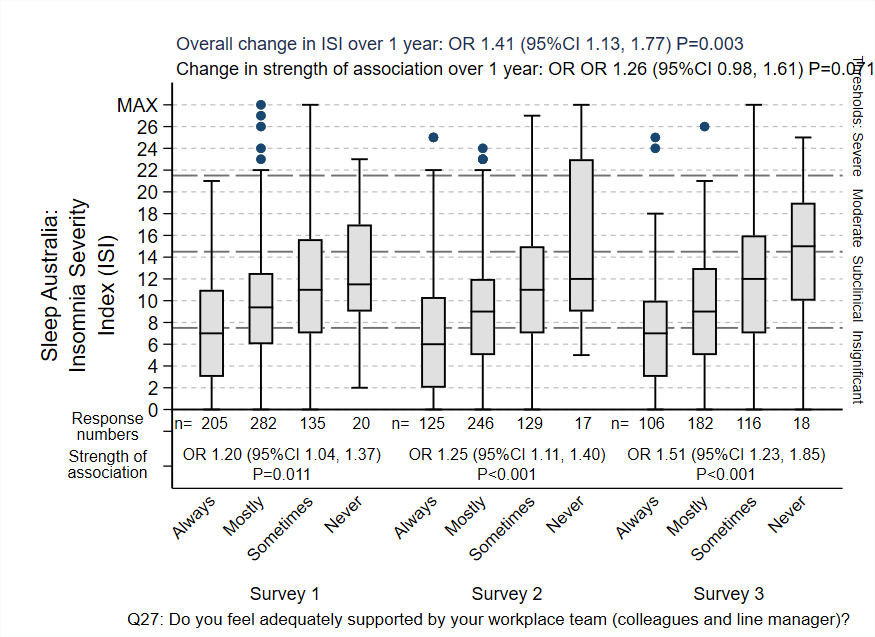

Supplement: S2 File — (ZIP) [file pone.0271824.s002.zip › STeam27_ISI7_V3.3_040522.tif]

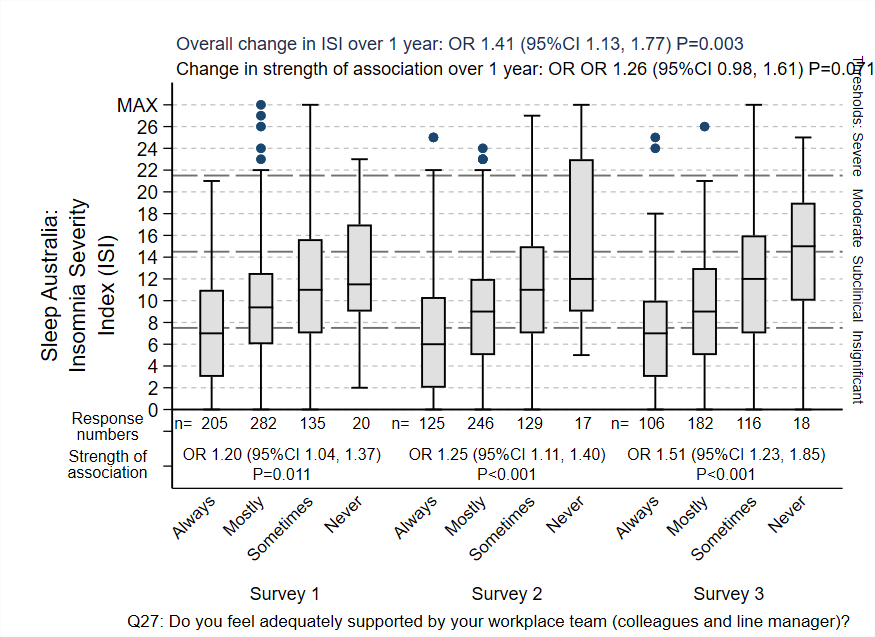

Supplement: S2 File — (ZIP) [file pone.0271824.s002.zip › STeam27_ISI7_V3.4_050522.tif]

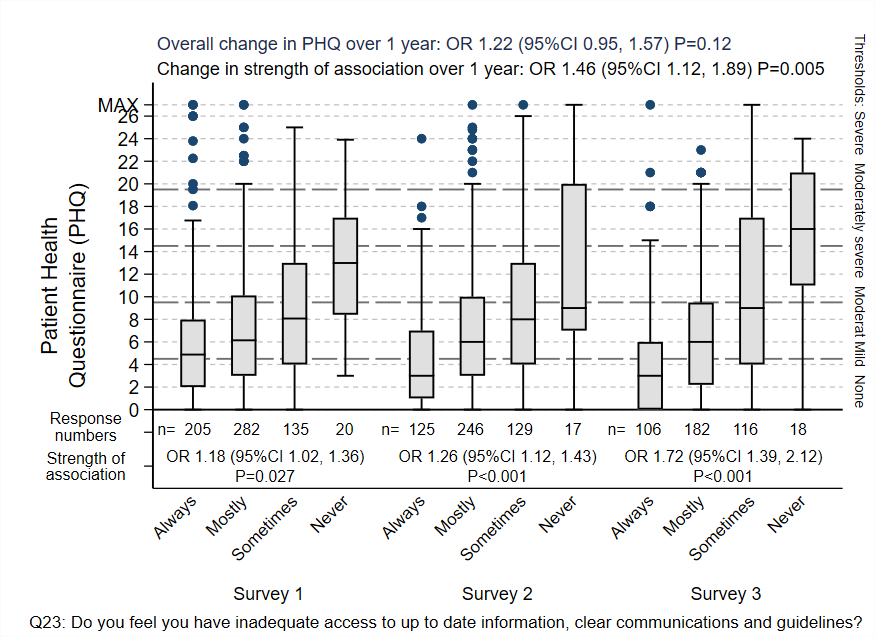

Supplement: S2 File — (ZIP) [file pone.0271824.s002.zip › STeam27_PHQ9_V3.3_040522.tif]

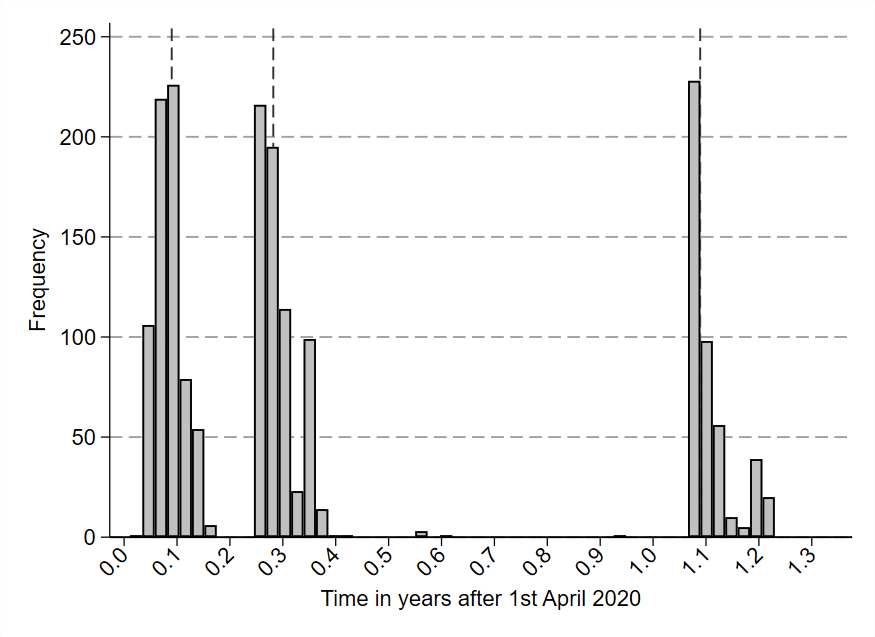

Supplement: S2 File — (ZIP) [file pone.0271824.s002.zip › Time_Respond_Histogram1_010522.tif]
