# Supplementary material for: Stressors, manifestations and course of COVID-19 related distress among public sector nurses and midwives during the COVID-19 pandemic first year in Tasmania, Australia
Source: PLoS One. 2022 Aug 9;17(8):e0271824. doi: 10.1371/journal.pone.0271824 (PMC9362919; doi:10.1371/journal.pone.0271824)
Supplement: S3 File — (DOCX) [file pone.0271824.s003.docx]

**Index of Additional Materials**

| **Material** | **Description** | **Pages** |
| --- | --- | --- |
|  | Respondent Flow Diagram | 2 |
| 1. | Additional Statistical Methodology | 3 – 27 |
| 1.1 | Population: identifiability of repeat responders | 2 – 3 |
| Table A1 | Assumed individuals who responded once, twice and thrice cumulatively at each Survey time-point. | 2 |
| 2. | Choice of statistical tests for effect size estimates of primary outcomes | 3 – 13 |
| 2.1 | Properties of the psychological outcome measurements | 3 – 5 |
| 2.1.1 | Analysis of the assumptions of linear regression in the main outcome analyses | 5 |
| Table A2 | Residual analysis of potential primary outcome multivariate regression analysis | 6 |
| 2.2 | Potential predictors of those outcomes | 6 |
| 2.2.1 | The numerical properties of the outcome and predictor variables were analysed | 7 |
| 2.2.2 | How the choices made of statistical tests were determined | 7 – 8 |
| 2.2.3 | Coding and transformation of predictor variables | 8 |
| Table A3 | Catalogue of potential predictor variables and their numerical properties | 9– 11 |
| 2.2.4 | Justification of the use of standardized normal transformations of predictor variables in regression models | 11 – 12 |
| 2.3 | Missing data reporting and handling | 12 – 13 |
| Table A4 | Tabulation of missing data | 12 |
| 2.2.6 | Primary regression analysis syntax | 13 |
| Table A5 | Explanation of how the syntax terms operate in Stata 16.1 as used in this analysis | 13 – 14 |
| 2.2.7 | Question 28 not included in the analysis | 14 |
| 3. | Additional Results | 15 – |
| 3.1 | Association of workplace variables and psychological outcomes | 15 – 17 |
| 3.1.1 | Figures: Q27. Do you feel adequately supported by your workplace team (colleagues and line manager)? | 15 |
| 3.1.2 | Figures: Q23. Do you feel you have inadequate access to up to date information, clear communications and guidelines? | 16 |
| 3.1.3 | Figures: Q21. Are you concerned about adequate access to PPE? | 16 |
| 3.1.4 | Figures: Q15 – Q20. Intensity of exposure | 17 |
| 3.2 | Association of home-life variables and psychological outcomes | 17 – 18 |
| 3.2.1 | Figures: Q11. To what degree has COVID-19 contributed to your CURRENT home and family stress? | 17 |
| 3.2.2 | Figures: Q14. To what degree do you consider COVID-19 will contribute to your Future financial stress? | 18 |
| 3.3 | Association of demographic variables and psychological outcomes | 18 – |
| 3.3.1 | Figures: Q7. Highest education level | 18 |
| 3.3.2 | Figures: Q8. What is your AGE GROUP? | 19 |
|  | Commentary on these additional results and their interpretation | 19 – 20 |
| Table A6 | Psychological test median scores, and IQR (%) in each classification range, plus missing data case numbers | 21 |
| 4. | Sensitivity analyses | 22 |
| 4.1 | Effect of more or fewer variables on effect size estimates: are the different predictor variables truly independent? Justification for inclusion of all predictor variables in final regression model | 23 – 27 |
| Tables A7 – A10 | Associations of predictors with GAD (A7), ISI (S8), IES-R (S9), PHQ (S10) in full and limited models: sensitivity analysis 1 results | 27 |
| 4.2 | Does the inability to accurately identify individuals who responded to more than one survey affect the predictor effect size estimates? | 27 |
| Tables 11 – 14 | Associations with GAD (A11), ISI (A12), IES-R (A13), PHQ (A14) in full models: with either exclusion of apparent duplicate responses (Group 1), or inclusion of all available responses (Group2): sensitivity analysis 2 results | 28 – 34 |

**Respondent Flow Diagram**





1. **Additional Statistical Methodology**

Our approach to the construction of an analytic framework for the survey of the impact on the psychological state of nurses in Tasmania of potentially causal factors, both general and specific to the Covid-19 pandemic was determined by a number of considerations.

- 1. **Population:** The participants in the survey were drawn from a sample from defined by the nursing department email list of the Tasmanian Health Service.

The people included in the data were those who chose to participate in the anonymous survey. There is no information about those who did not respond.

Individuals could not be identified accurately, and it was not possible to link responses from the same individual in the three survey periods. They were invited to create a code for themselves, but whether they wished to code themselves, or were willing and able to remember those codes accurately is unknown. The data appears to show that 1,303 individuals responded at some point, with 227 of those responding twice and 73 responding three times, producing 1,676 responses in total. It would seem unlikely that all the repeat responses were identified. The suggested code was crude, allowing the possibility that different individuals produced the same code by chance.

Table A1. Assumed individuals who responded once, twice and thrice cumulatively at each Survey time-point.

| Apparent response | Survey 1 | Survey 2 | Survey 3 | Total |
| --- | --- | --- | --- | --- |
| First response | 684 | 344 | 275 | 1303 |
| Second response | 0 | 209 | 91 | 300 |
| Third response | 0 | 0 | 73 | 73 |
| Total | 684 | 553 | 439 | 1676 |

For the analysis, the matching of individuals was assumed to be correct, and that failure to match individuals was taken as an inherent cost of undertaking any anonymous longitudinal online survey. Such failure to match may both under-estimate the effect sizes due to failure to compare each individual with themselves, and under-estimate the variances of those estimates since without accurate matching the observations are assumed to be independent.

For the future, it may be helpful if the commercial online survey tools were able to generate a study-specific random code during each episode of questionnaire completion, with a search of the already-completed responses to exclude repeats of the same code. The respondent would be asked to record that code in their mobile phone for future use (I place such codes as a dummy entry in my contacts list). This should not impose too great a burden on the commercial online corporations, or the memory of the respondents.

1. **Choice of statistical tests for effect size estimates of primary outcomes:**
   1. **Properties of the psychological outcome measurements:**

Standard validated measurement tools: Generalized Anxiety Disorder (GAD; range 0 – 21), Sleep Australia: Insomnia Severity Index (ISI; range 0 – 28), Impact of Events Scale – Revised (IES-R; range 0 – 88), Patient Health Questionnaire (PHQ; range 0 – 27).

These are inherently rank-ordered scales with varying ranges. Analytic methods that use linear regression methods (otherwise known as “parametric tests”) estimate means and mean differences that are relatively easily understood by readers. However, where the assumptions of linear regression are violated, the effect size estimates may be misleading, and may need to be avoided. Therefore, we tested those assumptions.

Patient Health Questionnaire (PHQ-9)

The patient health questionnaire (PHQ-9) is a 9-item measure of depression based on the diagnose criteria of DSM IV. The PHQ-9 has a dual-purpose to screen for the presence of a depressive disorder as well as to grade depressive symptom severity.

The PHQ-9 score ranges from 0 to 27, based on the 9 items scored from 0 = “not at all” to 3 = “nearly every day”. The suggested cut-points are 5, 10, 15, and 20, which represent the thresholds for mild, moderate, moderately severe, and severe depression, respectively[37]. Depression severity: 0-4 none, 5-9 mild, 10-14 moderate, 15-19 moderately severe, 20-27 severe. PHQ-9 shows adequate internal consistency at pre- and post-treatment, Cronbach’s alpha (α) = 0.74 and 0.81. The internal consistency for the PHQ-9 based on the current sample was α = 0.77.

General Anxiety Disorder (GAD-7)

General Anxiety Disorder (GAD-7) is a 7-item measure created as a screening tool for General Anxiety Disorder and has been used across various settings and populations [38-40] and has a range from 0-21. Anxiety severity ranges: 0-4 None to minimal, 5-7 mild (recommended to monitor symptoms), 8-9 mild though likely to be diagnosed with an anxiety disorder, 10-14 moderate symptoms are clinically significant, 15-21 severe symptoms warrant active treatment. A cut-off score of 10 has been identified as the optimal point for sensitivity (89%) and specificity (82%) [ Kroenke K., S.R., Williams J., Monahan P., Löwe B., *Anxiety disorders in primary care: Prevalence, impairment, comorbidity, and detection.* Annals of Internal Medicine, 2007. **146**: p. 317-325.]. GAD-7 has demonstrated strong psychometric properties in the general population and adequate internal consistency across subgroups (α = 0.89). The internal consistency of the GAD-7 based on the current sample was α = 0.89.

Insomnia Severity Index (ISI)

The Insomnia Severity Index (ISI)[Wilson J., K.T., *Assessing psychological trauma and PTSD*. 2004, New York: Guilford Press] consists of 7 items to assesses the nature, severity, and impact of insomnia. Items include severity of sleep onset, sleep maintenance, early morning awakening problems, sleep dissatisfaction, interference of sleep difficulties with daytime functioning, noticeability of sleep problems by others, and distress caused by the sleep difficulties. A 5-point rating scale is used to rate each item, with 0 = no problem and 4 = very severe problem, yielding a total score ranging from 0 to 28. The total score is interpreted as follows: absence of insomnia (0–7), sub-threshold insomnia (8–14), moderate insomnia (15–21), and severe insomnia (22–28) [ Bastien C., V.A., Morin C., *Validation of the insomnia severity index as an outcome measure for insomnia research.* Sleep Medicine, 2001. **2**: p. 297-307]. The ISI has been evaluated in a population-based sample and the internal consistency was excellent, α = 0.90. It is suggested a cut-off score of 10 (86.1% sensitivity and 87.7% specificity) for detecting insomnia in a general population [ Morin C., B.G., Belanger L., Ivers H., *The insomnia severity index: Psychometric indicators to detect insomnia cases and evaluate treatment response.* 2011. **34**: p. 601-608]. The internal consistency for the ISI-7 in the present study was α = 0.90.

­­­­­­­­­­­­­­­­­­­Impact of Events Scale-Revised (IES-R)

PTSD often coexists and interacts with anxiety and depression, and contributes to delayed recovery, suboptimal functional outcome, poor quality of life, sleep disorders and feelings of being detached. The IES-R provides a dimensional assessment of PTSD. Participants specify the frequency with which they have had intrusion-, avoidance-, and hyperarousal- related thoughts in the previous 7 days on a Likert scale. The scores for the intrusion component of the scale range from 0-24, for the avoidance component, 0-32 and hyperarousal 0-24 with a total score between 0-88. The higher the score, the greater the level of distress indicated. For the identification of coping disorders, scores above the cut-off point of 35 were classified as a high level of PTSD-related symptoms[ Neal L., B.W., Rollins J., Herepath R., Strike P., *Convergent validity of measures of post traumatic stress disorder in mixed military and civilian population.* J Trauma Stress, 1994. **7**: p. 447-55].

Weiss and Marmar report [Weiss D. MC. *The Impact of Events Scale Revised: Assessing Psychological Trauma and PTSD*. New York, Guilford Press; 1997] the IES-R showed high internal consistency, with Cronbach’s alphas ranging from 0.87-0.92 for intrusion, 0.84-0.85 for avoidance and 0.79-0.90 for hyperarousal. Test-retest correlation coefficients ranged from 0.57-0.94 for intrusion, 0.51-0.89 for avoidance and 0.59-0.92 for hyperarousal. The IES-R has been used in numerous studies with a wide variety of adult populations and has proved valuable in documenting the course of posttraumatic phenomena over time[ Rothbaum B., F.E., Riggs D., Murdock T., Walsh W., *A prospective examination of post traumatic stress disorder in rape victims.* Journal of Traumatic Stress, 1992. **7**: p. 669-90].

- - 1. **Analysis of the assumptions of linear regression in the main outcome analyses**

Where possible, multivariate regression analysis is performed using linear regression methods, since this uses the maximum amount of the value of the data that is available. However, where the distribution of residuals from the regression models (the variation in values left over after the predictions of the observed values associated with the predictor variables included in the model have been removed, otherwise known as residual analysis) violate assumptions of linear regression, the effect size estimates from the model may not be reliable. In which case, it may be prudent to use rank-ordered analyses (sometimes called “non-parametric” analyses); although these analyses are less informative, they are less likely to give an inaccurate answer in terms of relative magnitude and direction of effect.

The assumptions of linear regression tested here are

- - - 1. Cameron & Trivedi's decomposition of IM-test (Information Matrix Test) tests that
         1. There is No heteroskedasticity (the standard deviation of the residuals is the same all along the x-axis)
         2. No skewness (residual values more to one side or other of the mean)
         3. No kurtosis (residuals more bunched together of spread out than expected from a normal distribution)
      2. Ramsey RESET test that there is no need to raise any of the predictor variables to a power relationship to achieve optimum model fit.
      3. Residual plots (residuals against predicted values) were inspected for any distortions to their distributions.

Post-estimation testing of the primary regression models using multilevel mixed effects linear regression of the association of outcome measures and the predictor variables being examined was performed. This is the closest approximation to performing optimum parametric analysis that would allow this residual analysis. Missing variables were substituted using the mean values of the 20 imputations created for the primary multiple imputation ordered logistic regression analysis for each outcome (post-estimation testing in multiple imputation analyses in Stata 16.1 does not include residual analysis);

Table A2. shows strong violations of the assumptions of linear regression, and also a degree of truncation of the expected random scatter of residual values. Logarithmic transformation of the outcome variables did not significantly improve the analysis. Therefore, the results of linear regression analyses may be unreliable, and we have chosen ordered logistic regression as a “non-parametric” equivalent of the necessary multivariate linear regression models.

Table A2. Residual analysis of potential primary outcome multivariate regression analysis

| GAD7 |  |  |  | 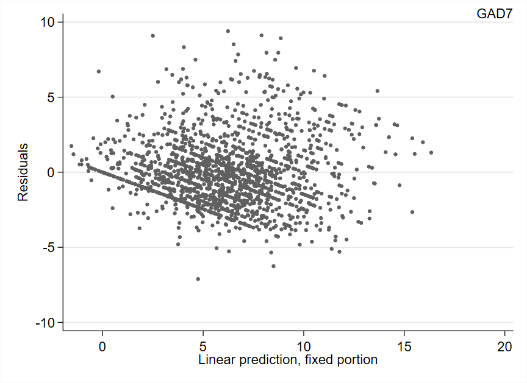 |
| --- | --- | --- | --- | --- |
| Imtest | Chi2 | df | P-value |  |
| Heteroskedasticity | 38.7 | 2 | <0.0001 |  |
| Skewness | 67.58 | 1 | <0.0001 |  |
| Kurtosis | 21.46 | 1 | <0.0001 |  |
| Total | 127.73 | 4 | <0.0001 |  |
|  | F-test |  | P-value |  |
| Ramsey RESET test | F(3, 1671) | 7.15 | 0.0001 |  |
| ISI7 |  |  |  | 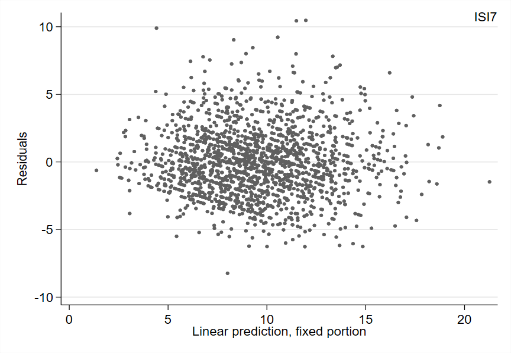 |
| Imtest | Chi2 | df | P-value |  |
| Heteroskedasticity | 11.09 | 2 | 0.0039 |  |
| Skewness | 28.63 | 1 | <0.0001 |  |
| Kurtosis | 8.08 | 1 | 0.0045 |  |
| Total | 47.79 | 4 | <0.0001 |  |
|  | F-test |  | P-value |  |
| Ramsey RESET test | F(3, 1671) | 0.26 | 0.86 |  |
| IESR22 |  |  |  | 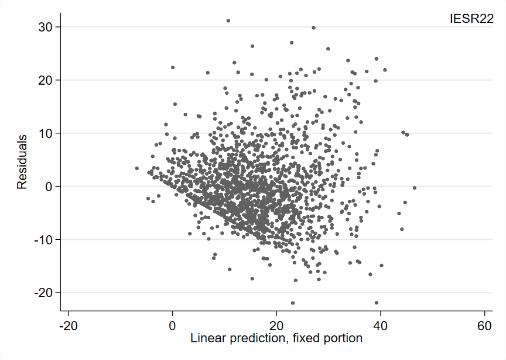 |
| Imtest | Chi2 | df | P-value |  |
| Heteroskedasticity | 129.86 | 2 | <0.0001 |  |
| Skewness | 68.63 | 1 | <0.0001 |  |
| Kurtosis | 20.32 | 1 | <0.0001 |  |
| Total | 218.81 | 4 | <0.0001 |  |
|  | F-test |  | P-value |  |
| Ramsey RESET test | F(3, 1671) | 12.46 | <0.0001 |  |
| PHQ9 |  |  |  | 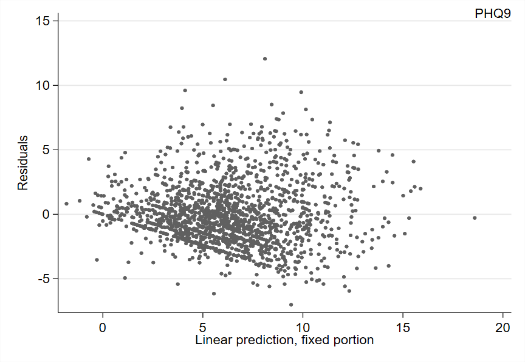 |
| Imtest | Chi2 | df | P-value |  |
| Heteroskedasticity | 73.7 | 2 | <0.0001 |  |
| Skewness | 53.96 | 1 | <0.0001 |  |
| Kurtosis | 10.3 | 1 | 0.0013 |  |
| Total | 137.96 | 4 | <0.0001 |  |
|  | F-test |  | P-value |  |
| Ramsey RESET test | F(3, 1671) | 3.64 | 0.0123 |  |

Information matrix test for the regression model and an orthogonal decomposition into tests for heteroskedasticity, skewness, and kurtosis due to Cameron and Trivedi. Ramsey RESET test for missing variables: whether power versions of the predictor variables appear to be operating

- 1. **Potential predictors of those outcomes**

The relationship of the predictors to the outcomes was investigated by estimating the relative strength of those associations. The purpose of the statistical tests was to **estimate effect sizes**, **estimate the variability** of those effect size measurements as assessed by **95% confidence intervals** (defining the likely position of the true population effect sizes), with **P-values** defining the position of those effect sizes along the assumed distribution of the variability of the measurements. No prior assumptions were made about any of the effect sizes or distributions. The nature of the statistical tests used to perform the estimations was determined by:

- - 1. **The numerical properties of the outcome and predictor variables were analysed, shown below in Table A3.**

There is a specific problem with the survey predictor measurement scales; are the values being measured the same in different individuals. This is to some extent unavoidable. The outcome measurements are at least validated in the usual way for such tools. The predictor measurements, however, are not validated in the same way. Each respondent makes their own judgement about matching the words in the questions with what they feel about the quality/quantity being asked. There is no reason why different respondents will be making quantitatively comparable judgements, although such comparability is implicit in the study design. What might be assumed is that each individual will be making a similar judgement about the different questions, and that individual respondents are doing some sort of work to allow the Likert scales to be compared.

In addition, there was a significant amount of missing data (see section below). The likely cross-correlations between the measured and unmeasured predictor variables leading to identifiable and unidentifiable confounding effects. There is a need to avoid distortion of the results of the estimations by these considerations.

The multiple predictor variables dictated that multivariate regression models be used as the statistical tests. The requirements of the choice of the statistical tests were that they could accommodate:

- - - 1. Rank-ordered measurement scales
      2. Variable distributions that violate the assumptions of linear regression
      3. Repeated measurements in the same participants, at least to the extent that it was possible to identify individual from an anonymous survey
      4. Presentation of the predictor scales in a way that allowed valid comparison of the relative strength of the association between outcomes and predictors (which predictors were more important in influencing the psychological states of the nurses in the survey)
      5. Significant amounts of missing data
      6. The statistical tests chosen must be available in Stata 16.1, the statistical package available to the research team.
    1. **The choices made of statistical tests were determined by (the terminology used here is that used by Stata to describe its techniques):**
       1. The quantity of missing data dictated that multiple imputation methodology be used to account for that missing data.
       2. It would have been preferable to use multi-level mixed effects linear regression, as this methodology takes account of repeated-measures and random effects that might otherwise distort the effect size estimates. However, tests of the associations between outcomes and predictors showed strong violation of the assumptions of linear regression as shown by regression residual analysis (Table A2).
       3. Thus, a **rank-ordered repeated-measures multivariate regression** (ologit) methodology was required that could be implemented with **multiple imputation** methods in Stata. A mixed effects version of ologit methods is available in Stata, but this is not implementable by multiple imputation.
       4. The simplest method that fulfilled these requirements was **ordered-logistic regression** with missing data substituted with **multiple imputation**.
       5. This produces effect size estimates as beta-coefficients that are converted into **odds ratios** by exponential expansion. This is not ideal, since interpretation of odds ratios may not be familiar to all of the target audience for this paper. However, odds ratios will not be totally foreign to the audience, and statistical tests are likely to be the least unreliable method of comparison.
    2. **Coding and transformation of predictor variables:**

The direct comparison between the strength of the association between the different measured predictors of the outcome was made by including standardized normal transformations of the predictor variables.

Thus, all the predictor variables in the regression model are converted into the same standardized measure of the variability of those predictors. This accounts to a certain extent for the different measurement scales and variability of the data: it was a pragmatic choice in the face of potential incompatibility between the different measurement processes employed in the survey.

The use of odds ratio also deals with the problem of potential lack of comparability between the judgements of different subjective experiences of respondents in how they score the predictor variable questions. Each respondent is probably using their own experiential scales to compare the different predictor responses to the survey questions. A precise matching of the effect size measurement scales with the predictor question scales may lose sight of this process in the face of poor inter-respondent comparisons.

Thus, the effect size odds ratio estimates may give a more reliable measure of the direction of the association, and the relative size of the effects of different predictors, but not any reliable estimate of the absolute effect sizes of the individual predictors.

However, the predictor measurements were not constructed prior to the decision to conduct the survey, but were chosen to suit the needs of the impact of the Covid-19 pandemic on the psychological health of nurses in Tasmania. They were not validated measurement tools.

In an ideal situation, a quantitative survey would be preceded by a qualitative investigation of what are the most important predictors of the outcome measures in the survey. Qualitative methods would investigate the possible predictors of distress, and validated tools for accurate measurement of those predictors constructed with numerical properties designed for appropriate statistical analysis. There was no time to do so. It was determined that the first survey would start within 2 weeks of the acknowledgement by the Australian government of the existence of the Covid-19 pandemic, allowing no time for the careful development of the questionnaire questions. The same questionnaire was to be repeated a 3-months and 12-months. Thus, the questions to be asked, and the responses sought, were chosen quickly, without careful planning of the statistical analysis of the data that was collected.

Table A3. Catalogue of predictor variables and their numerical properties

|  |  |  | | Respondent numbers | | | | For z-score | |
| --- | --- | --- | --- | --- | --- | --- | --- | --- | --- |
|  | Predictor | Coding | | Survey 1 | Survey 2 | Survey 3 | Total | Mean | SD |
| Q2 | Staff grade | | All | 676 | 539 | 431 | 1646 | 1.25 | 0.65 |
|  | State registered nurse | | 1 | 572 | 473 | 362 | 1407 |  |  |
|  | State registered midwife | | 2 | 40 | 26 | 26 | 92 |  |  |
|  | State enrolled nurse | | 3 | 56 | 32 | 38 | 126 |  |  |
|  | Assistant in nursing | | 4 | 8 | 8 | 5 | 21 |  |  |
|  | [“Others” allocated to the nearest similar employment class] | |  |  |  |  | [44] |  |  |
| Q3 | Covid ward status | | All | 667 | 538 | 388 | 1631 | 0.17 | 0.48 |
|  | Covid-negative patients | | 0 | 547 | 487 | 388 | 1422 |  |  |
|  | Both | | 1 | 41 | 20 | 13 | 74 |  |  |
|  | Covid-positive patients | | 2 | 79 | 31 | 25 | 135 |  |  |
| Q5 | Sector | | All | 377 | 538 | 433 | 1348 | 0.96 | 0.21 |
|  | Public | | 1 | 359 | 517 | 412 | 1288 |  |  |
|  | Private/Both/Other | | 0 | 18 | 21 | 21 | 60 |  |  |
| Q6 | Gender | | All | 670 | 538 | 433 | 1641 | 0.12 | 0.33 |
|  | Female | | 0 | 590 | 462 | 390 | 1442 |  |  |
|  | Male | | 1 | 80 | 76 | 43 | 199 |  |  |
| Q7 | Highest education level | | All | 670 | 539 | 433 | 1642 | 1.36 | 0.75 |
|  | Hospital certificate | | 0 | 61 | 54 | 47 | 162 |  |  |
|  | TAFE diploma | | 0 | 35 | 25 | 33 | 93 |  |  |
|  | Undergraduate student | | 0 | 9 | 6 | 4 | 19 |  |  |
|  | Batchelor degree | | 1 | 210 | 171 | 130 | 511 |  |  |
|  | Postgrad certificate/Masters/PhD | | 2 | 355 | 283 | 219 | 857 |  |  |
| Q8 | Age group (years) | | All | 675 | 538 | 432 | 1645 | 4.08 | 1.34 |
|  | 18-25 | | 1 | 37 | 26 | 22 | 85 |  |  |
|  | 26-30 | | 2 | 56 | 36 | 30 | 122 |  |  |
|  | 30-40 | | 3 | 138 | 104 | 88 | 330 |  |  |
|  | 41-50 | | 4 | 134 | 123 | 88 | 345 |  |  |
|  | 51-60 | | 5 | 234 | 186 | 151 | 571 |  |  |
|  | 61-71 | | 6 | 75 | 61 | 51 | 187 |  |  |
|  | 71+ | | 7 | 1 | 2 | 2 | 5 |  |  |
| Q9 | Social situation | | All | 449 | 527 | 429 | 1405 | 1.20 | 0.46 |
|  | Living with partner/family/friends | | 1 | 354 | 410 | 335 | 1159 |  |  |
|  | Living with friends | | 2 | 11 | 15 | 8 | 34 |  |  |
|  | Living caring for others | | 3 | 19 | 24 | 17 |  |  |  |
|  | Living alone | | 4 | 65 | 78 | 69 | 212 |  |  |
| Q10 | Smoking | | All | 664 | 491 | 392 | 1619 | 0.078 | 0.27 |
|  | Non-smoker | | 0 | 609 | 491 | 392 | 1492 |  |  |
|  | Smoker | | 1 | 55 | 40 | 32 | 127 |  |  |
| Q11 | Current home/family stress | | All | 664 | 534 | 430 | 1628 | 1.56 | 0.81 |
|  | Not at all | | 0 | 37 | 44 | 65 | 146 |  |  |
|  | Some | | 1 | 220 | 210 | 185 | 615 |  |  |
|  | Moderately | | 2 | 298 | 230 | 146 | 674 |  |  |
|  | Extremely | | 3 | 109 | 50 | 34 | 193 |  |  |
| Q12 | Future home/family stress | | All | 618 | 506 | 416 | 1540 | 1.50 | 0.75 |
|  | Not at all | | 0 | 40 | 30 | 44 | 114 |  |  |
|  | Some | | 1 | 256 | 205 | 219 | 680 |  |  |
|  | Moderately | | 2 | 261 | 229 | 124 | 614 |  |  |
|  | Extremely | | 3 | 61 | 42 | 29 | 132 |  |  |

Table A3. (Cont.) Catalogue of predictor variables and their numerical properties

|  |  |  | | Respondent numbers | | | | | | For z-score | | | | | |  |
| --- | --- | --- | --- | --- | --- | --- | --- | --- | --- | --- | --- | --- | --- | --- | --- | --- |
|  | Predictor | Coding | | Survey 1 | Survey 2 | Survey 3 | | Total | Mean | | | SD | | |  |  |
| Q13 | Current financial stress | | All | 658 | 530 | 430 | | 1618 | 0.67 | | | 0.91 | | |  |  |
|  | Not at all | | 0 | 368 | 299 | 269 | | 936 |  | | |  | | |  |  |
|  | Some | | 1 | 146 | 139 | 81 | | 366 |  | | |  | | |  |  |
|  | Moderately | | 2 | 106 | 66 | 59 | | 231 |  | | |  | | |  |  |
|  | Extremely | | 3 | 38 | 26 | 21 | | 85 |  | | |  | | |  |  |
| Q14 | Future financial stress | | All | 587 | 488 | 402 | | 1477 | 0.91 | | | 0.93 | | |  |  |
|  | Not at all | | 0 | 226 | 184 | 198 | | 608 |  | | |  | | |  |  |
|  | Some | | 1 | 189 | 186 | 121 | | 496 |  | | |  | | |  |  |
|  | Moderately | | 2 | 123 | 85 | 61 | | 269 |  | | |  | | |  |  |
|  | Extremely | | 3 | 49 | 33 | 22 | | 104 |  | | |  | | |  |  |
| Q15 - 20 | Intensity of exposure to Covid | | All | 660 | 532 | 432 | | 1624 | 0.63 | | | 1.06 | | |  |  |
|  | None | | 0 | 477 | 392 | 269 | | 1138 |  | | |  | | |  |  |
|  | Exposed (not defined) | | 1 | 46 | 37 | 55 | | 138 |  | | |  | | |  |  |
|  | Exposed friends/neighbours | | 2 | 52 | 39 | 66 | | 157 |  | | |  | | |  |  |
|  | Exposed family/patients | | 3 | 85 | 64 | 42 | | 191 |  | | |  | | |  |  |
|  | Self was diagnosed with Covid | | 4 | 0 | 0 | 0 | | 0 |  | | |  | | |  |  |
|  | The hierarchy of Covid exposure intensity was hypothesized by the rank in this list | | 0  1  2  3  4  Missing | Q15-Q20 No  Q15 Yes, but No to other questions ( Q16-Q20)  Q19 or Q20 Yes, but No to Q16-Q18  Q17 or Q18 Yes, but No to Q16  Q16 Yes  “Unsure”, “Prefer not to answer”, Not answered | | | | | | | | | |  |  |  |
| Q21 | Concerned about PPE | | All | 648 | 522 | 425 | 1595 | | | | 1.86 | | 0.81 | | | |
|  | Not concerned | | 0 | 176 | 187 | 201 | 564 | | | |  | |  | | | |
|  | Moderately concerned | | 1 | 302 | 231 | 168 | 701 | | | |  | |  | | | |
|  | Very concerned | | 2 | 170 | 104 | 56 | 330 | | | |  | |  | | | |
| Q22 | Adequate access to rapid tests | | All | 567 | 493 | 400 | 1460 | | | | 0.99 | | 0.88 | | | |
|  | All of the time | | 0 | 168 | 194 | 116 | 478 | | | |  | |  | | | |
|  | Most of the time | | 1 | 229 | 210 | 168 | 607 | | | |  | |  | | | |
|  | Some of the time | | 2 | 135 | 68 | 82 | 285 | | | |  | |  | | | |
|  | None of the time | | 3 | 35 | 21 | 34 | 90 | | | |  | |  | | | |
| Q23 | Adequate access to information | | All | 648 | 552 | 426 | 1596 | | | | 2.00 | | 0.78 | | | |
|  | All of the time | | 0 | 16 | 9 | 13 | 38 | | | |  | |  | | | |
|  | Most of the time | | 1 | 176 | 105 | 88 | 369 | | | |  | |  | | | |
|  | Some of the time | | 2 | 286 | 254 | 202 | 742 | | | |  | |  | | | |
|  | None of the time | | 3 | 170 | 154 | 123 | 447 | | | |  | |  | | | |
| Q24 | Excess information | | All | 643 | 518 | 424 | 1585 | | | | 1.32 | | 1.00 | | | |
|  | All of the time | | 3 | 131 | 100 | 51 | 282 | | | |  | |  | | | |
|  | Most of the time | | 2 | 124 | 91 | 69 | 284 | | | |  | |  | | | |
|  | Some of the time | | 1 | 263 | 228 | 192 | 683 | | | |  | |  | | | |
|  | None of the time | | 0 | 125 | 99 | 112 | 336 | | | |  | |  | | | |
| Q25 | Enough deployment training | | All | 575 | 457 | 378 | 1410 | | | | 1.92 | | 1.03 | | | |
|  | Definitely | | 0 | 60 | 49 | 42 | 151 | | | |  | |  | | | |
|  | Moderately | | 1 | 145 | 113 | 101 | 359 | | | |  | |  | | | |
|  | Slightly | | 2 | 144 | 109 | 95 | 348 | | | |  | |  | | | |
|  | Not at all | | 3 | 226 | 186 | 140 | 552 | | | |  | |  | | | |

Table A3. (Cont.) Catalogue of predictor variables and their numerical properties

|  |  | |  | Respondent numbers | | | | For z-score | | |
| --- | --- | --- | --- | --- | --- | --- | --- | --- | --- | --- |
|  | Predictor | Coding | | Survey 1 | Survey 2 | Survey 3 | Total | Mean | SD |  |
| Q26 | Currently provide quality care | | All | 601 | 490 | 403 | 1494 | 0.58 | 0.75 |  |
|  | Definitely | | 0 | 348 | 266 | 228 | 842 |  |  |  |
|  | Moderately | | 1 | 183 | 166 | 127 | 476 |  |  |  |
|  | Slightly | | 2 | 56 | 49 | 38 | 143 |  |  |  |
|  | Not at all | | 3 | 14 | 9 | 10 | 33 |  |  |  |
| Q27 | Supportive clinical team | | All | 642 | 517 | 422 | 1581 | 1.03 | 0.81 |  |
|  | All of the time | | 0 | 205 | 125 | 106 | 436 |  |  |  |
|  | Most of the time | | 1 | 282 | 246 | 182 | 710 |  |  |  |
|  | Some of the time | | 2 | 135 | 129 | 116 | 380 |  |  |  |
|  | None of the time | | 3 | 20 | 17 | 18 | 55 |  |  |  |

- - 1. **Justification of the use of standardized normal transformations of predictor variables in regression models:**

The relatively simple multivariate regression models available for analysis of this type of survey data permit the use of:

- - - 1. Either continuous interval data, rank-ordered data, or categorical data for the outcome (dependent) variables;
      2. Either continuous interval data, or categorical data, but not rank-ordered data, for the predictor (independent) variables.

There are no ideal statistical methods for analysis of the type of survey data produced by this study. Any analysis must represent a compromise between the different limitations of the different methodological choices. Better options could be produced, but as far as we are aware, they are not provided as validated methods in standard software packages.

Analysis of our survey data for violations of the assumptions of linear regression demonstrate that it would be more reliable to treat the outcome data as rank-ordered (See Section 2.1.1 and Table A2 above). The use of ordered logistic regression was chosen for this reason.

The predictor variables are clearly rank-ordered in nature (see Section 2.2.1 and Table A3 above). There are no simple multivariate regression models that allow this type of data to be used in a completely valid manner. We used a continuous presentation of the predictor variables to maintain a representation of the rank-ordering of the data. If the data were presented as categorical, the regression analysis would treat the data as nominal categories with no rank-ordering of values: this would lose most of the value of the data (i.e. the ranking of the data).

Calculation of standardized normal transformation (or z-score) using the values shown in the right-hand columns in Table A3 inserted in the equation:

$$=(Respondent value-Mean)/(Standard deviation)$$

This value has, by construction, a mean value of zero, and a standard deviation of one. Thus, in a regression model, the constant term (which is the mean effect size estimate for the population with or without adjustment) is estimated when all predictors are zero. If all the predictor variable are presented as standardized normal transformations, the regression constant is the adjusted population effect size mean, and the predictor variable coefficients are the effect of raising each predictor value by one standard deviation. This becomes a little abstract when binomial predictors such as gender are included, but the process does aid quick assimilation of the relative strength of the different predictors for the reader of the paper. If specific effects, such as gender comparisons, are required, these can be estimated with replacement of the gender z-score with the natural gender value, remembering that this will produce two estimates of the adjusted population mean value, one for females, one for males, if those values are of specific interest. Due to the large discrepancies in the numbers of women and men respondents, this was held not to be a major focus of the survey.

Table A4. Tabulation of missing data

|  | Question(s) | Complete | | Missing data / data imputed | | Missing % | Total |
| --- | --- | --- | --- | --- | --- | --- | --- |
| Outcome measures |  | |  | |  |  |  |
| GAD7 | Q36-42 | | 1577 | | 99 | 5.9% | 1676 |
| ISI7 | Q29-35 | | 1599 | | 77 | 4.6% | 1676 |
| IESR22 | Q47-68 | | 1505 | | 171 | 10.2% | 1676 |
| PHQ9 | Q69-Q77 | | 1278 | | 398 | 23.7% | 1676 |
| Time interaction variable | Qx time stamp | | 1676 | | 0 | 0.0% | 1676 |
| Predictor variables |  | |  | |  |  |  |
| Staff grade (Staff) | Q2 | | 1646 | | 30 | 1.8% | 1676 |
| Covid ward status (Covidward) | Q3 | | 1631 | | 45 | 2.7% | 1676 |
| Public sector (Public) | Q5 | | 1348 | | 328 | 19.6% | 1676 |
| Males (M) | Q6 | | 1641 | | 35 | 2.1% | 1676 |
| Education level (Educ) | Q7 | | 1642 | | 34 | 2.0% | 1676 |
| Age group (age) | Q8 | | 1645 | | 31 | 1.8% | 1676 |
| Social situation (Social) | Q9 | | 1405 | | 271 | 16.2% | 1676 |
| Smoking | Q10 | | 1619 | | 57 | 3.4% | 1676 |
| Current home/family stress | Q11 | | 1628 | | 48 | 2.9% | 1676 |
| Future home/family stress | Q12 | | 1540 | | 136 | 8.1% | 1676 |
| Current financial stress | Q13 | | 1618 | | 58 | 3.5% | 1676 |
| Future financial stress | Q14 | | 1477 | | 199 | 11.9% | 1676 |
| Intensity of exposure | Q15-20 | | 1624 | | 52 | 3.1% | 1676 |
| Concerned about PPE | Q21 | | 1595 | | 81 | 4.8% | 1676 |
| Poor access to rapid tests | Q22 | | 1460 | | 216 | 12.9% | 1676 |
| Inadequacy of information | Q23 | | 1596 | | 80 | 4.8% | 1676 |
| Excess of information | Q24 | | 1585 | | 91 | 5.4% | 1676 |
| Enough deployment training | Q25 | | 1410 | | 266 | 15.9% | 1676 |
| Poor ability for quality care | Q26 | | 1494 | | 182 | 10.9% | 1676 |
| Poor clinical team support | Q27 | | 1581 | | 95 | 5.7% | 1676 |

- - 1. **Missing data reporting and handling**

Missing data is tabulated in Table A4. This includes questions not answered, “Don’t know”, “Prefer not to answer” and “Not applicable” responses.

Without substitution, one of the missing data responses can be fitted into the analysis we have undertaken, based on placing the responses in an understandable rank order of meaning. Rather than let the respondent fall out of the analysis, which is the effect of providing no valid data for a particular question for particular respondents, the missing data was substituted by multiple imputation (MI). MI involves making a series of best guesses (20 guesses in this analysis) about what the responses might have been if the respondents had answered the question, based on all the other responses the individual respondent had given, using regression-type techniques. The multiple guesses are then integrated into each multiple regression model.

- - 1. **Primary regression analysis syntax:**

mi set wide

mi register imputed GAD7 insom7 IESR22 PHQ9 zStaff zPublic zM zCovidward zEduc1 zAge zSocial zSmoking zHFSN11 zHFSF12 zFnSN13 zFnSF14 zExpCv_Any zConPPE21 zAcTest22 zInadInfo23 zExInfo24 zDepTr25 zQCare26 zSTeam27

mi impute mvn GAD7 insom7 IESR22 PHQ9 zStaff zPublic zM zCovidward zEduc1 zAge zSocial zSmoking zHFSN11 zHFSF12 zFnSN13 zFnSF14 zExpCv_Any zConPPE21 zAcTest22 zInadInfo23 zExInfo24 zDepTr25 zQCare26 zSTeam27, add(20) rseed(2232) force

mi estimate: ologit GAD7 c.Timey_S1##c.zStaff c.Timey_S1##c.zEduc c.Timey_S1##c.zPublic c.Timey_S1##c.zM c.Timey_S1##c.zCovidward c.Timey_S1##c.zAge c.Timey_S1##c.zSocial c.Timey_S1##c.zSmoking c.Timey_S1##c. zHFSN11 c.Timey_S1##c. zHFSF12 c.Timey_S1##c.zFnSN13 c.Timey_S1##c.zFnSF14 c.Timey_S1##c.zExpCv_Any c.Timey_S1##c.zCoPPE21 c.Timey_S1##c.zAcTest22 c.Timey_S1##c. zInadInfo23 c.Timey_S1##c. zExInfo24 c.Timey_S1##c. zDepTr25 c.Timey_S1##c.zQCare26 c.Timey_S1##c.zSTeam27, vce(cl ID)

Table A5. Explanation of how the syntax terms operate in Stata 16.1 as used in this analysis

| Syntax term |  |
| --- | --- |
| mi impute mvn | Instruction: imputes missing values using the Stata multivariate normal regression method, an iterative Markov chain Monte Carlo (MCMC) method to impute missing values |
| mi estimate | Instruction: performs multiple imputation analysis on the standard statistical method |
| ologit | Standard statistical method: ordered logistic regression |
| GAD7 | Outcome measure of statistical model |
| Predictors: | All the next set of syntax terms define the predictor variables, and how they are interpreted by the software. There are 20 predictors, each with a time interaction term |
| c.*var* | Treats predictor variable as a continuous number; if no c. term is used, the software treats the predictor as a categorical variable. As a compromise between less ideal options, the predictor variables were analysed as continuous data. In principle, continuous predictor variables are used to estimate the linear association with the outcome variable; whilst categorical variables are used to estimate the mean differences between the different components of that variable (e.g. Gender would estimate the mean difference between males and females). |
| ## | This treats the two or more variables as interacting (multiple interactions can be specified) |
| Timey_S1 | This variable is the time the respondent completed the survey (in years), from a date zero set as the mean date of completion by the first survey respondents: all of the surveys are being included in this analysis (surveys 1, 2 and 3), and the three otherwise identical analyses using Timey_S1, Timey_S2 (zeroed at the mean date of survey 2), and Timey_S3 (zeroed at the mean date of survey 3).  This created three estimates of the association (odds ratios, ORs) between the outcome (GAD7) and the predictor variables at three time points corresponding to the mean ORs at those time points. The time-interaction created estimates of the linear change over time, the year of the 3 surveys: those ORs for change were identical when either Timey_S1, Timey_S2, or Timey_S3 were include in the model; the time variables were identical apart from different zero points. |

Table A5. (Cont.) Explanation of how the syntax terms operate in Stata 16.1 as used in this analysis

| Syntax term |  |
| --- | --- |
| Predictor names | The predictor variables used compacted names for convenience of processing the model output tables, corresponding to the predictors listed in table X. The “z” before each name indicates the use of the standardized normal transformation: our convention, not Stata’s. |
| vce(cl ID) | Indicates the type of variance-covariance matrix estimates used in the statistical model: in this case, it indicates clustering around ID (the respondent ID) in order to link identifiable individuals in the three surveys. The cluster syntax uses robust standard error estimation. |

- - 1. **Question 28 not included in the analysis**

Question 28, “Do you feel your workplace has supported you in the provision of food, accommodation and transportation as your workplace demands increase?” had a high level of missing data: 990 of 1676 (59%) failed to provide a usable answer. Most (871) of this missing data was a “Not applicable” response. We were not confident that this question could be analysed in an unbiased way.

All other predictor questions were included in the multivariate regression models used in the analysis, with missing data substituted using multiple imputation.

1. **Additional Results**
   1. **Association of workplace variables and psychological outcomes**

Guide to interpretation of the graphs (Section 3. Additional results):

- Each graph refers to a survey question, with the provided answers to those questions at each of the three Survey timepoints shown. The numbers of responders for each of the answers are shown as “Response numbers”
- The overall change in the psychological outcome over 1 year (e.g. GAD) is shown as an odds ratio (95%CI and P-value) corresponding to the top line “Change in GAD over 1 year” in each of Tables 3 to 6 in the main paper
- The changes in the strength of association, and the “Strength of association” at each Survey timepoint correspond to the line of results in Tables 3 to 6 in the main paper against each specific predictor
- The dash-lines in the graph correspond to the standard threshold values for the psychological outcome test scale, with the standard description of the ranges shown in the labels on the right
- The box-plots show the levels of percentiles of each group of responders (90^th^, 75^th^, 50^th^, 25^th^, 10^th^ from upper to lower, and outliers), grouped by answers and Survey timepoint
- All outcome values in the box-plots include missing data substituted by multiple imputation, but are otherwise not adjusted for any of the covariates. The odds ratios are adjusted for covariates.
- See our interpretation at the end of Section 3.
  - 1. **Q27. Do you feel adequately supported by your workplace team (colleagues and line manager)?**

| 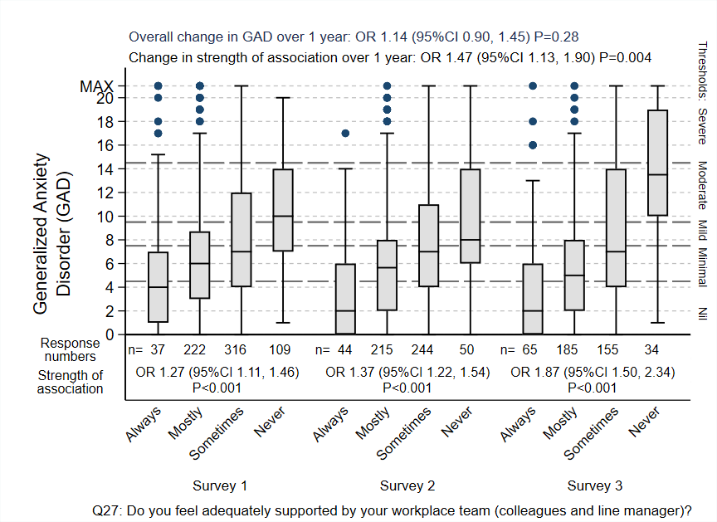 | 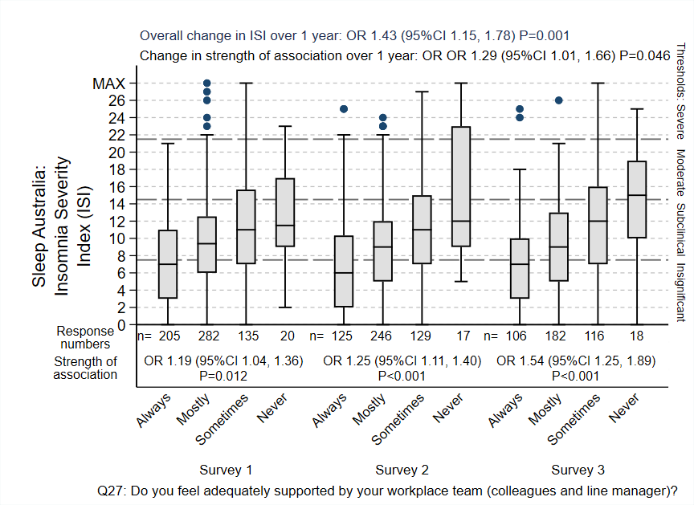 |
| --- | --- |
| 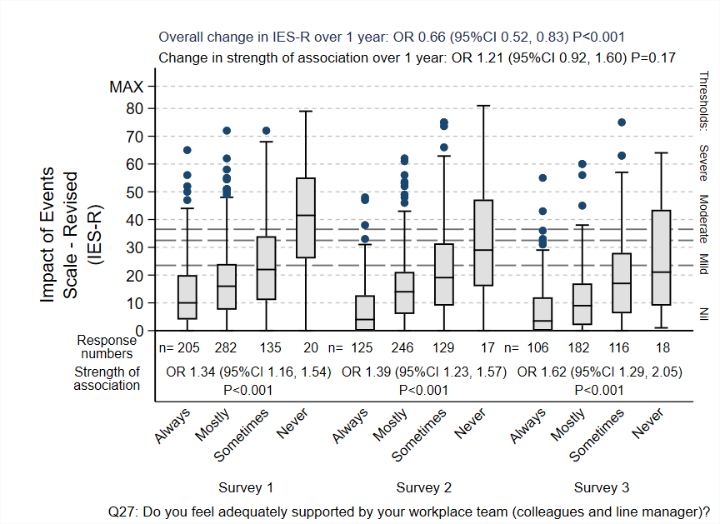 | 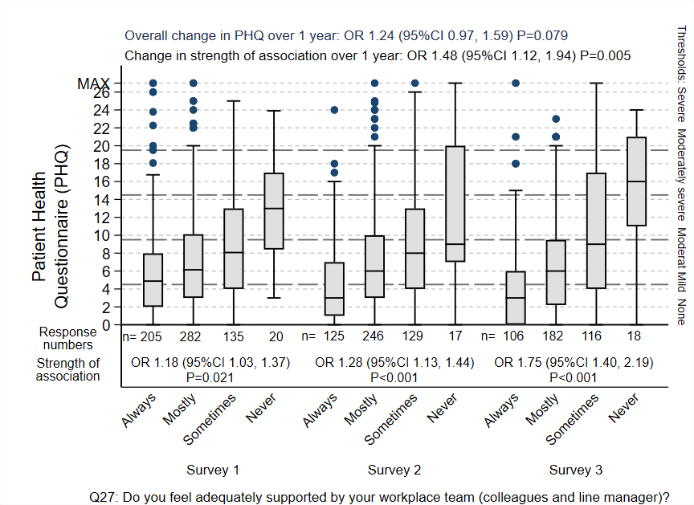 |

- - 1. **Q23. Do you feel you have inadequate access to up to date information, clear communications and guidelines?** Note that “adequate” has been replaced by “inadequate” reversing the order of the responses so that the graphs have a consistent appearance from good to bad stressors

| 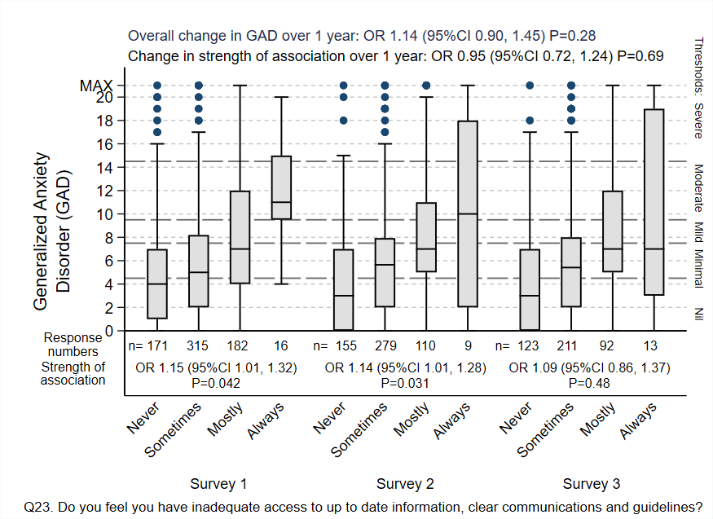 | 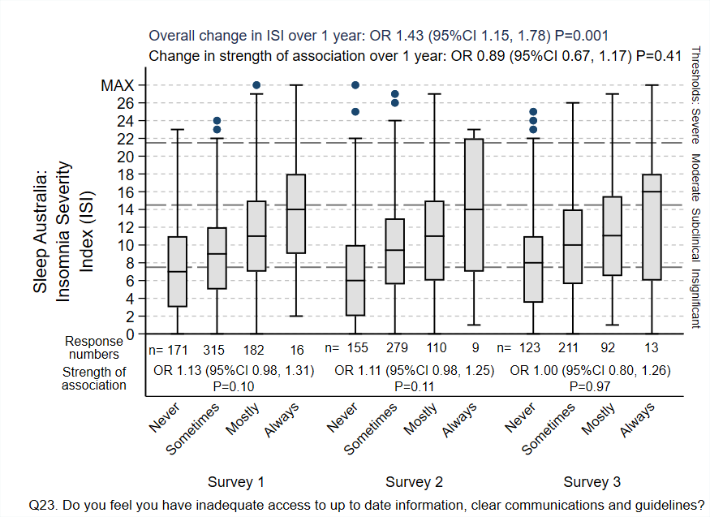 |
| --- | --- |
| 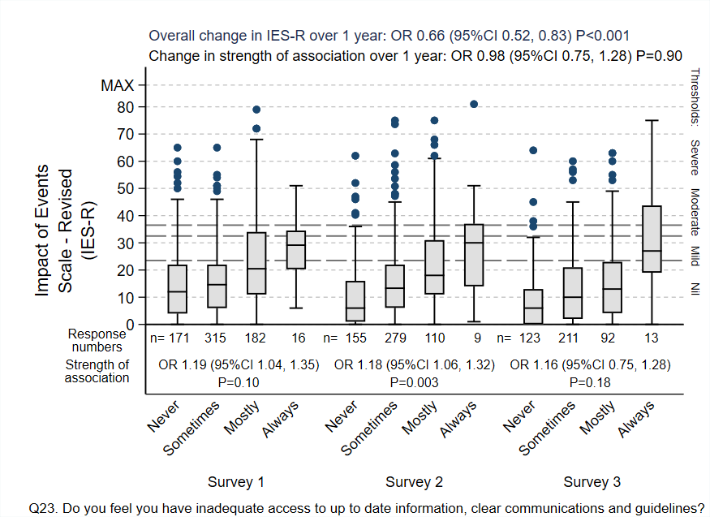 | 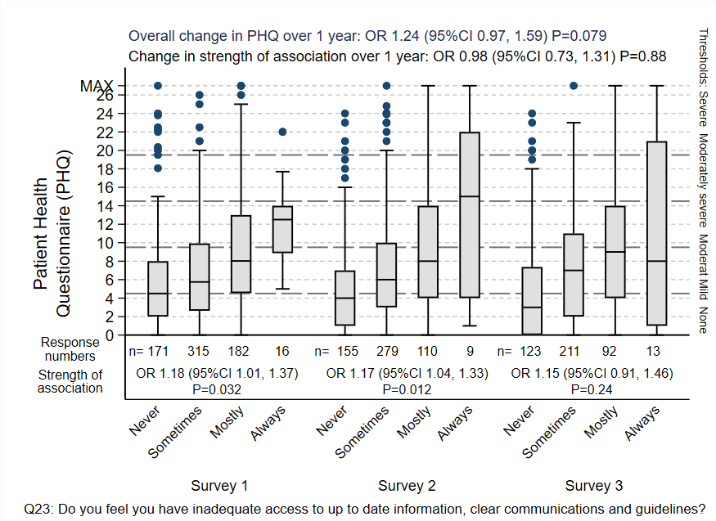 |

- - 1. **Q21. Are you concerned about adequate access to PPE?**

| 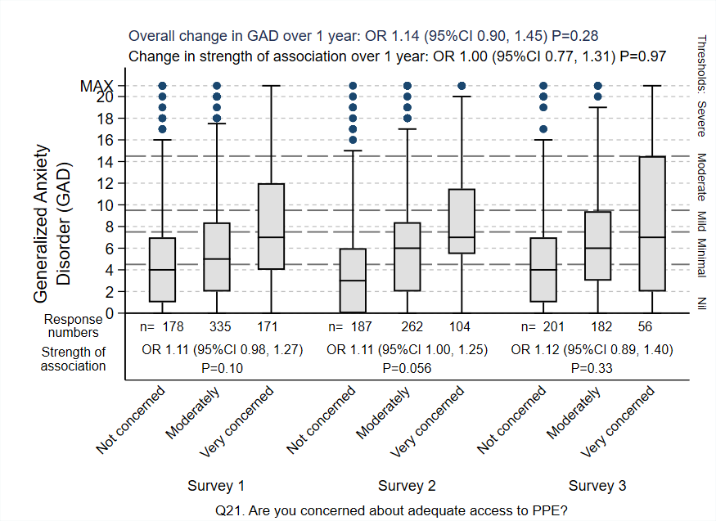 | 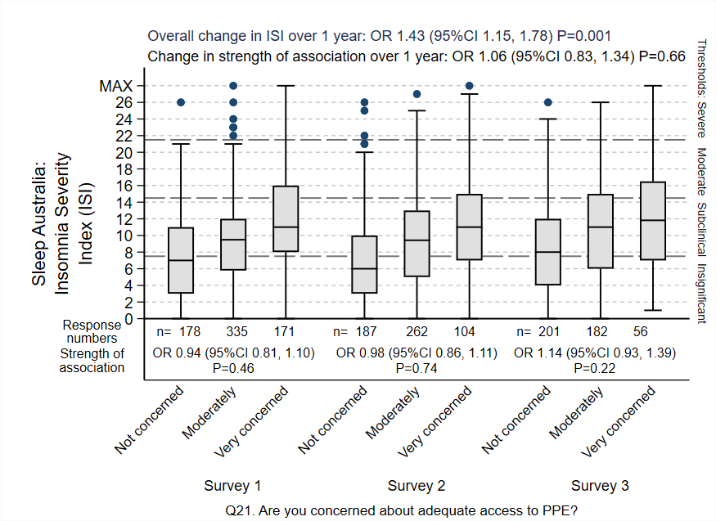 |
| --- | --- |
| 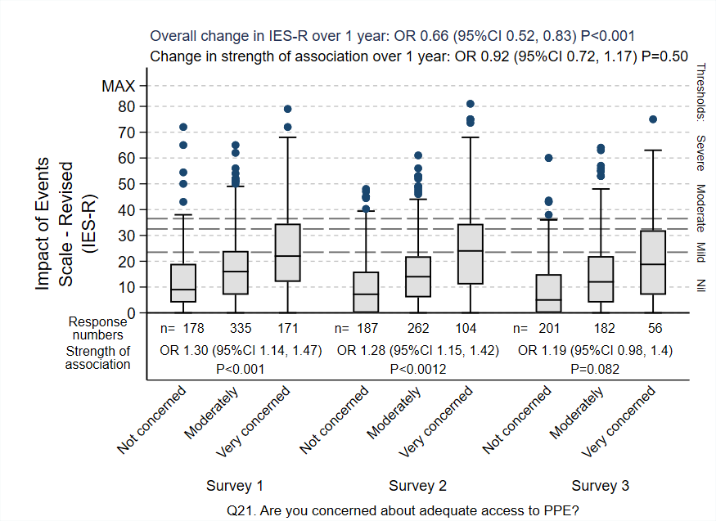 | 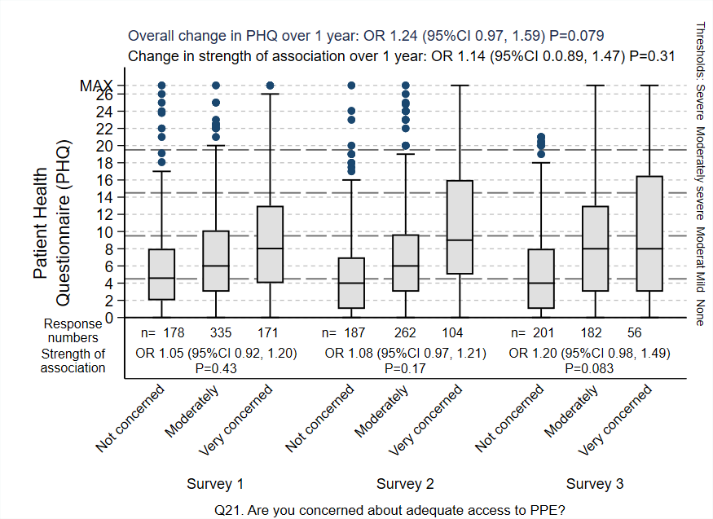 |

- - 1. **Q15 – Q20. Intensity of exposure:** see Table A3 for details of coding

| 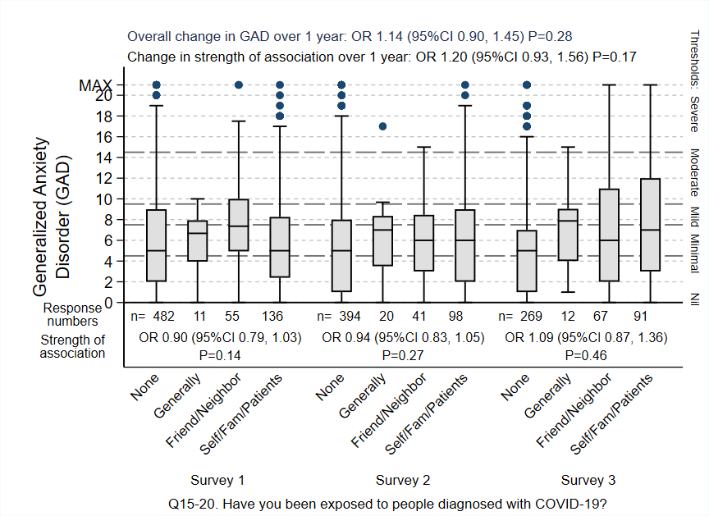 | 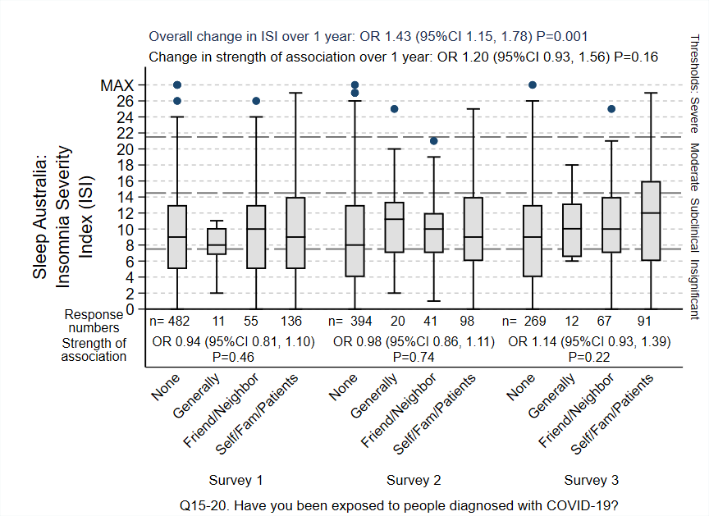 |
| --- | --- |
| 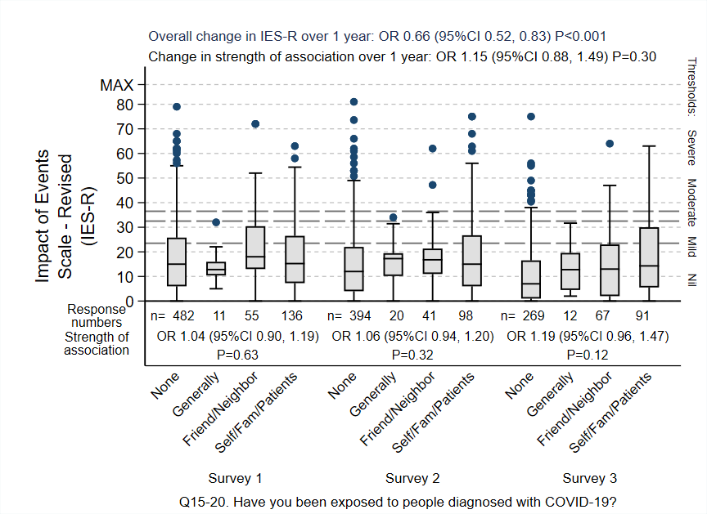 | 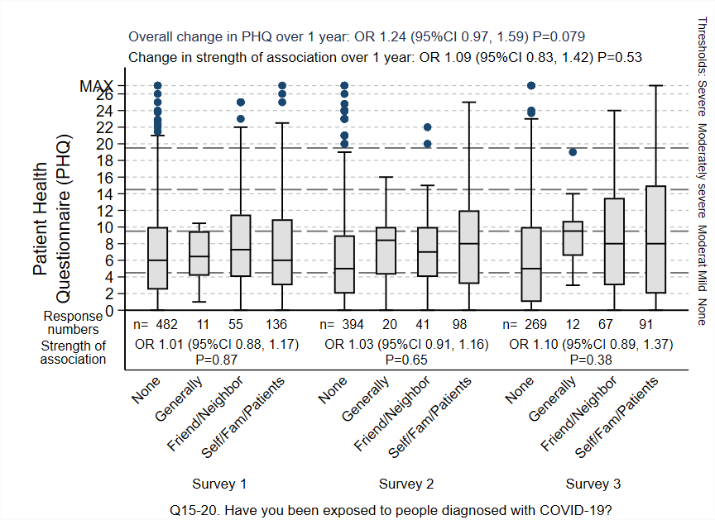 |

- 1. **Association of home-life variables and psychological outcomes**
     1. **Q11. To what degree has COVID-19 contributed to your CURRENT home and family stress?**

| 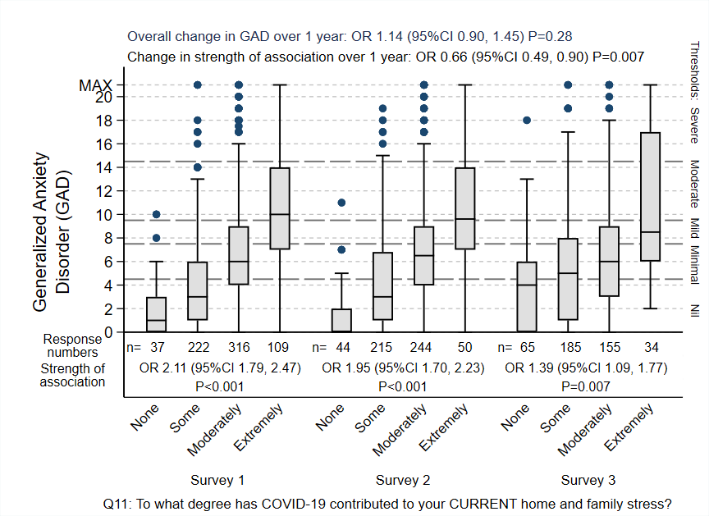 | 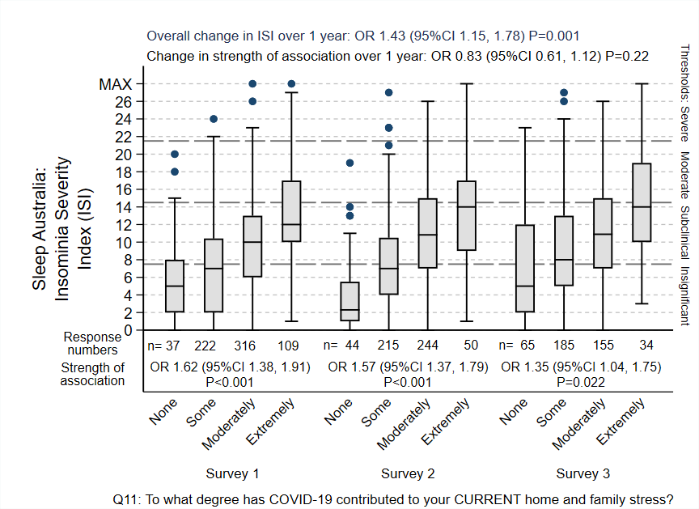 |
| --- | --- |
| 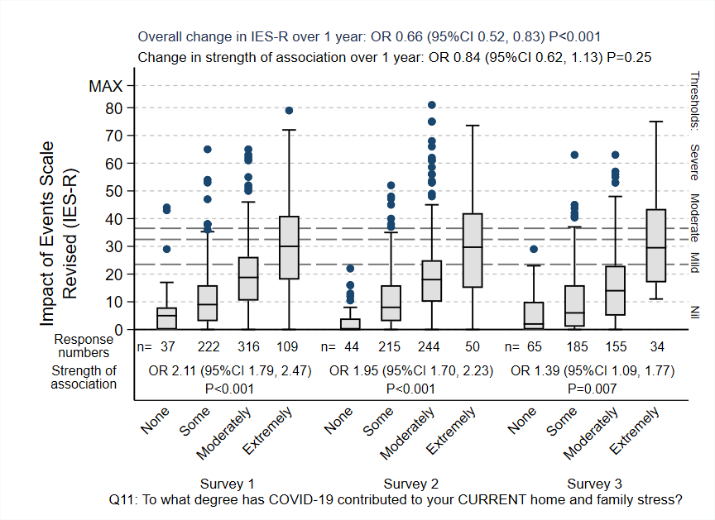 | 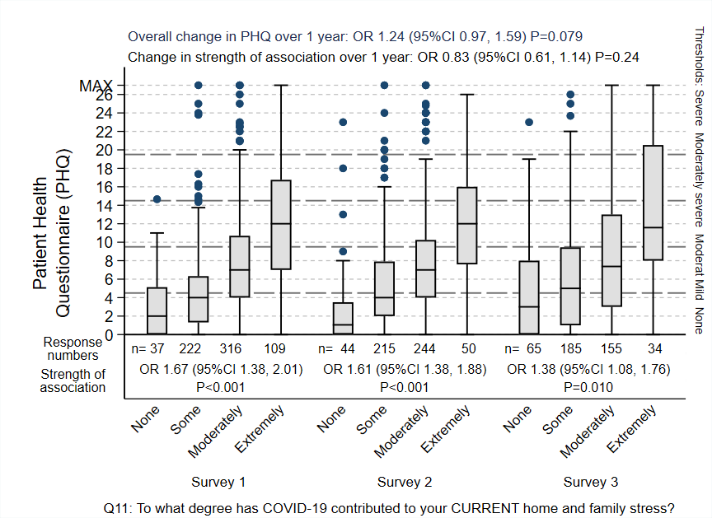 |

- - 1. **Q14. To what degree do you consider COVID-19 will contribute to your FUTURE financial stress?**

| 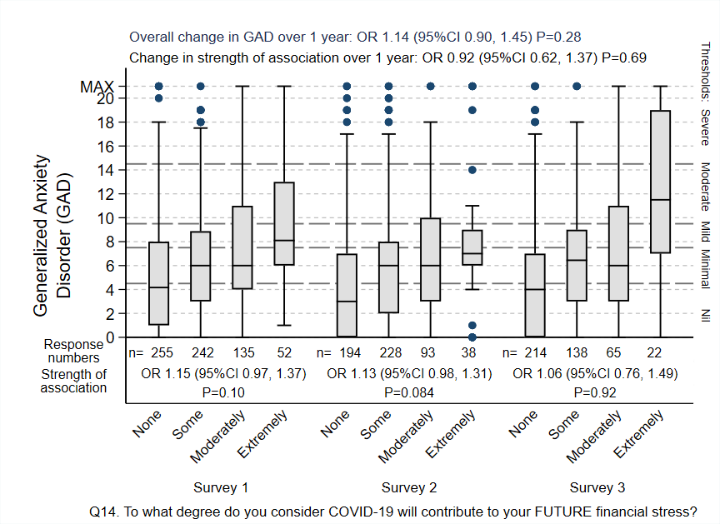 | 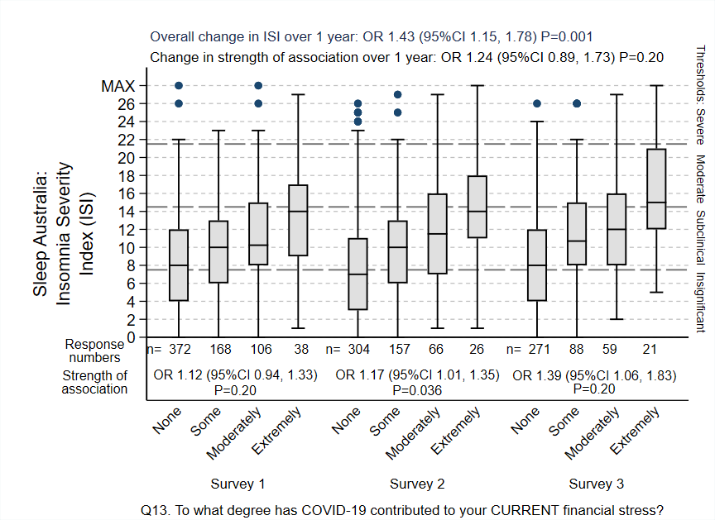 |
| --- | --- |
| 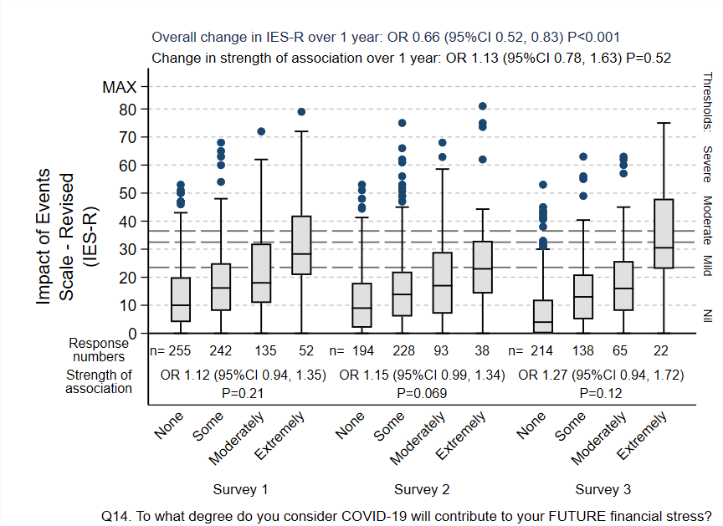 | 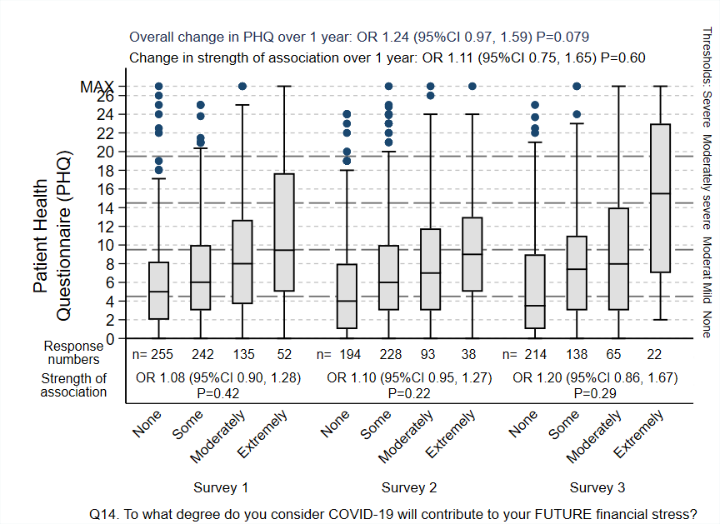 |

- 1. **Association of demographic variables and psychological outcomes**
     1. **Q7. Highest education level**

| 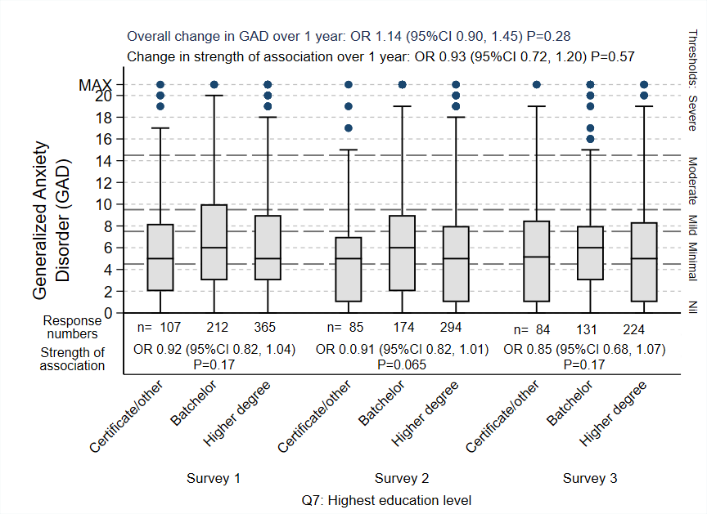 | 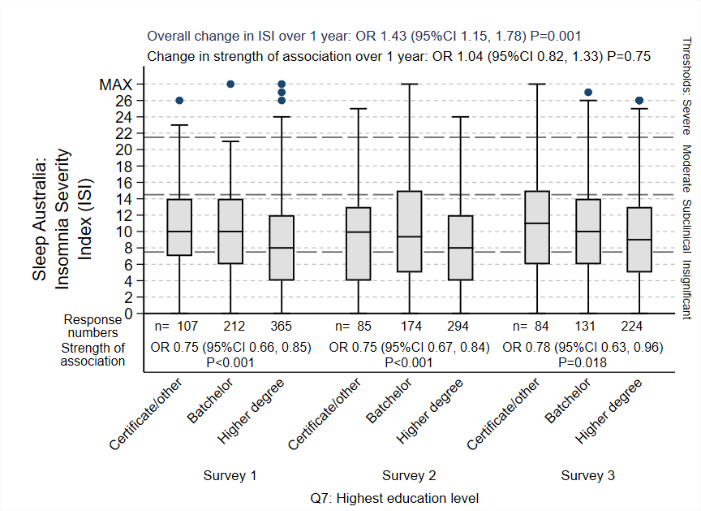 |
| --- | --- |
| 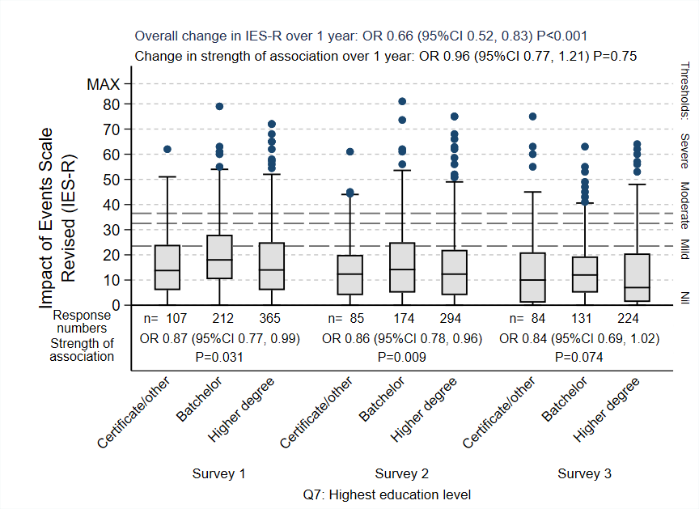 | 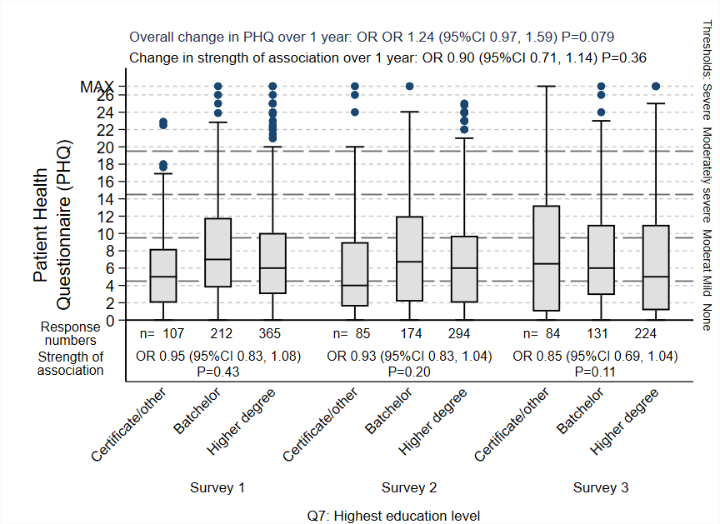 |
| - - 1. **Q8. What is your AGE GROUP?** |  |
| 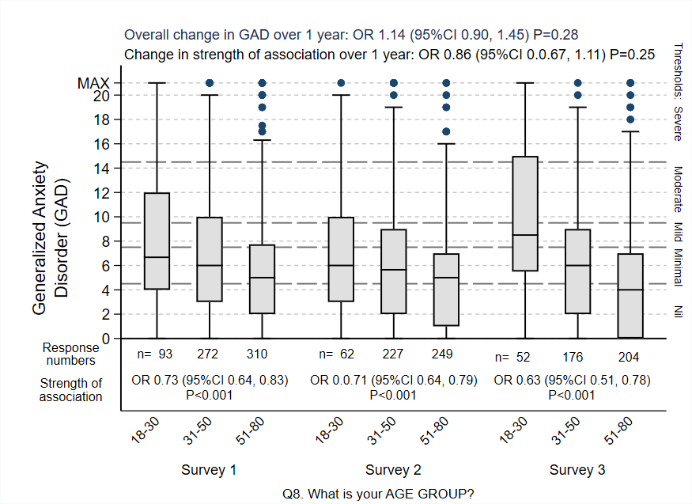 | 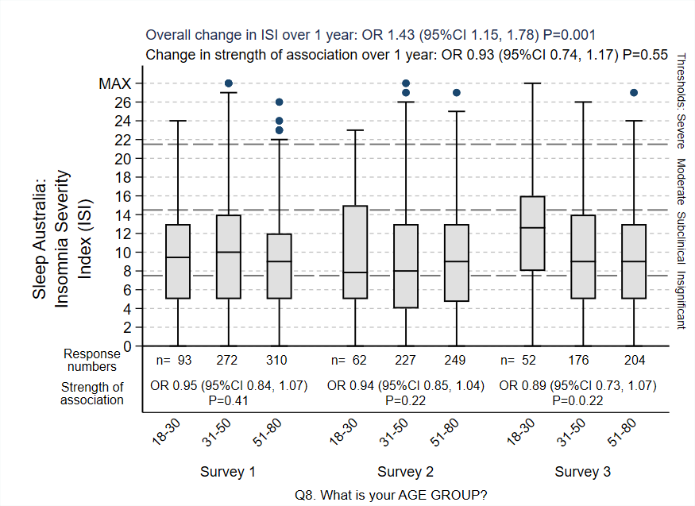 |
| 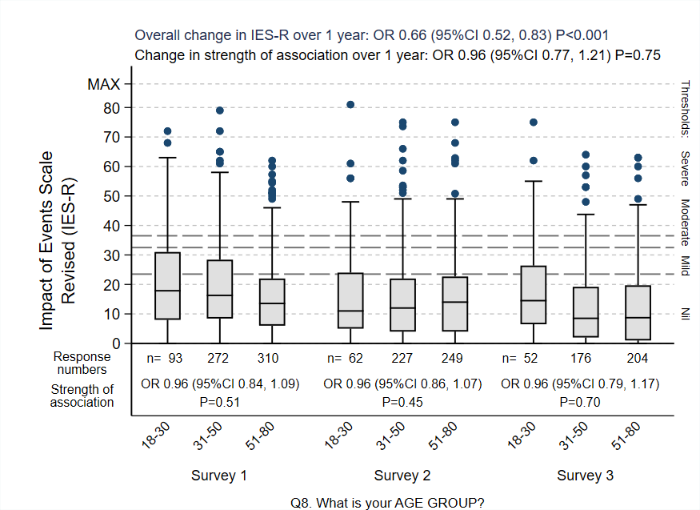 | 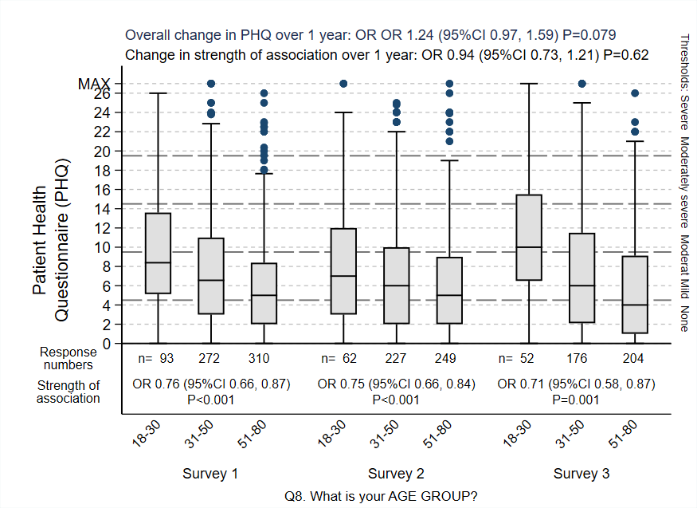 |

Commentary on these additional results and their interpretation:

Association of workplace variables and psychological outcomes

- The strongest associations seen are for poor workplace team support. There is a progressive rise in each outcome at each survey timepoint as support becomes poorer, and the variability of the outcomes (the height of the boxes) are relatively compact. There is a progressive rise in the strength of the association across the year (the ORs, and the position of the boxes becomes higher especially in the last survey). The Odds Ratios are adjusted for the other predictors, whilst the box=plot values are not.
- The other workplace predictors shown of Information Inadequacy and Inadequate PPE Access show similar patterns of association, but the increase in outcome values is somewhat less as levels of adverse predictors rise, and the Odds Ratios show weaker strengths of association. This may be explained by a confounding association between poor workplace support and the other workplace predictors: the qualitative responses seem to indicate that these two issues are seen as part of the workplace support issue, thus explaining the confounding effect.

Association of home-life variables and psychological outcomes

- In the initial survey, a strong association between current home/family stress and all outcomes was found, and this diminished somewhat over the year, although it remained a strong association.
- Current financial stress appears to show an association when viewed in isolation, but this becomes much weaker when adjusted for the other predictors. This may well be due to the financial stress being strongly associate with home/family stress, again from the qualitative responses. This confounding effect does not mean that financial issues are not important, just that they are operating through another question that is part of the survey.
- The intensity of exposure to COVID-19 does not show any strong association with psychological outcomes. This may have been because COVID exposure was not strong in Tasmania, or that the knowledge that COVID pandemic was occurring was having an effect, and that the individual level of exposure up to the time of the survey was not important: i.e. it was more of an existential issue rather than a concrete issue.

Association of demographic variables and psychological outcomes

- The main demographic issues of importance were education level, and age group. Over the short term, neither of these issues can be altered. However, the observations may suggest the need for tailoring any management responses to individual groups, based on these characteristics.
- There was a mild tendency for nurses with higher levels of education showed lower levels of insomnia than those with less formal education.
- Older nurses to show more anxiety and depression than the younger nurses.

Table A6. Psychological test median scores, and IQR (%) in each classification range, plus missing data case numbers

|  | Survey 1 |  |  |  | Survey 2 |  |  |  | Survey 3 |  |  |  |
| --- | --- | --- | --- | --- | --- | --- | --- | --- | --- | --- | --- | --- |
|  | Median n | IQR % | N | Missing | Median n | IQR % | N | Missing | Median n | IQR % | N | Missing |
| GAD Anxiety (GAD) | 5 | (2, 9) | 641 | 43 | 5 | (1, 8) | 514 | 39 | 5 | (1, 8) | 422 | 17 |
| Nil/minimal: 0-4 | 260 | 41% |  |  | 228 | 44% |  |  | 182 | 43% |  |  |
| Mild (Monitor): 5-7 | 167 | 26% |  |  | 148 | 29% |  |  | 115 | 37% |  |  |
| Mild (? Refer): 8-9 | 64 | 10% |  |  | 38 | 7% |  |  | 37 | 9% |  |  |
| Moderate (Refer): 10-14 | 94 | 15% |  |  | 59 | 11% |  |  | 38 | 9% |  |  |
| Severe (Treat): 15-21 | 56 | 9% |  |  | 41 | 8% |  |  | 50 | 12% |  |  |
| ISI Insomnia (ISI) | 9 | (5, 13) | 650 | 34 | 9 | (4, 13) | 522 | 31 | 9 | (5, 14) | 427 | 12 |
| Insignificant: 0-7 | 260 | 41% |  |  | 277 | 44% |  |  | 168 | 40% |  |  |
| Subclinical: 8-14 | 261 | 41% |  |  | 190 | 37% |  |  | 166 | 39% |  |  |
| Moderate: 15-21 | 115 | 18% |  |  | 85 | 17% |  |  | 74 | 18% |  |  |
| Severe: 22-28 | 14 | 2% |  |  | 20 | 4% |  |  | 19 | 5% |  |  |
| Stress, PTSD (IES-R) | 15 | (6, 26) | 617 | 67 | 12 | (4, 23) | 496 | 57 | 8 | (2, 21) | 396 | 43 |
| Insignificant: 0-23 | 423 | 69% |  |  | 371 | 75% |  |  | 322 | 81% |  |  |
| Concerning: 24-32 | 97 | 16% |  |  | 65 | 13% |  |  | 38 | 10% |  |  |
| Diagnostic: 33-36 | 20 | 3% |  |  | 10 | 2% |  |  | 6 | 2% |  |  |
| Immune suppression: 37-88 | 76 | 12% |  |  | 47 | 10% |  |  | 30 | 8% |  |  |
| Depression (PHQ) | 5 | (2, 9) | 406 | 278 | 5 | (2, 9) | 486 | 67 | 5 | (1, 10) | 386 | 53 |
| None: 0-4 | 201 | 50% |  |  | 237 | 49% |  |  | 189 | 49% |  |  |
| Mild: 5-9 | 120 | 30% |  |  | 143 | 29% |  |  | 94 | 24% |  |  |
| Moderate: 10-14 | 51 | 13% |  |  | 57 | 12% |  |  | 49 | 13% |  |  |
| Moderately severe: 15-19 | 20 | 5% |  |  | 28 | 6% |  |  | 35 | 9% |  |  |
| Severe: 20-27 | 14 | 3% |  |  | 21 | 4% |  |  | 19 | 5% |  |  |

1. **Sensitivity analyses.**
   1. **Effect of more or fewer variables on effect size estimates: are the different predictor variables truly independent? Justification for inclusion of all predictor variables in final regression model**

**First sensitivity analysis**: Concern has been raised by a reviewer about the choice of predictor variables for inclusion in the primary outcome regression models, although the nature and expected impact of the concern was not explained. A possible reason for this concern is that inclusion of too many predictor variables may lead to over-specification: there is excessive influence of individual case values on the effect size estimates. This is an issue in the analysis of smaller datasets, where low numbers of case per variable leads to individual case results distorting the estimates of association for between outcomes and individual predictors. Alternatively, where the objective of the study is to determine the minimum set of predictors required to specify a prediction of an outcome for the purpose of risk prediction in a future population, inclusion of a large number of predictor variable may force future practice to measure an inefficient excess of predictor variables.

We addressed the over-specification problem. The study analysed the effect of 21 predictor variables amongst 1,676 respondents: i.e. 80 cases per predictor variable, which would normally be considered an adequate number. Nevertheless, in order to satisfy this question, the numbers of predictor variables was reduced sequentially by removal of variables with least strength of association until a final reduced model of 11 predictor variables was chosen. The odds ratios for the full and limited models were then described in Tables A7 to A10. Inspection of the results demonstrate no important change in the effect size estimates in either absolute or relative size for particular predictors in the two models. This suggests that over-specification was a minimal problem in this analysis.

The purpose of the survey was to identify which of the possible reasons for differences in the psychological states of nursing staff appeared to have had the stronger or lesser relative impact on the measured outcomes. This information would then be used to focus the efforts of local nursing managers on relieving the potential psychological distresses associate with the Covid-19 pandemic, by allowing them to choose the factors that may seem to have the greatest impact and those most amenable to influence. The amenability would need to be judged separately. The analysis would thus be the everything-against-everything-else type of analysis as conducted by us, rather than an effort to create a risk measurement tool that includes only those predictors found to be most statistically “important” (as measured by post-estimation testing. A risk measurement tool may be appropriate for the purposes of selecting individual nurses-at-risk for individual therapy intended to reduce the levels of the psychological distress in those individuals. Future analysis may allow such a risk-prediction tool to be constructed, but that was not the intention of this particular paper, and it would not be regarded by us as the priority for the first productive results from the survey. Efforts to improve the lives of all the nurses impacted by the Covid-19 pandemic were judged to be of most immediate value.

Specific postestimation tests were mentioned by a reviewer, which I understand to be relevant to the selection of the optimum regression model: i.e. Akaike's and Schwarz's Bayesian information criteria, as well as details relevant to mixed effects regression models. These cannot be provided, since the advice given by the reviewer to use multiple imputation to account for missing data (with which we agree) prevents such postestimation testing: Stata 16.1 does not support such testing when multiple imputation is being used. The details of models that were used are shown in Additional Materials Section 2.2.6.

Table A7. Associations with Generalized Anxiety Disorder (GAD) in full (1) and limited (2) models:

|  |  | Survey 1 (Initial; N=684) | | | Survey 2 (3-month; N=553) | | | Survey 3 (12-month; N=439) | | | Change in GAD over 1 year ^3,4^ | | |
| --- | --- | --- | --- | --- | --- | --- | --- | --- | --- | --- | --- | --- | --- |
| Predictors | Model | OR | 95%CI | P-value | OR | 95%CI | P-value | OR | 95%CI | P-value | OR | 95%CI | P-value |
| Change in GAD over 1 year ^3^ | 1 |  |  |  |  |  |  |  |  |  | 1.14 | (0.90, 1.45) | 0.28 |
|  | 2 |  |  |  |  |  |  |  |  |  | 1.12 | (0.89, 1.41) | 0.33 |
| Current home/family stress | 1 | 2.13 | (1.81, 2.50) | <0.001 | 1.98 | (1.73, 2.26) | <0.001 | 1.44 | (1.12, 1.85) | 0.005 | 0.68 | (0.50, 0.92) | 0.013 |
|  | 2 | 2.14 | (1.83, 2.49) | <0.001 | 1.96 | (1.73, 2.23) | <0.001 | 1.36 | (1.07, 1.74) | 0.013 | 0.64 | (0.48, 0.86) | 0.003 |
| Poor clinical team support | 1 | 1.27 | (1.11, 1.46) | <0.001 | 1.37 | (1.22, 1.54) | <0.001 | 1.87 | (1.50, 2.34) | <0.001 | 1.47 | (1.13, 1.90) | 0.004 |
|  | 2 | 1.29 | (1.14, 1.47) | <0.001 | 1.39 | (1.24, 1.55) | <0.001 | 1.87 | (1.52, 2.29) | <0.001 | 1.44 | (1.13, 1.84) | 0.003 |
| Future home/family stress | 1 | 1.19 | (1.02, 1.39) | 0.032 | 1.15 | (1.01, 1.31) | 0.040 | 0.99 | (0.76, 1.28) | 0.93 | 0.83 | (0.60, 1.14) | 0.26 |
|  | 2 | 1.16 | (0.99, 1.37) | 0.063 | 1.13 | (0.98, 1.29) | 0.083 | 0.98 | (0.74, 1.30) | 0.91 | 0.85 | (0.61, 1.18) | 0.33 |
| Future financial stress | 1 | 1.15 | (0.97, 1.37) | 0.10 | 1.13 | (0.98, 1.31) | 0.084 | 1.06 | (0.76, 1.49) | 0.72 | 0.92 | (0.62, 1.37) | 0.69 |
|  | 2 | 1.15 | (0.97, 1.36) | 0.12 | 1.12 | (0.97, 1.30) | 0.14 | 1.01 | (0.72, 1.43) | 0.95 | 0.88 | (0.59, 1.31) | 0.54 |
| Inadequacy of information | 1 | 1.15 | (1.01, 1.32) | 0.042 | 1.14 | (1.01, 1.28) | 0.031 | 1.09 | (0.86, 1.37) | 0.48 | 0.95 | (0.72, 1.24) | 0.69 |
|  | 2 | 1.14 | (1.00, 1.30) | 0.043 | 1.15 | (1.03, 1.29) | 0.012 | 1.19 | (0.95, 1.50) | 0.13 | 1.05 | (0.80, 1.36) | 0.74 |
| Poor ability for quality care | 1 | 1.09 | (0.96, 1.25) | 0.19 | 1.10 | (0.98, 1.24) | 0.12 | 1.13 | (0.88, 1.44) | 0.33 | 1.03 | (0.78, 1.36) | 0.83 |
|  | 2 | 1.15 | (1.01, 1.31) | 0.040 | 1.15 | (1.02, 1.29) | 0.019 | 1.14 | (0.93, 1.41) | 0.21 | 1.00 | (0.78, 1.28) | 0.98 |
| Covid ward status | 1 | 1.09 | (0.97, 1.24) | 0.16 | 1.06 | (0.95, 1.18) | 0.28 | 0.93 | (0.74, 1.17) | 0.55 | 0.85 | (0.66, 1.11) | 0.23 |
|  | 2 | 1.10 | (0.98, 1.24) | 0.11 | 1.06 | (0.95, 1.17) | 0.29 | 0.89 | (0.72, 1.10) | 0.29 | 0.81 | (0.64, 1.04) | 0.095 |
| Excessive information | 1 | 1.07 | (0.95, 1.21) | 0.27 | 1.06 | (0.96, 1.18) | 0.26 | 1.03 | (0.84, 1.25) | 0.81 | 0.96 | (0.76, 1.21) | 0.71 |
| Concerned about PPE | 1 | 1.11 | (0.98, 1.27) | 0.10 | 1.11 | (1.00, 1.25) | 0.056 | 1.12 | (0.89, 1.40) | 0.33 | 1.00 | (0.77, 1.31) | 0.97 |
|  | 2 | 1.12 | (0.98, 1.27) | 0.095 | 1.11 | (1.00, 1.24) | 0.060 | 1.10 | (0.88, 1.36) | 0.41 | 0.98 | (0.76, 1.27) | 0.89 |
| Smoking | 1 | 1.05 | (0.94, 1.18) | 0.36 | 1.07 | (0.97, 1.17) | 0.19 | 1.12 | (0.96, 1.31) | 0.15 | 1.06 | (0.87, 1.29) | 0.55 |
| Enough deployment training | 1 | 1.04 | (0.91, 1.18) | 0.57 | 1.04 | (0.93, 1.16) | 0.51 | 1.04 | (0.84, 1.28) | 0.71 | 1.00 | (0.78, 1.28) | 0.99 |
| Poor access to RATs | 1 | 1.05 | (0.93, 1.18) | 0.45 | 1.06 | (0.96, 1.17) | 0.25 | 1.12 | (0.91, 1.38) | 0.27 | 1.07 | (0.84, 1.37) | 0.59 |
| Public sector | 1 | 1.04 | (0.92, 1.18) | 0.51 | 1.04 | (0.94, 1.15) | 0.44 | 1.04 | (0.82, 1.33) | 0.74 | 1.00 | (0.75, 1.33) | 1.00 |
| Social situation | 1 | 1.05 | (0.94, 1.18) | 0.41 | 1.03 | (0.94, 1.14) | 0.50 | 0.97 | (0.79, 1.20) | 0.78 | 0.93 | (0.72, 1.18) | 0.54 |
| Lower staff grade | 1 | 0.98 | (0.86, 1.10) | 0.68 | 0.97 | (0.88, 1.08) | 0.60 | 0.96 | (0.76, 1.22) | 0.74 | 0.99 | (0.75, 1.30) | 0.92 |
| Current financial stress | 1 | 0.98 | (0.82, 1.17) | 0.83 | 1.03 | (0.89, 1.19) | 0.70 | 1.26 | (0.91, 1.75) | 0.17 | 1.29 | (0.87, 1.89) | 0.21 |
|  | 2 | 1.03 | (0.86, 1.22) | 0.77 | 1.08 | (0.92, 1.25) | 0.34 | 1.32 | (0.96, 1.81) | 0.086 | 1.28 | (0.89, 1.86) | 0.18 |
| Education level | 1 | 0.92 | (0.82, 1.04) | 0.17 | 0.91 | (0.82, 1.01) | 0.065 | 0.85 | (0.68, 1.07) | 0.17 | 0.93 | (0.72, 1.20) | 0.57 |
| Intensity of Covid exposure | 1 | 0.90 | (0.79, 1.03) | 0.14 | 0.94 | (0.83, 1.05) | 0.27 | 1.09 | (0.87, 1.36) | 0.46 | 1.20 | (0.93, 1.56) | 0.17 |
|  | 2 | 0.87 | (0.76, 0.98) | 0.023 | 0.93 | (0.84, 1.03) | 0.18 | 1.26 | (1.03, 1.55) | 0.026 | 1.45 | (1.14, 1.85) | 0.002 |
| Age group | 1 | 0.73 | (0.64, 0.83) | <0.001 | 0.71 | (0.64, 0.79) | <0.001 | 0.63 | (0.51, 0.78) | <0.001 | 0.86 | (0.67, 1.11) | 0.25 |
|  | 2 | 0.75 | (0.66, 0.85) | <0.001 | 0.72 | (0.64, 0.81) | <0.001 | 0.63 | (0.50, 0.79) | <0.001 | 0.84 | (0.65, 1.09) | 0.19 |
| Males | 1 | 0.83 | (0.74, 0.94) | 0.002 | 0.84 | (0.76, 0.93) | 0.001 | 0.86 | (0.69, 1.08) | 0.20 | 1.03 | (0.80, 1.33) | 0.79 |

Table A8. Associations with Sleep Australia: Insomnia Severity Index (ISI) in full (1) and limited (2) models:

|  |  | Survey 1 | | | Survey 2 | | | Survey 3 | | | Change in ISI over 1 year ^3^ | | |
| --- | --- | --- | --- | --- | --- | --- | --- | --- | --- | --- | --- | --- | --- |
| Predictors | Model | OR | 95%CI | P-value | OR | 95%CI | P-value | OR | 95%CI | P-value | OR | 95%CI | P-value |
| Change in ISI over 1 year ^3^ | 1 |  |  |  |  |  |  |  |  |  | 1.43 | (1.15, 1.78) | 0.001 |
|  | 2 |  |  |  |  |  |  |  |  |  | 1.37 | (1.10, 1.71) | 0.004 |
| Current home/family stress | 1 | 1.64 | (1.39, 1.94) | <0.001 | 1.58 | (1.38, 1.82) | <0.001 | 1.35 | (1.06, 1.73) | 0.015 | 0.83 | (0.61, 1.12) | 0.22 |
|  | 2 | 1.62 | (1.38, 1.91) | <0.001 | 1.55 | (1.35, 1.77) | <0.001 | 1.27 | (0.98, 1.65) | 0.072 | 0.79 | (0.57, 1.08) | 0.14 |
| Future home/family stress | 1 | 1.29 | (1.10, 1.52) | 0.002 | 1.21 | (1.06, 1.39) | 0.005 | 0.93 | (0.73, 1.18) | 0.55 | 0.72 | (0.53, 0.97) | 0.034 |
|  | 2 | 1.27 | (1.09, 1.48) | 0.002 | 1.20 | (1.05, 1.36) | 0.006 | 0.94 | (0.74, 1.19) | 0.59 | 0.74 | (0.55, 0.99) | 0.043 |
| Poor clinical team support | 1 | 1.19 | (1.04, 1.36) | 0.012 | 1.25 | (1.11, 1.40) | <0.001 | 1.54 | (1.25, 1.89) | <0.001 | 1.29 | (1.01, 1.66) | 0.046 |
|  | 2 | 1.20 | (1.05, 1.37) | 0.006 | 1.25 | (1.12, 1.40) | <0.001 | 1.50 | (1.22, 1.83) | <0.001 | 1.24 | (0.98, 1.59) | 0.078 |
| Poor access to rapid tests | 1 | 1.16 | (1.02, 1.32) | 0.026 | 1.16 | (1.04, 1.30) | 0.007 | 1.18 | (0.97, 1.44) | 0.089 | 1.02 | (0.80, 1.30) | 0.87 |
|  | 2 | 1.15 | (1.01, 1.31) | 0.035 | 1.14 | (1.02, 1.28) | 0.019 | 1.12 | (0.90, 1.38) | 0.31 | 0.97 | (0.76, 1.25) | 0.81 |
| Concerned about PPE | 1 | 1.12 | (0.98, 1.27) | 0.10 | 1.13 | (1.01, 1.26) | 0.034 | 1.18 | (0.97, 1.43) | 0.10 | 1.06 | (0.83, 1.34) | 0.66 |
|  | 2 | 1.13 | (0.99, 1.30) | 0.062 | 1.15 | (1.03, 1.28) | 0.016 | 1.20 | (0.99, 1.45) | 0.057 | 1.06 | (0.84, 1.34) | 0.63 |
| Current financial stress | 1 | 1.12 | (0.94, 1.33) | 0.20 | 1.17 | (1.01, 1.35) | 0.036 | 1.39 | (1.06, 1.83) | 0.017 | 1.24 | (0.89, 1.73) | 0.20 |
|  | 2 | 1.17 | (1.03, 1.33) | 0.017 | 1.23 | (1.10, 1.38) | 0.000 | 1.54 | (1.28, 1.87) | <0.001 | 1.32 | (1.05, 1.66) | 0.019 |
| Covid ward status | 1 | 1.10 | (0.96, 1.26) | 0.19 | 1.07 | (0.94, 1.21) | 0.30 | 0.95 | (0.75, 1.20) | 0.66 | 0.86 | (0.66, 1.14) | 0.30 |
|  | 2 | 1.11 | (0.98, 1.25) | 0.11 | 1.07 | (0.96, 1.19) | 0.25 | 0.91 | (0.72, 1.16) | 0.45 | 0.83 | (0.64, 1.08) | 0.16 |
| Poor ability for quality care | 1 | 1.09 | (0.96, 1.24) | 0.20 | 1.08 | (0.96, 1.21) | 0.19 | 1.03 | (0.80, 1.33) | 0.81 | 0.95 | (0.70, 1.27) | 0.72 |
|  | 2 | 1.13 | (0.99, 1.28) | 0.071 | 1.13 | (1.01, 1.26) | 0.030 | 1.15 | (0.92, 1.43) | 0.22 | 1.02 | (0.79, 1.32) | 0.88 |
| Inadequacy of information | 1 | 1.13 | (0.98, 1.31) | 0.10 | 1.11 | (0.98, 1.25) | 0.11 | 1.00 | (0.80, 1.26) | 0.97 | 0.89 | (0.67, 1.17) | 0.41 |
|  | 2 | 1.14 | (0.99, 1.32) | 0.064 | 1.13 | (1.00, 1.28) | 0.044 | 1.09 | (0.86, 1.39) | 0.46 | 0.96 | (0.73, 1.26) | 0.75 |
| Enough deployment training | 1 | 1.08 | (0.94, 1.24) | 0.29 | 1.08 | (0.96, 1.21) | 0.21 | 1.09 | (0.89, 1.33) | 0.43 | 1.01 | (0.79, 1.29) | 0.94 |
| Future financial stress | 1 | 1.03 | (0.87, 1.23) | 0.72 | 1.05 | (0.90, 1.21) | 0.54 | 1.11 | (0.85, 1.46) | 0.44 | 1.08 | (0.77, 1.51) | 0.66 |
| Excessive information | 1 | 1.00 | (0.89, 1.13) | 0.97 | 1.03 | (0.93, 1.13) | 0.62 | 1.13 | (0.92, 1.38) | 0.25 | 1.12 | (0.88, 1.43) | 0.34 |
| Public sector | 1 | 1.04 | (0.91, 1.17) | 0.58 | 1.07 | (0.96, 1.19) | 0.22 | 1.22 | (0.99, 1.49) | 0.058 | 1.17 | (0.92, 1.50) | 0.20 |
| Social situation | 1 | 1.04 | (0.92, 1.18) | 0.55 | 1.04 | (0.93, 1.15) | 0.50 | 1.03 | (0.87, 1.21) | 0.75 | 0.99 | (0.80, 1.21) | 0.91 |
| Lower staff grade | 1 | 0.99 | (0.88, 1.11) | 0.84 | 0.97 | (0.88, 1.07) | 0.58 | 0.91 | (0.76, 1.09) | 0.32 | 0.92 | (0.74, 1.15) | 0.48 |
| Males | 1 | 0.98 | (0.87, 1.10) | 0.75 | 0.95 | (0.86, 1.05) | 0.33 | 0.83 | (0.67, 1.02) | 0.083 | 0.84 | (0.66, 1.07) | 0.17 |
| Age group | 1 | 0.95 | (0.84, 1.07) | 0.41 | 0.94 | (0.85, 1.04) | 0.22 | 0.89 | (0.73, 1.07) | 0.22 | 0.93 | (0.74, 1.17) | 0.55 |
| Smoking | 1 | 0.96 | (0.84, 1.10) | 0.59 | 0.99 | (0.89, 1.11) | 0.90 | 1.12 | (0.96, 1.31) | 0.14 | 1.16 | (0.96, 1.42) | 0.13 |
| Intensity of Covid exposure | 1 | 0.94 | (0.81, 1.10) | 0.46 | 0.98 | (0.86, 1.11) | 0.74 | 1.14 | (0.93, 1.39) | 0.22 | 1.20 | (0.93, 1.56) | 0.16 |
|  | 2 | 0.91 | (0.80, 1.04) | 0.18 | 0.96 | (0.86, 1.07) | 0.45 | 1.17 | (0.99, 1.40) | 0.070 | 1.28 | (1.03, 1.61) | 0.029 |
| Higher education level | 1 | 0.75 | (0.66, 0.85) | <0.001 | 0.75 | (0.67, 0.84) | <0.001 | 0.78 | (0.63, 0.96) | 0.018 | 1.04 | (0.82, 1.33) | 0.75 |
|  | 2 | 0.77 | (0.68, 0.87) | <0.001 | 0.78 | (0.70, 0.86) | <0.001 | 0.82 | (0.69, 0.97) | 0.022 | 1.06 | (0.87, 1.30) | 0.57 |

Table A9. Associations with Impact of Events Scale - Revised (IES-R) in full (1) and limited (2) models:

|  |  | Survey 1 | | | Survey 2 | | | Survey 3 | | | Change in IES-R over 1 year ^3^ | | |
| --- | --- | --- | --- | --- | --- | --- | --- | --- | --- | --- | --- | --- | --- |
| Predictors | Model | OR | 95%CI | P-value | OR | 95%CI | P-value | OR | 95%CI | P-value | OR | 95%CI | P-value |
| Change in IES-R over 1 year ^3^ | 1 |  |  |  |  |  |  |  |  |  | 0.66 | (0.52, 0.83) | <0.001 |
|  | 2 |  |  |  |  |  |  |  |  |  | 0.63 | (0.50, 0.79) | <0.001 |
| Current home/family stress | 1 | 1.95 | (1.64, 2.33) | <0.001 | 1.89 | (1.63, 2.18) | <0.001 | 1.64 | (1.30, 2.06) | <0.001 | 0.84 | (0.62, 1.13) | 0.25 |
|  | 2 | 1.99 | (1.67, 2.36) | <0.001 | 1.90 | (1.65, 2.19) | <0.001 | 1.56 | (1.23, 1.99) | <0.001 | 0.79 | (0.58, 1.07) | 0.13 |
| Poor clinical team support | 1 | 1.34 | (1.16, 1.54) | <0.001 | 1.39 | (1.23, 1.57) | <0.001 | 1.62 | (1.29, 2.05) | <0.001 | 1.21 | (0.92, 1.60) | 0.17 |
|  | 2 | 1.30 | (1.13, 1.49) | <0.001 | 1.35 | (1.20, 1.53) | <0.001 | 1.62 | (1.31, 2.00) | <0.001 | 1.25 | (0.98, 1.60) | 0.077 |
| Concerned about PPE | 1 | 1.30 | (1.14, 1.47) | <0.001 | 1.28 | (1.15, 1.42) | <0.001 | 1.19 | (0.98, 1.46) | 0.082 | 0.92 | (0.72, 1.17) | 0.50 |
|  | 2 | 1.28 | (1.13, 1.45) | <0.001 | 1.27 | (1.14, 1.41) | <0.001 | 1.22 | (1.00, 1.49) | 0.045 | 0.96 | (0.75, 1.22) | 0.72 |
| Poor ability for quality care | 1 | 1.15 | (1.01, 1.32) | 0.039 | 1.12 | (0.99, 1.25) | 0.065 | 0.97 | (0.75, 1.25) | 0.80 | 0.84 | (0.62, 1.13) | 0.25 |
|  | 2 | 1.23 | (1.07, 1.40) | 0.003 | 1.21 | (1.08, 1.36) | 0.001 | 1.13 | (0.91, 1.40) | 0.28 | 0.92 | (0.71, 1.19) | 0.51 |
| Future home/family stress | 1 | 1.26 | (1.06, 1.49) | 0.009 | 1.22 | (1.07, 1.41) | 0.004 | 1.09 | (0.84, 1.42) | 0.50 | 0.87 | (0.62, 1.22) | 0.42 |
|  | 2 | 1.19 | (1.00, 1.40) | 0.046 | 1.18 | (1.02, 1.35) | 0.022 | 1.13 | (0.86, 1.49) | 0.39 | 0.95 | (0.68, 1.34) | 0.78 |
| Future financial stress | 1 | 1.12 | (0.94, 1.35) | 0.21 | 1.15 | (0.99, 1.34) | 0.069 | 1.27 | (0.94, 1.72) | 0.12 | 1.13 | (0.78, 1.63) | 0.52 |
|  | 2 | 1.14 | (0.96, 1.36) | 0.13 | 1.14 | (0.98, 1.32) | 0.080 | 1.13 | (0.82, 1.56) | 0.44 | 0.99 | (0.68, 1.44) | 0.96 |
| Inadequacy of information | 1 | 1.19 | (1.04, 1.35) | 0.010 | 1.18 | (1.06, 1.32) | 0.003 | 1.16 | (0.93, 1.46) | 0.18 | 0.98 | (0.75, 1.28) | 0.90 |
|  | 2 | 1.19 | (1.05, 1.35) | 0.007 | 1.19 | (1.07, 1.32) | 0.001 | 1.19 | (0.98, 1.45) | 0.083 | 1.00 | (0.79, 1.27) | 0.99 |
| Enough deployment training | 1 | 1.02 | (0.90, 1.17) | 0.71 | 1.06 | (0.95, 1.18) | 0.33 | 1.21 | (0.98, 1.48) | 0.071 | 1.17 | (0.93, 1.49) | 0.18 |
| Excessive information | 1 | 1.10 | (0.97, 1.25) | 0.13 | 1.10 | (0.99, 1.22) | 0.077 | 1.10 | (0.91, 1.31) | 0.33 | 0.99 | (0.80, 1.24) | 0.96 |
| Covid ward status | 1 | 1.08 | (0.95, 1.23) | 0.26 | 1.06 | (0.95, 1.19) | 0.29 | 1.01 | (0.79, 1.29) | 0.91 | 0.94 | (0.72, 1.24) | 0.67 |
| Social situation | 1 | 1.12 | (0.99, 1.27) | 0.073 | 1.10 | (0.99, 1.22) | 0.089 | 0.99 | (0.83, 1.19) | 0.95 | 0.89 | (0.71, 1.11) | 0.29 |
|  | 2 | 1.12 | (0.97, 1.29) | 0.12 | 1.10 | (0.97, 1.24) | 0.12 | 1.02 | (0.83, 1.24) | 0.88 | 0.91 | (0.71, 1.17) | 0.45 |
| Current financial stress | 1 | 1.10 | (0.92, 1.32) | 0.27 | 1.17 | (1.00, 1.36) | 0.050 | 1.47 | (1.08, 1.99) | 0.014 | 1.33 | (0.93, 1.90) | 0.12 |
|  | 2 | 1.10 | (0.92, 1.31) | 0.30 | 1.16 | (1.00, 1.35) | 0.055 | 1.46 | (1.08, 1.97) | 0.013 | 1.33 | (0.93, 1.88) | 0.11 |
| Smoking | 1 | 1.00 | (0.90, 1.13) | 0.94 | 1.04 | (0.94, 1.14) | 0.45 | 1.19 | (0.97, 1.47) | 0.098 | 1.19 | (0.93, 1.52) | 0.18 |
| Public sector | 1 | 1.04 | (0.90, 1.21) | 0.59 | 1.04 | (0.92, 1.18) | 0.50 | 1.05 | (0.89, 1.25) | 0.54 | 1.01 | (0.81, 1.27) | 0.91 |
| Intensity of Covid exposure | 1 | 1.04 | (0.90, 1.19) | 0.63 | 1.06 | (0.94, 1.20) | 0.32 | 1.19 | (0.96, 1.47) | 0.12 | 1.15 | (0.88, 1.49) | 0.30 |
|  | 2 | 1.01 | (0.90, 1.13) | 0.88 | 1.01 | (0.90, 1.13) | 0.88 | 1.01 | (0.90, 1.13) | 0.88 | 1.16 | (0.91, 1.48) | 0.22 |
| Age group | 1 | 0.96 | (0.84, 1.09) | 0.51 | 0.96 | (0.86, 1.07) | 0.45 | 0.96 | (0.79, 1.17) | 0.70 | 1.00 | (0.79, 1.28) | 0.97 |
| Poor access to rapid tests | 1 | 0.96 | (0.84, 1.09) | 0.51 | 0.97 | (0.87, 1.08) | 0.55 | 1.01 | (0.81, 1.25) | 0.94 | 1.05 | (0.81, 1.36) | 0.70 |
| Higher education level | 1 | 0.87 | (0.77, 0.99) | 0.031 | 0.86 | (0.78, 0.96) | 0.009 | 0.84 | (0.69, 1.02) | 0.074 | 0.96 | (0.77, 1.21) | 0.75 |
|  | 2 | 0.90 | (0.82, 0.99) | 0.039 | 0.90 | (0.82, 0.99) | 0.039 | 0.90 | (0.82, 0.99) | 0.039 | 0.96 | (0.77, 1.20) | 0.73 |
| Lower staff grade | 1 | 0.90 | (0.79, 1.02) | 0.090 | 0.90 | (0.81, 0.99) | 0.039 | 0.89 | (0.74, 1.06) | 0.20 | 0.99 | (0.79, 1.24) | 0.91 |
| Males | 1 | 0.79 | (0.70, 0.88) | <0.001 | 0.81 | (0.73, 0.89) | <0.001 | 0.90 | (0.72, 1.13) | 0.36 | 1.14 | (0.89, 1.48) | 0.30 |

Table A10. Associations with Patient Health Questionnaire (PHQ) in full (1) and limited (2) models:

|  |  | Survey 1 | | | Survey 2 | | | Survey 3 | | | Change in PHQ over 1 year ^3^ | | |
| --- | --- | --- | --- | --- | --- | --- | --- | --- | --- | --- | --- | --- | --- |
| Predictors | Model | OR | 95%CI | P-value | OR | 95%CI | P-value | OR | 95%CI | P-value | OR | 95%CI | P-value |
| Change in PHQ over 1 year ^3^ | 1 |  |  |  |  |  |  |  |  |  | 1.24 | (0.97, 1.59) | 0.079 |
|  | 2 |  |  |  |  |  |  |  |  |  | 1.16 | (0.92, 1.46) | 0.20 |
| Current home/family stress | 1 | 1.67 | (1.38, 2.01) | <0.001 | 1.61 | (1.38, 1.88) | <0.001 | 1.38 | (1.08, 1.76) | 0.010 | 0.83 | (0.61, 1.14) | 0.24 |
|  | 2 | 1.74 | (1.47, 2.06) | <0.001 | 1.67 | (1.46, 1.92) | <0.001 | 1.40 | (1.11, 1.75) | 0.004 | 0.80 | (0.59, 1.09) | 0.16 |
| Future home/family stress | 1 | 1.26 | (1.05, 1.50) | 0.014 | 1.20 | (1.03, 1.40) | 0.017 | 1.00 | (0.77, 1.30) | 1.00 | 0.80 | (0.58, 1.10) | 0.17 |
|  | 2 | 1.26 | (1.07, 1.48) | 0.006 | 1.22 | (1.07, 1.39) | 0.003 | 1.07 | (0.84, 1.37) | 0.58 | 0.85 | (0.62, 1.17) | 0.32 |
| Poor ability for quality care | 1 | 1.17 | (1.03, 1.34) | 0.019 | 1.15 | (1.02, 1.29) | 0.022 | 1.05 | (0.82, 1.35) | 0.71 | 0.90 | (0.68, 1.19) | 0.44 |
|  | 2 | 1.24 | (1.08, 1.43) | 0.002 | 1.22 | (1.08, 1.37) | 0.001 | 1.12 | (0.90, 1.40) | 0.31 | 0.90 | (0.69, 1.19) | 0.47 |
| Poor clinical team support | 1 | 1.18 | (1.03, 1.35) | 0.015 | 1.27 | (1.13, 1.42) | <0.001 | 1.72 | (1.40, 2.12) | <0.001 | 1.46 | (1.13, 1.88) | 0.004 |
|  | 2 | 1.20 | (1.06, 1.37) | 0.004 | 1.30 | (1.16, 1.44) | <0.001 | 1.77 | (1.45, 2.17) | <0.001 | 1.47 | (1.15, 1.87) | 0.002 |
| Social situation | 1 | 1.20 | (1.05, 1.38) | 0.006 | 1.20 | (1.07, 1.34) | 0.002 | 1.17 | (0.96, 1.43) | 0.13 | 0.97 | (0.76, 1.24) | 0.82 |
|  | 2 | 1.19 | (1.03, 1.36) | 0.016 | 1.15 | (1.03, 1.30) | 0.017 | 1.03 | (0.83, 1.28) | 0.81 | 0.87 | (0.67, 1.13) | 0.29 |
| Inadequacy of information | 1 | 1.18 | (1.01, 1.37) | 0.032 | 1.17 | (1.04, 1.33) | 0.012 | 1.15 | (0.91, 1.46) | 0.24 | 0.98 | (0.73, 1.31) | 0.88 |
|  | 2 | 1.17 | (1.02, 1.35) | 0.030 | 1.18 | (1.05, 1.33) | 0.007 | 1.22 | (0.98, 1.52) | 0.083 | 1.04 | (0.79, 1.36) | 0.79 |
| Enough deployment training | 1 | 1.10 | (0.94, 1.28) | 0.22 | 1.11 | (0.97, 1.26) | 0.12 | 1.15 | (0.95, 1.39) | 0.16 | 1.04 | (0.82, 1.33) | 0.74 |
| Excessive information | 1 | 1.07 | (0.93, 1.22) | 0.35 | 1.07 | (0.95, 1.19) | 0.26 | 1.07 | (0.89, 1.30) | 0.46 | 1.01 | (0.80, 1.27) | 0.95 |
| Future financial stress | 1 | 1.08 | (0.90, 1.28) | 0.42 | 1.10 | (0.95, 1.27) | 0.22 | 1.20 | (0.86, 1.67) | 0.29 | 1.11 | (0.75, 1.65) | 0.60 |
| Smoking | 1 | 1.05 | (0.94, 1.18) | 0.39 | 1.07 | (0.97, 1.18) | 0.20 | 1.13 | (0.93, 1.37) | 0.22 | 1.07 | (0.85, 1.35) | 0.55 |
| Covid ward status | 1 | 1.04 | (0.91, 1.19) | 0.55 | 1.02 | (0.91, 1.15) | 0.71 | 0.95 | (0.74, 1.21) | 0.65 | 0.91 | (0.69, 1.20) | 0.49 |
| Concerned about PPE | 1 | 1.05 | (0.92, 1.20) | 0.43 | 1.08 | (0.97, 1.21) | 0.17 | 1.20 | (0.98, 1.49) | 0.083 | 1.14 | (0.89, 1.47) | 0.31 |
|  | 2 | 1.06 | (0.92, 1.23) | 0.39 | 1.06 | (0.92, 1.23) | 0.39 | 1.21 | (0.98, 1.48) | 0.077 | 1.13 | (0.88, 1.46) | 0.34 |
| Current financial stress | 1 | 1.08 | (0.90, 1.29) | 0.42 | 1.08 | (0.93, 1.25) | 0.32 | 1.09 | (0.80, 1.48) | 0.58 | 1.01 | (0.70, 1.47) | 0.94 |
| Higher education level | 1 | 0.95 | (0.83, 1.08) | 0.43 | 0.93 | (0.83, 1.04) | 0.20 | 0.85 | (0.69, 1.04) | 0.11 | 0.90 | (0.71, 1.14) | 0.36 |
| Public sector | 1 | 1.04 | (0.90, 1.21) | 0.60 | 1.02 | (0.90, 1.15) | 0.78 | 0.93 | (0.74, 1.16) | 0.52 | 0.89 | (0.68, 1.17) | 0.41 |
| Poor access to rapid tests | 1 | 0.98 | (0.86, 1.11) | 0.71 | 0.99 | (0.89, 1.10) | 0.85 | 1.05 | (0.85, 1.29) | 0.67 | 1.07 | (0.83, 1.39) | 0.59 |
| Lower staff grade | 1 | 0.95 | (0.84, 1.08) | 0.42 | 0.96 | (0.87, 1.07) | 0.47 | 1.02 | (0.84, 1.24) | 0.85 | 1.07 | (0.84, 1.38) | 0.57 |
| Intensity of exposure | 1 | 1.01 | (0.88, 1.17) | 0.87 | 1.03 | (0.91, 1.16) | 0.65 | 1.10 | (0.89, 1.37) | 0.38 | 1.09 | (0.83, 1.42) | 0.53 |
|  | 2 | 0.94 | (0.83, 1.07) | 0.34 | 0.99 | (0.89, 1.10) | 0.84 | 1.22 | (0.99, 1.50) | 0.063 | 1.29 | (1.00, 1.66) | 0.047 |
| Age group | 1 | 0.81 | (0.70, 0.93) | 0.003 | 0.79 | (0.70, 0.89) | <0.001 | 0.71 | (0.57, 0.88) | 0.002 | 0.87 | (0.67, 1.14) | 0.32 |
|  | 2 | 0.80 | (0.69, 0.92) | 0.001 | 0.78 | (0.69, 0.88) | <0.001 | 0.71 | (0.57, 0.88) | 0.002 | 0.89 | (0.68, 1.16) | 0.38 |
| Males | 1 | 0.68 | (0.47, 0.98) | 0.039 | 0.66 | (0.48, 0.91) | 0.011 | 0.60 | (0.32, 1.09) | 0.094 | 0.87 | (0.43, 1.79) | 0.71 |

Footnote to Tables A7 to A10

These analyses examine the question that appears to have been raised by a reviewer: Does the inclusion or exclusion of different predictor variables alter the estimates of the associations between the individual variables and the outcome scores? The same ordered logistic regression analyses were performed on the 17 selected predictor variables, and also on predictor variables with potentially significant associations (i.e. potential confounding variables), selected by removal of variables with multivariate P-values <0.2 in the full model by regression in the multiple imputation analysis.

1. Associations between predictors and outcomes, and change in outcomes over 1 year were estimated using repeated-measures ordered logistic regression; the effects were shown as odds ratios (OR; 95% confidence intervals; P-values), adjusted for each of the predictors shown in the tables; missing data was substituted by multiple imputation. Odds ratios of 1.00 indicate no association; OR more than 1.00 indicate a positive association; OR less than 1.00 indicate a negative association. The odds ratio values shown are the effect of a rise of 1 standard deviation of each of the predictor variables.
2. Two models are presented: 1) the full model including all the predictor variables that appeared independent (light green shading; 2) more limited model with
3. The association between predictors and outcomes at the start of the pandemic (1^st^ April 2020) was fixed by the zero value of the time interaction predictor.
4. The change in association between each predictor and outcomes was determined by the time interaction, with the time defined as the number of years from 1^st^ April 2020 to the date each respondent completed each of the surveys. Mean date of survey completion was: Survey 1, 0.1 years 6/05/2020; Survey 2, 0.29 years 6/05/2020; Survey 3, 1.1 years 8/05/2021.
   1. **Does the inability to accurately identify individuals who responded to more than one survey affect the predictor effect size estimates?**

**Second sensitivity analysis**: When individuals contribute more than one observation to a study, this may violate the assumption of independence of observations made by the statistical models. This can lead to a loss of precision in the estimate of the effect sizes, and also an underestimation of the variability of those estimates. This is normally dealt with by repeated-measures / cluster correction in the regression analyses. This correction relies on being able to identify which observations are repeated observations in an individual. This identification may have been inaccurate in our analyses.

There were apparently 139 survey responses where an individual completed more than one survey at a particular survey time-point. We chose to exclude 139 survey responses for these apparent individuals by randomly selecting one of the multiple responses for inclusion and excluding the rest. This situation, however, may have arisen by different individuals choosing the same identification code, resulting in incorrect exclusion of some or all of those 139 apparent duplicates. A sensitivity analysis was conducted in order to test whether the magnitude and variability of the effect size estimates was changed by this decision. The results are shown in Tables

Table A11. Associations with Generalized Anxiety Disorder (GAD) in full models: with either exclusion of apparent duplicate responses (Group 1), or inclusion of all available responses (Group2)

|  |  | Survey 1 | | | Survey 2 | | | Survey 3 | | | Change in GAD over 1 year ^3^ | | |
| --- | --- | --- | --- | --- | --- | --- | --- | --- | --- | --- | --- | --- | --- |
|  | Group^2^ | OR^1^ | 95%CI | P-value | OR^1^ | 95%CI | P-value | OR^1^ | 95%CI | P-value | OR^1^ | 95%CI | P-value |
| Change in GAD over 1 year^3^ | 1 |  |  |  |  |  |  |  |  |  | 1.14 | (0.90, 1.45) | 0.28 |
|  | 2 |  |  |  |  |  |  |  |  |  | 1.13 | (0.89, 1.44) | 0.30 |
| Predictors |  |  |  |  |  |  |  |  |  |  |  |  |  |
| Current home/family stress | 1 | 2.13 | (1.81, 2.50) | <0.001 | 1.98 | (1.73, 2.26) | <0.001 | 1.44 | (1.12, 1.85) | 0.005 | 0.68 | (0.50, 0.92) | 0.013 |
|  | 2 | 2.13 | (1.81, 2.51) | <0.001 | 1.97 | (1.72, 2.27) | <0.001 | 1.41 | (1.10, 1.82) | 0.007 | 0.66 | (0.49, 0.90) | 0.009 |
| Poor clinical team support | 1 | 1.27 | (1.11, 1.46) | <0.001 | 1.37 | (1.22, 1.54) | <0.001 | 1.87 | (1.50, 2.34) | <0.001 | 1.47 | (1.13, 1.90) | 0.004 |
|  | 2 | 1.28 | (1.11, 1.47) | 0.001 | 1.37 | (1.21, 1.54) | <0.001 | 1.83 | (1.47, 2.27) | <0.001 | 1.43 | (1.11, 1.84) | 0.01 |
| Future home/family stress | 1 | 1.19 | (1.02, 1.39) | 0.032 | 1.15 | (1.01, 1.31) | 0.040 | 0.99 | (0.76, 1.28) | 0.93 | 0.83 | (0.60, 1.14) | 0.26 |
|  | 2 | 1.17 | (1.00, 1.38) | 0.056 | 1.13 | (0.98, 1.30) | 0.085 | 0.96 | (0.73, 1.25) | 0.74 | 0.82 | (0.59, 1.13) | 0.22 |
| Future financial stress | 1 | 1.15 | (0.97, 1.37) | 0.10 | 1.13 | (0.98, 1.31) | 0.084 | 1.06 | (0.76, 1.49) | 0.72 | 0.92 | (0.62, 1.37) | 0.69 |
|  | 2 | 1.16 | (0.98, 1.38) | 0.087 | 1.15 | (0.99, 1.33) | 0.061 | 1.09 | (0.78, 1.53) | 0.61 | 0.94 | (0.63, 1.40) | 0.77 |
| Inadequacy of information | 1 | 1.15 | (1.01, 1.32) | 0.042 | 1.14 | (1.01, 1.28) | 0.031 | 1.09 | (0.86, 1.37) | 0.48 | 0.95 | (0.72, 1.24) | 0.69 |
|  | 2 | 1.16 | (1.01, 1.33) | 0.036 | 1.15 | (1.02, 1.29) | 0.025 | 1.10 | (0.88, 1.38) | 0.40 | 0.95 | (0.73, 1.24) | 0.71 |
| Concerned about PPE | 1 | 1.11 | (0.98, 1.27) | 0.10 | 1.11 | (1.00, 1.25) | 0.056 | 1.12 | (0.89, 1.40) | 0.33 | 1.00 | (0.77, 1.31) | 0.97 |
|  | 2 | 1.10 | (0.96, 1.26) | 0.16 | 1.10 | (0.98, 1.24) | 0.095 | 1.11 | (0.89, 1.38) | 0.35 | 1.01 | (0.78, 1.30) | 0.96 |
| Poor ability for quality care | 1 | 1.09 | (0.96, 1.25) | 0.19 | 1.10 | (0.98, 1.24) | 0.12 | 1.13 | (0.88, 1.44) | 0.33 | 1.03 | (0.78, 1.36) | 0.83 |
|  | 2 | 1.13 | (0.98, 1.31) | 0.084 | 1.14 | (1.01, 1.30) | 0.036 | 1.19 | (0.93, 1.51) | 0.17 | 1.04 | (0.79, 1.38) | 0.76 |
| Covid ward status | 1 | 1.09 | (0.97, 1.24) | 0.16 | 1.06 | (0.95, 1.18) | 0.28 | 0.93 | (0.74, 1.17) | 0.55 | 0.85 | (0.66, 1.11) | 0.23 |
|  | 2 | 1.11 | (0.97, 1.26) | 0.13 | 1.07 | (0.96, 1.20) | 0.22 | 0.94 | (0.75, 1.17) | 0.59 | 0.85 | (0.66, 1.10) | 0.22 |
| Excessive information | 1 | 1.07 | (0.95, 1.21) | 0.27 | 1.06 | (0.96, 1.18) | 0.26 | 1.03 | (0.84, 1.25) | 0.81 | 0.96 | (0.76, 1.21) | 0.71 |
|  | 2 | 1.09 | (0.97, 1.23) | 0.15 | 1.09 | (0.98, 1.20) | 0.12 | 1.05 | (0.86, 1.28) | 0.62 | 0.96 | (0.76, 1.21) | 0.73 |
| Smoking | 1 | 1.05 | (0.94, 1.18) | 0.36 | 1.07 | (0.97, 1.17) | 0.19 | 1.12 | (0.96, 1.31) | 0.15 | 1.06 | (0.87, 1.29) | 0.55 |
|  | 2 | 1.06 | (0.95, 1.20) | 0.30 | 1.07 | (0.97, 1.19) | 0.16 | 1.12 | (0.96, 1.31) | 0.15 | 1.05 | (0.87, 1.28) | 0.60 |
| Social situation | 1 | 1.05 | (0.94, 1.18) | 0.41 | 1.03 | (0.94, 1.14) | 0.50 | 0.97 | (0.79, 1.20) | 0.78 | 0.93 | (0.72, 1.18) | 0.54 |
|  | 2 | 1.03 | (0.91, 1.16) | 0.64 | 1.02 | (0.93, 1.13) | 0.63 | 1.01 | (0.82, 1.24) | 0.94 | 0.98 | (0.77, 1.25) | 0.87 |
| Poor access to rapid tests | 1 | 1.05 | (0.93, 1.18) | 0.45 | 1.06 | (0.96, 1.17) | 0.25 | 1.12 | (0.91, 1.38) | 0.27 | 1.07 | (0.84, 1.37) | 0.59 |
|  | 2 | 1.05 | (0.93, 1.18) | 0.47 | 1.05 | (0.95, 1.16) | 0.35 | 1.07 | (0.87, 1.31) | 0.52 | 1.02 | (0.80, 1.30) | 0.86 |
| Public sector | 1 | 1.04 | (0.92, 1.18) | 0.51 | 1.04 | (0.94, 1.15) | 0.44 | 1.04 | (0.82, 1.33) | 0.74 | 1.00 | (0.75, 1.33) | 1.00 |
|  | 2 | 1.02 | (0.90, 1.16) | 0.74 | 1.02 | (0.92, 1.14) | 0.70 | 1.02 | (0.80, 1.30) | 0.85 | 1.00 | (0.76, 1.32) | 0.99 |
| Enough deployment training | 1 | 1.04 | (0.91, 1.18) | 0.57 | 1.04 | (0.93, 1.16) | 0.51 | 1.04 | (0.84, 1.28) | 0.71 | 1.00 | (0.78, 1.28) | 0.99 |
|  | 2 | 1.03 | (0.90, 1.18) | 0.71 | 1.03 | (0.91, 1.15) | 0.68 | 1.02 | (0.83, 1.26) | 0.83 | 1.00 | (0.78, 1.27) | 0.98 |
| Current financial stress | 1 | 0.98 | (0.82, 1.17) | 0.83 | 1.03 | (0.89, 1.19) | 0.70 | 1.26 | (0.91, 1.75) | 0.17 | 1.29 | (0.87, 1.89) | 0.21 |
|  | 2 | 0.98 | (0.82, 1.17) | 0.85 | 1.03 | (0.89, 1.20) | 0.70 | 1.26 | (0.91, 1.74) | 0.17 | 1.28 | (0.87, 1.87) | 0.21 |

Table A11. (Cont.) Associations with GAD in full models: with either exclusion of apparent duplicate responses (Group 1), or inclusion of all available responses (Group2)

|  |  | Survey 1 | | | Survey 2 | | | Survey 3 | | | Change in GAD over 1 year ^3^ | | |
| --- | --- | --- | --- | --- | --- | --- | --- | --- | --- | --- | --- | --- | --- |
| Predictors | Group^2^ | OR^1^ | 95%CI | P-value | OR^1^ | 95%CI | P-value | OR^1^ | 95%CI | P-value | OR^1^ | 95%CI | P-value |
| Lower staff grade | 1 | 0.98 | (0.86, 1.10) | 0.68 | 0.97 | (0.88, 1.08) | 0.60 | 0.96 | (0.76, 1.22) | 0.74 | 0.99 | (0.75, 1.30) | 0.92 |
|  | 2 | 0.99 | (0.87, 1.12) | 0.88 | 0.99 | (0.89, 1.10) | 0.88 | 1.00 | (0.80, 1.24) | 0.98 | 1.01 | (0.78, 1.31) | 0.96 |
| Higher education level | 1 | 0.92 | (0.82, 1.04) | 0.17 | 0.91 | (0.82, 1.01) | 0.065 | 0.85 | (0.68, 1.07) | 0.17 | 0.93 | (0.72, 1.20) | 0.57 |
|  | 2 | 0.93 | (0.82, 1.06) | 0.29 | 0.92 | (0.83, 1.03) | 0.14 | 0.87 | (0.70, 1.07) | 0.18 | 0.93 | (0.73, 1.18) | 0.54 |
| Intensity of exposure | 1 | 0.90 | (0.79, 1.03) | 0.14 | 0.94 | (0.83, 1.05) | 0.27 | 1.09 | (0.87, 1.36) | 0.46 | 1.20 | (0.93, 1.56) | 0.17 |
|  | 2 | 0.91 | (0.79, 1.04) | 0.18 | 0.95 | (0.84, 1.06) | 0.35 | 1.11 | (0.90, 1.37) | 0.32 | 1.22 | (0.95, 1.57) | 0.12 |
| Males | 1 | 0.83 | (0.74, 0.94) | 0.002 | 0.84 | (0.76, 0.93) | 0.001 | 0.86 | (0.69, 1.08) | 0.20 | 1.03 | (0.80, 1.33) | 0.79 |
|  | 2 | 0.85 | (0.76, 0.96) | 0.007 | 0.85 | (0.76, 0.94) | 0.002 | 0.84 | (0.68, 1.03) | 0.088 | 0.98 | (0.78, 1.24) | 0.88 |
| Age group | 1 | 0.73 | (0.64, 0.83) | <0.001 | 0.71 | (0.64, 0.79) | <0.001 | 0.63 | (0.51, 0.78) | <0.001 | 0.86 | (0.67, 1.11) | 0.25 |
|  | 2 | 0.75 | (0.66, 0.85) | <0.001 | 0.72 | (0.64, 0.81) | <0.001 | 0.61 | (0.49, 0.75) | <0.001 | 0.81 | (0.63, 1.04) | 0.10 |

Table A12. Associations with ISI in full models: with either exclusion of apparent duplicate responses (Group 1), or inclusion of all available responses (Group2)

|  |  | Survey 1 | | | Survey 2 | | | Survey 3 | | | Change in ISI over 1 year ^3^ | | |
| --- | --- | --- | --- | --- | --- | --- | --- | --- | --- | --- | --- | --- | --- |
|  | Group^2^ | OR^1^ | 95%CI | P-value | OR^1^ | 95%CI | P-value | OR^1^ | 95%CI | P-value | OR^1^ | 95%CI | P-value |
| Change in ISI7 over 1 year ^3^ | 1 |  |  |  |  |  |  |  |  |  | 1.43 | (1.15, 1.78) | 0.001 |
|  | 2 |  |  |  |  |  |  |  |  |  | 1.42 | (1.13, 1.77) | 0.002 |
| Predictors |  |  |  |  |  |  |  |  |  |  |  |  |  |
| Current home/family stress | 1 | 1.64 | (1.39, 1.94) | <0.001 | 1.58 | (1.38, 1.82) | <0.001 | 1.35 | (1.06, 1.73) | 0.015 | 0.83 | (0.61, 1.12) | 0.22 |
|  | 2 | 1.63 | (1.38, 1.92) | <0.001 | 1.57 | (1.37, 1.81) | <0.001 | 1.35 | (1.04, 1.74) | 0.022 | 0.83 | (0.61, 1.13) | 0.24 |
| Future home/family stress | 1 | 1.29 | (1.10, 1.52) | 0.002 | 1.21 | (1.06, 1.39) | 0.005 | 0.93 | (0.73, 1.18) | 0.55 | 0.72 | (0.53, 0.97) | 0.034 |
|  | 2 | 1.26 | (1.07, 1.49) | 0.007 | 1.18 | (1.02, 1.35) | 0.022 | 0.88 | (0.69, 1.13) | 0.32 | 0.70 | (0.51, 0.96) | 0.025 |
| Poor clinical team support | 1 | 1.19 | (1.04, 1.36) | 0.012 | 1.25 | (1.11, 1.40) | <0.001 | 1.54 | (1.25, 1.89) | <0.001 | 1.29 | (1.01, 1.66) | 0.046 |
|  | 2 | 1.19 | (1.04, 1.37) | 0.013 | 1.25 | (1.11, 1.41) | <0.001 | 1.51 | (1.23, 1.85) | <0.001 | 1.26 | (0.99, 1.62) | 0.064 |
| Poor access to rapid tests | 1 | 1.16 | (1.02, 1.32) | 0.026 | 1.16 | (1.04, 1.30) | 0.007 | 1.18 | (0.97, 1.44) | 0.089 | 1.02 | (0.80, 1.30) | 0.87 |
|  | 2 | 1.17 | (1.03, 1.34) | 0.018 | 1.18 | (1.05, 1.32) | 0.005 | 1.18 | (0.97, 1.43) | 0.10 | 1.00 | (0.79, 1.28) | 0.98 |
| Inadequacy of information | 1 | 1.13 | (0.98, 1.31) | 0.10 | 1.11 | (0.98, 1.25) | 0.11 | 1.00 | (0.80, 1.26) | 0.97 | 0.89 | (0.67, 1.17) | 0.41 |
|  | 2 | 1.12 | (0.96, 1.31) | 0.14 | 1.11 | (0.97, 1.26) | 0.12 | 1.05 | (0.84, 1.32) | 0.67 | 0.94 | (0.71, 1.24) | 0.64 |

Table A12. (Cont.) Associations with ISI in full models: with either exclusion of apparent duplicate responses (Group 1), or inclusion of all available responses (Group2)

|  |  | Survey 1 | | | Survey 2 | | | Survey 3 | | | Change in ISI over 1 year ^3^ | | |
| --- | --- | --- | --- | --- | --- | --- | --- | --- | --- | --- | --- | --- | --- |
| Predictors | Group^2^ | OR^1^ | 95%CI | P-value | OR^1^ | 95%CI | P-value | OR^1^ | 95%CI | P-value | OR^1^ | 95%CI | P-value |
| Current financial stress | 1 | 1.12 | (0.94, 1.33) | 0.20 | 1.17 | (1.01, 1.35) | 0.036 | 1.39 | (1.06, 1.83) | 0.017 | 1.24 | (0.89, 1.73) | 0.20 |
|  | 2 | 1.14 | (0.95, 1.35) | 0.16 | 1.18 | (1.02, 1.37) | 0.030 | 1.38 | (1.05, 1.80) | 0.021 | 1.21 | (0.87, 1.69) | 0.26 |
| Concerned about PPE | 1 | 1.12 | (0.98, 1.27) | 0.10 | 1.13 | (1.01, 1.26) | 0.034 | 1.18 | (0.97, 1.43) | 0.10 | 1.06 | (0.83, 1.34) | 0.66 |
|  | 2 | 1.13 | (0.98, 1.29) | 0.088 | 1.13 | (1.01, 1.27) | 0.039 | 1.14 | (0.94, 1.39) | 0.18 | 1.02 | (0.80, 1.29) | 0.90 |
| Covid ward status | 1 | 1.10 | (0.96, 1.26) | 0.19 | 1.07 | (0.94, 1.21) | 0.30 | 0.95 | (0.75, 1.20) | 0.66 | 0.86 | (0.66, 1.14) | 0.30 |
|  | 2 | 1.10 | (0.95, 1.26) | 0.19 | 1.07 | (0.94, 1.21) | 0.31 | 0.95 | (0.74, 1.21) | 0.67 | 0.86 | (0.65, 1.14) | 0.31 |
| Poor ability for quality care | 1 | 1.09 | (0.96, 1.24) | 0.20 | 1.08 | (0.96, 1.21) | 0.19 | 1.03 | (0.80, 1.33) | 0.81 | 0.95 | (0.70, 1.27) | 0.72 |
|  | 2 | 1.12 | (0.98, 1.28) | 0.096 | 1.11 | (0.99, 1.24) | 0.082 | 1.05 | (0.82, 1.35) | 0.70 | 0.94 | (0.70, 1.26) | 0.67 |
| Enough deployment training | 1 | 1.08 | (0.94, 1.24) | 0.29 | 1.08 | (0.96, 1.21) | 0.21 | 1.09 | (0.89, 1.33) | 0.43 | 1.01 | (0.79, 1.29) | 0.94 |
|  | 2 | 1.09 | (0.94, 1.25) | 0.26 | 1.08 | (0.96, 1.22) | 0.21 | 1.06 | (0.86, 1.30) | 0.60 | 0.97 | (0.76, 1.26) | 0.84 |
| Social situation | 1 | 1.04 | (0.92, 1.18) | 0.55 | 1.04 | (0.93, 1.15) | 0.50 | 1.03 | (0.87, 1.21) | 0.75 | 0.99 | (0.80, 1.21) | 0.91 |
|  | 2 | 1.01 | (0.89, 1.15) | 0.88 | 1.02 | (0.91, 1.14) | 0.76 | 1.05 | (0.89, 1.24) | 0.55 | 1.04 | (0.84, 1.28) | 0.71 |
| Public sector | 1 | 1.04 | (0.91, 1.17) | 0.58 | 1.07 | (0.96, 1.19) | 0.22 | 1.22 | (0.99, 1.49) | 0.058 | 1.17 | (0.92, 1.50) | 0.20 |
|  | 2 | 1.03 | (0.90, 1.18) | 0.65 | 1.06 | (0.95, 1.20) | 0.30 | 1.21 | (0.99, 1.49) | 0.068 | 1.17 | (0.91, 1.50) | 0.21 |
| Future financial stress | 1 | 1.03 | (0.87, 1.23) | 0.72 | 1.05 | (0.90, 1.21) | 0.54 | 1.11 | (0.85, 1.46) | 0.44 | 1.08 | (0.77, 1.51) | 0.66 |
|  | 2 | 1.04 | (0.87, 1.24) | 0.68 | 1.06 | (0.91, 1.23) | 0.44 | 1.16 | (0.88, 1.52) | 0.30 | 1.11 | (0.79, 1.57) | 0.54 |
| Excessive information | 1 | 1.00 | (0.89, 1.13) | 0.97 | 1.03 | (0.93, 1.13) | 0.62 | 1.13 | (0.92, 1.38) | 0.25 | 1.12 | (0.88, 1.43) | 0.34 |
|  | 2 | 1.03 | (0.91, 1.15) | 0.66 | 1.05 | (0.95, 1.16) | 0.33 | 1.16 | (0.94, 1.43) | 0.16 | 1.13 | (0.88, 1.44) | 0.33 |
| Lower staff grade | 1 | 0.99 | (0.88, 1.11) | 0.84 | 0.97 | (0.88, 1.07) | 0.58 | 0.91 | (0.76, 1.09) | 0.32 | 0.92 | (0.74, 1.15) | 0.48 |
|  | 2 | 1.00 | (0.89, 1.12) | 0.99 | 0.98 | (0.89, 1.09) | 0.76 | 0.92 | (0.76, 1.11) | 0.37 | 0.92 | (0.73, 1.15) | 0.46 |
| Males | 1 | 0.98 | (0.87, 1.10) | 0.75 | 0.95 | (0.86, 1.05) | 0.33 | 0.83 | (0.67, 1.02) | 0.083 | 0.84 | (0.66, 1.07) | 0.17 |
|  | 2 | 0.99 | (0.88, 1.12) | 0.89 | 0.96 | (0.86, 1.06) | 0.42 | 0.83 | (0.67, 1.01) | 0.066 | 0.83 | (0.66, 1.05) | 0.13 |
| Smoking | 1 | 0.96 | (0.84, 1.10) | 0.59 | 0.99 | (0.89, 1.11) | 0.90 | 1.12 | (0.96, 1.31) | 0.14 | 1.16 | (0.96, 1.42) | 0.13 |
|  | 2 | 0.99 | (0.86, 1.14) | 0.90 | 1.02 | (0.90, 1.15) | 0.80 | 1.13 | (0.97, 1.32) | 0.12 | 1.14 | (0.93, 1.40) | 0.20 |
| Age group | 1 | 0.95 | (0.84, 1.07) | 0.41 | 0.94 | (0.85, 1.04) | 0.22 | 0.89 | (0.73, 1.07) | 0.22 | 0.93 | (0.74, 1.17) | 0.55 |
|  | 2 | 0.98 | (0.87, 1.10) | 0.71 | 0.96 | (0.86, 1.06) | 0.42 | 0.88 | (0.72, 1.07) | 0.21 | 0.90 | (0.71, 1.14) | 0.37 |
| Intensity of exposure | 1 | 0.94 | (0.81, 1.10) | 0.46 | 0.98 | (0.86, 1.11) | 0.74 | 1.14 | (0.93, 1.39) | 0.22 | 1.20 | (0.93, 1.56) | 0.16 |
|  | 2 | 0.95 | (0.82, 1.11) | 0.55 | 0.99 | (0.87, 1.12) | 0.86 | 1.15 | (0.94, 1.40) | 0.19 | 1.20 | (0.93, 1.55) | 0.17 |
| Higher education level | 1 | 0.75 | (0.66, 0.85) | <0.001 | 0.75 | (0.67, 0.84) | <0.001 | 0.78 | (0.63, 0.96) | 0.018 | 1.04 | (0.82, 1.33) | 0.75 |
|  | 2 | 0.76 | (0.67, 0.87) | <0.001 | 0.77 | (0.69, 0.86) | <0.001 | 0.79 | (0.65, 0.97) | 0.022 | 1.03 | (0.82, 1.31) | 0.78 |

Table A13. Associations with Impact of Events Scale – Revised (IES-R) in full models: with either exclusion of apparent duplicate responses (Group 1), or inclusion of all available responses (Group2)

|  |  | Survey 1 | | | Survey 2 | | | Survey 3 | | | Change in IES-R over 1 year ^3^ | | |
| --- | --- | --- | --- | --- | --- | --- | --- | --- | --- | --- | --- | --- | --- |
|  | Group^2^ | OR^1^ | 95%CI | P-value | OR^1^ | 95%CI | P-value | OR^1^ | 95%CI | P-value | OR^1^ | 95%CI | P-value |
| Change in IES-R over 1 year^3^ | 1 |  |  |  |  |  |  |  |  |  | 0.66 | (0.52, 0.83) | <0.001 |
|  | 2 |  |  |  |  |  |  |  |  |  | 0.64 | (0.51, 0.81) | <0.001 |
| Predictors |  |  |  |  |  |  |  |  |  |  |  |  |  |
| Current home/family stress | 1 | 1.95 | (1.64, 2.33) | <0.001 | 1.89 | (1.63, 2.18) | <0.001 | 1.64 | (1.30, 2.06) | <0.001 | 0.84 | (0.62, 1.13) | 0.25 |
|  | 2 | 1.92 | (1.60, 2.30) | <0.001 | 1.85 | (1.59, 2.14) | <0.001 | 1.57 | (1.24, 1.99) | <0.001 | 0.82 | (0.60, 1.11) | 0.20 |
| Poor clinical team support | 1 | 1.34 | (1.16, 1.54) | <0.001 | 1.39 | (1.23, 1.57) | <0.001 | 1.62 | (1.29, 2.05) | <0.001 | 1.21 | (0.92, 1.60) | 0.17 |
|  | 2 | 1.31 | (1.13, 1.52) | <0.001 | 1.37 | (1.21, 1.55) | <0.001 | 1.61 | (1.29, 2.01) | <0.001 | 1.23 | (0.94, 1.60) | 0.13 |
| Concerned about PPE | 1 | 1.30 | (1.14, 1.47) | <0.001 | 1.28 | (1.15, 1.42) | <0.001 | 1.19 | (0.98, 1.46) | 0.082 | 0.92 | (0.72, 1.17) | 0.50 |
|  | 2 | 1.28 | (1.13, 1.46) | <0.001 | 1.27 | (1.14, 1.41) | <0.001 | 1.22 | (1.00, 1.48) | 0.049 | 0.95 | (0.75, 1.21) | 0.67 |
| Future home/family stress | 1 | 1.26 | (1.06, 1.49) | 0.009 | 1.22 | (1.07, 1.41) | 0.004 | 1.09 | (0.84, 1.42) | 0.50 | 0.87 | (0.62, 1.22) | 0.42 |
|  | 2 | 1.24 | (1.04, 1.47) | 0.016 | 1.22 | (1.06, 1.40) | 0.006 | 1.13 | (0.87, 1.46) | 0.36 | 0.91 | (0.65, 1.28) | 0.59 |
| Inadequacy of information | 1 | 1.19 | (1.04, 1.35) | 0.010 | 1.18 | (1.06, 1.32) | 0.003 | 1.16 | (0.93, 1.46) | 0.18 | 0.98 | (0.75, 1.28) | 0.90 |
|  | 2 | 1.18 | (1.03, 1.34) | 0.016 | 1.18 | (1.05, 1.32) | 0.004 | 1.19 | (0.95, 1.49) | 0.14 | 1.01 | (0.77, 1.32) | 0.94 |
| Poor ability for quality care | 1 | 1.15 | (1.01, 1.32) | 0.039 | 1.12 | (0.99, 1.25) | 0.065 | 0.97 | (0.75, 1.25) | 0.80 | 0.84 | (0.62, 1.13) | 0.25 |
|  | 2 | 1.22 | (1.06, 1.40) | 0.005 | 1.18 | (1.05, 1.33) | 0.006 | 1.02 | (0.79, 1.31) | 0.90 | 0.83 | (0.62, 1.12) | 0.23 |
| Future financial stress | 1 | 1.12 | (0.94, 1.35) | 0.21 | 1.15 | (0.99, 1.34) | 0.069 | 1.27 | (0.94, 1.72) | 0.12 | 1.13 | (0.78, 1.63) | 0.52 |
|  | 2 | 1.16 | (0.97, 1.38) | 0.10 | 1.17 | (1.01, 1.35) | 0.041 | 1.20 | (0.89, 1.63) | 0.24 | 1.04 | (0.72, 1.50) | 0.84 |
| Social situation | 1 | 1.12 | (0.99, 1.27) | 0.073 | 1.10 | (0.99, 1.22) | 0.089 | 0.99 | (0.83, 1.19) | 0.95 | 0.89 | (0.71, 1.11) | 0.29 |
|  | 2 | 1.09 | (0.96, 1.24) | 0.20 | 1.07 | (0.96, 1.19) | 0.21 | 1.00 | (0.83, 1.20) | 0.99 | 0.92 | (0.73, 1.16) | 0.47 |
| Excessive information | 1 | 1.10 | (0.97, 1.25) | 0.13 | 1.10 | (0.99, 1.22) | 0.077 | 1.10 | (0.91, 1.31) | 0.33 | 0.99 | (0.80, 1.24) | 0.96 |
|  | 2 | 1.12 | (0.98, 1.27) | 0.090 | 1.11 | (1.00, 1.24) | 0.052 | 1.10 | (0.91, 1.32) | 0.34 | 0.98 | (0.78, 1.24) | 0.88 |
| Current financial stress | 1 | 1.08 | (0.90, 1.29) | 0.39 | 1.13 | (0.97, 1.31) | 0.12 | 1.34 | (1.00, 1.79) | 0.052 | 1.23 | (0.87, 1.76) | 0.25 |
|  | 2 | 1.07 | (0.90, 1.29) | 0.44 | 1.13 | (0.97, 1.31) | 0.12 | 1.39 | (1.03, 1.86) | 0.029 | 1.29 | (0.90, 1.84) | 0.16 |
| Covid ward status | 1 | 1.08 | (0.95, 1.23) | 0.26 | 1.06 | (0.95, 1.19) | 0.29 | 1.01 | (0.79, 1.29) | 0.91 | 0.94 | (0.72, 1.24) | 0.67 |
|  | 2 | 1.08 | (0.94, 1.23) | 0.28 | 1.07 | (0.95, 1.20) | 0.30 | 1.02 | (0.79, 1.32) | 0.87 | 0.95 | (0.72, 1.26) | 0.71 |
| Public sector | 1 | 1.04 | (0.90, 1.21) | 0.59 | 1.04 | (0.92, 1.18) | 0.50 | 1.05 | (0.89, 1.25) | 0.54 | 1.01 | (0.81, 1.27) | 0.91 |
|  | 2 | 1.03 | (0.88, 1.19) | 0.75 | 1.03 | (0.91, 1.17) | 0.64 | 1.05 | (0.89, 1.25) | 0.56 | 1.03 | (0.82, 1.29) | 0.83 |
| Intensity of exposure | 1 | 1.04 | (0.90, 1.19) | 0.63 | 1.06 | (0.94, 1.20) | 0.32 | 1.19 | (0.96, 1.47) | 0.12 | 1.15 | (0.88, 1.49) | 0.30 |
|  | 2 | 1.04 | (0.90, 1.19) | 0.59 | 1.07 | (0.95, 1.20) | 0.29 | 1.19 | (0.96, 1.47) | 0.11 | 1.14 | (0.89, 1.48) | 0.30 |
| Enough deployment training | 1 | 1.02 | (0.90, 1.17) | 0.71 | 1.06 | (0.95, 1.18) | 0.33 | 1.21 | (0.98, 1.48) | 0.071 | 1.17 | (0.93, 1.49) | 0.18 |
|  | 2 | 1.00 | (0.88, 1.15) | 0.96 | 1.03 | (0.92, 1.16) | 0.59 | 1.17 | (0.95, 1.43) | 0.15 | 1.16 | (0.91, 1.47) | 0.22 |

Table A13. (Cont.) Associations with IES-R in full models: with either exclusion of apparent duplicate responses (Group 1), or inclusion of all available responses (Group2)

|  |  | Survey 1 | | | Survey 2 | | | Survey 3 | | | | | Change in IES-R over 1 year ^3^ | | | | |  |
| --- | --- | --- | --- | --- | --- | --- | --- | --- | --- | --- | --- | --- | --- | --- | --- | --- | --- | --- |
| Predictors | Group^2^ | OR^1^ | 95%CI | P-value | OR^1^ | 95%CI | P-value | OR^1^ | 95%CI | | P-value | | OR^1^ | | 95%CI | | P-value |  |
| Smoking | 1 | 1.00 | (0.90, 1.13) | 0.94 | 1.04 | (0.94, 1.14) | 0.45 | 1.19 | (0.97, 1.47) | 0.098 | | 1.19 | | (0.93, 1.52) | | 0.18 | | |
|  | 2 | 1.05 | (0.94, 1.18) | 0.38 | 1.08 | (0.98, 1.19) | 0.13 | 1.20 | (0.97, 1.48) | 0.088 | | 1.14 | | (0.89, 1.46) | | 0.30 | | |
| Poor access to rapid tests | 1 | 0.96 | (0.84, 1.09) | 0.51 | 0.97 | (0.87, 1.08) | 0.55 | 1.01 | (0.81, 1.25) | | 0.94 | | 1.05 | | (0.81, 1.36) | | 0.70 |  |
|  | 2 | 0.95 | (0.84, 1.09) | 0.48 | 0.96 | (0.86, 1.07) | 0.46 | 0.98 | (0.79, 1.22) | | 0.86 | | 1.03 | | (0.79, 1.34) | | 0.85 |  |
| Age group | 1 | 0.96 | (0.84, 1.09) | 0.51 | 0.96 | (0.86, 1.07) | 0.45 | 0.96 | (0.79, 1.17) | | 0.70 | | 1.00 | | (0.79, 1.28) | | 0.97 |  |
|  | 2 | 0.96 | (0.84, 1.09) | 0.52 | 0.96 | (0.86, 1.07) | 0.45 | 0.96 | (0.77, 1.18) | | 0.67 | | 1.00 | | (0.78, 1.28) | | 0.98 |  |
| Lower staff grade | 1 | 0.90 | (0.79, 1.02) | 0.090 | 0.90 | (0.81, 0.99) | 0.039 | 0.89 | (0.74, 1.06) | | 0.20 | | 0.99 | | (0.79, 1.24) | | 0.91 |  |
|  | 2 | 0.90 | (0.79, 1.02) | 0.094 | 0.90 | (0.80, 1.00) | 0.045 | 0.89 | (0.73, 1.07) | | 0.22 | | 0.99 | | (0.79, 1.25) | | 0.94 |  |
| Higher education level | 1 | 0.87 | (0.77, 0.99) | 0.031 | 0.86 | (0.78, 0.96) | 0.009 | 0.84 | (0.69, 1.02) | | 0.074 | | 0.96 | | (0.77, 1.21) | | 0.75 |  |
|  | 2 | 0.88 | (0.78, 1.00) | 0.053 | 0.87 | (0.78, 0.97) | 0.013 | 0.82 | (0.68, 0.99) | | 0.044 | | 0.93 | | (0.74, 1.17) | | 0.55 |  |
| Males | 1 | 0.79 | (0.70, 0.88) | <0.001 | 0.81 | (0.73, 0.89) | <0.001 | 0.90 | (0.72, 1.13) | | 0.36 | | 1.14 | | (0.89, 1.48) | | 0.30 |  |
|  | 2 | 0.79 | (0.70, 0.89) | <0.001 | 0.80 | (0.72, 0.89) | <0.001 | 0.85 | (0.70, 1.04) | | 0.12 | | 1.08 | | (0.85, 1.37) | | 0.51 |  |

Table A14. Associations with Patient Health Questionnaire (PHQ) in full models: with either exclusion of apparent duplicate responses (Group 1), or inclusion of all available responses (Group2)

|  |  | Survey 1 | | | Survey 2 | | | Survey 3 | | | Change in PHQ over 1 year ^3^ | | |
| --- | --- | --- | --- | --- | --- | --- | --- | --- | --- | --- | --- | --- | --- |
| Predictors | Group^2^ | OR^1^ | 95%CI | P-value | OR^1^ | 95%CI | P-value | OR^1^ | 95%CI | P-value | OR^1^ | 95%CI | P-value |
| Change in PHQ over 1 year ^3^ | 1 |  |  |  |  |  |  |  |  |  | 1.24 | (0.97, 1.59) | 0.079 |
|  | 2 |  |  |  |  |  |  |  |  |  | 1.21 | (0.94, 1.56) | 0.14 |
| Current home/family stress | 1 | 1.67 | (1.38, 2.01) | <0.001 | 1.61 | (1.38, 1.88) | <0.001 | 1.38 | (1.08, 1.76) | 0.010 | 0.83 | (0.61, 1.14) | 0.24 |
|  | 2 | 1.63 | (1.35, 1.97) | <0.001 | 1.57 | (1.34, 1.84) | <0.001 | 1.34 | (1.04, 1.73) | 0.022 | 0.82 | (0.60, 1.14) | 0.24 |
| Future home/family stress | 1 | 1.26 | (1.05, 1.50) | 0.014 | 1.20 | (1.03, 1.40) | 0.017 | 1.00 | (0.77, 1.30) | 1.00 | 0.80 | (0.58, 1.10) | 0.17 |
|  | 2 | 1.26 | (1.04, 1.52) | 0.018 | 1.20 | (1.03, 1.41) | 0.021 | 1.00 | (0.77, 1.30) | 1.00 | 0.80 | (0.57, 1.11) | 0.18 |
| Social situation | 1 | 1.20 | (1.05, 1.38) | 0.006 | 1.20 | (1.07, 1.34) | 0.002 | 1.17 | (0.96, 1.43) | 0.13 | 0.97 | (0.76, 1.24) | 0.82 |
|  | 2 | 1.16 | (1.02, 1.32) | 0.026 | 1.17 | (1.04, 1.30) | 0.007 | 1.19 | (0.96, 1.46) | 0.11 | 1.02 | (0.79, 1.31) | 0.87 |
| Poor clinical team support | 1 | 1.18 | (1.03, 1.37) | 0.021 | 1.28 | (1.13, 1.44) | <0.001 | 1.75 | (1.40, 2.19) | <0.001 | 1.48 | (1.12, 1.94) | 0.005 |
|  | 2 | 1.19 | (1.03, 1.38) | 0.019 | 1.27 | (1.13, 1.44) | <0.001 | 1.71 | (1.37, 2.12) | <0.001 | 1.43 | (1.10, 1.86) | 0.008 |
| Inadequacy of information | 1 | 1.18 | (1.01, 1.37) | 0.032 | 1.17 | (1.04, 1.33) | 0.012 | 1.15 | (0.91, 1.46) | 0.24 | 0.98 | (0.73, 1.31) | 0.88 |
|  | 2 | 1.16 | (1.00, 1.35) | 0.053 | 1.17 | (1.03, 1.33) | 0.017 | 1.19 | (0.94, 1.52) | 0.15 | 1.03 | (0.76, 1.38) | 0.86 |
| Poor ability for quality care | 1 | 1.17 | (1.03, 1.34) | 0.019 | 1.15 | (1.02, 1.29) | 0.022 | 1.05 | (0.82, 1.35) | 0.71 | 0.90 | (0.68, 1.19) | 0.44 |
|  | 2 | 1.21 | (1.05, 1.39) | 0.009 | 1.19 | (1.05, 1.35) | 0.006 | 1.12 | (0.88, 1.42) | 0.38 | 0.92 | (0.70, 1.22) | 0.58 |

Table A14. (Cont.) Associations with PHQ9 in full models: with either exclusion of apparent duplicate responses (Group 1), or inclusion of all available responses (Group2)

|  |  | Survey 1 | | | Survey 2 | | | | | Survey 3 | | | | Change in PHQ9 over 1 year ^3^ | | |
| --- | --- | --- | --- | --- | --- | --- | --- | --- | --- | --- | --- | --- | --- | --- | --- | --- |
| Predictors | Group^2^ | OR^1^ | 95%CI | P-value | OR^1^ | | 95%CI | | P-value | OR^1^ | 95%CI | P-value | | OR^1^ | 95%CI | P-value |
| Enough deployment training | 1 | 1.10 | (0.94, 1.28) | 0.22 | 1.11 | (0.97, 1.26) | | 0.12 | | 1.15 | (0.95, 1.39) | | 0.16 | 1.04 | (0.82, 1.33) | 0.74 |
|  | 2 | 1.09 | (0.93, 1.29) | 0.28 | 1.10 | (0.96, 1.26) | | 0.19 | | 1.12 | (0.92, 1.35) | | 0.26 | 1.02 | (0.80, 1.31) | 0.86 |
| Current financial stress | 1 | 1.08 | (0.90, 1.29) | 0.42 | 1.08 | (0.93, 1.25) | | 0.32 | | 1.09 | (0.80, 1.48) | | 0.58 | 1.01 | (0.70, 1.47) | 0.94 |
|  | 2 | 1.07 | (0.90, 1.29) | 0.44 | 1.08 | (0.93, 1.26) | | 0.31 | | 1.12 | (0.82, 1.53) | | 0.47 | 1.04 | (0.72, 1.51) | 0.82 |
| Future financial stress | 1 | 1.08 | (0.90, 1.28) | 0.42 | 1.10 | | (0.95, 1.27) | | 0.22 | 1.20 | (0.86, 1.67) | 0.29 | | 1.11 | (0.75, 1.65) | 0.60 |
|  | 2 | 1.09 | (0.91, 1.30) | 0.36 | 1.10 | | (0.95, 1.28) | | 0.20 | 1.18 | (0.84, 1.65) | 0.34 | | 1.08 | (0.72, 1.62) | 0.70 |
| Excessive information | 1 | 1.07 | (0.93, 1.22) | 0.35 | 1.07 | | (0.95, 1.19) | | 0.26 | 1.07 | (0.89, 1.30) | 0.46 | | 1.01 | (0.80, 1.27) | 0.95 |
|  | 2 | 1.10 | (0.96, 1.25) | 0.18 | 1.10 | | (0.98, 1.22) | | 0.11 | 1.09 | (0.90, 1.32) | 0.38 | | 1.00 | (0.78, 1.26) | 0.97 |
| Concerned about PPE | 1 | 1.05 | (0.92, 1.20) | 0.43 | 1.08 | | (0.97, 1.21) | | 0.17 | 1.20 | (0.98, 1.49) | 0.083 | | 1.14 | (0.89, 1.47) | 0.31 |
|  | 2 | 1.07 | (0.93, 1.23) | 0.34 | 1.09 | | (0.97, 1.23) | | 0.15 | 1.19 | (0.97, 1.47) | 0.10 | | 1.11 | (0.86, 1.43) | 0.41 |
| Smoking | 1 | 1.05 | (0.94, 1.18) | 0.39 | 1.07 | | (0.97, 1.18) | | 0.20 | 1.13 | (0.93, 1.37) | 0.22 | | 1.07 | (0.85, 1.35) | 0.55 |
|  | 2 | 1.09 | (0.97, 1.22) | 0.15 | 1.10 | | (1.00, 1.21) | | 0.062 | 1.14 | (0.94, 1.38) | 0.19 | | 1.05 | (0.83, 1.32) | 0.70 |
| Covid ward status | 1 | 1.04 | (0.91, 1.19) | 0.55 | 1.02 | | (0.91, 1.15) | | 0.71 | 0.95 | (0.74, 1.21) | 0.65 | | 0.91 | (0.69, 1.20) | 0.49 |
|  | 2 | 1.04 | (0.90, 1.20) | 0.60 | 1.02 | | (0.90, 1.15) | | 0.73 | 0.95 | (0.74, 1.22) | 0.69 | | 0.92 | (0.69, 1.22) | 0.55 |
| Public sector | 1 | 1.04 | (0.90, 1.21) | 0.60 | 1.02 | | (0.90, 1.15) | | 0.78 | 0.93 | (0.74, 1.16) | 0.52 | | 0.89 | (0.68, 1.17) | 0.41 |
|  | 2 | 1.01 | (0.86, 1.18) | 0.93 | 0.99 | | (0.86, 1.13) | | 0.86 | 0.91 | (0.73, 1.14) | 0.41 | | 0.91 | (0.69, 1.18) | 0.47 |
| Intensity of exposure | 1 | 1.01 | (0.88, 1.17) | 0.87 | 1.03 | | (0.91, 1.16) | | 0.65 | 1.10 | (0.89, 1.37) | 0.38 | | 1.09 | (0.83, 1.42) | 0.53 |
|  | 2 | 1.03 | (0.89, 1.19) | 0.67 | 1.05 | | (0.93, 1.18) | | 0.46 | 1.12 | (0.90, 1.39) | 0.32 | | 1.08 | (0.83, 1.41) | 0.57 |
| Poor access to rapid tests | 1 | 0.98 | (0.86, 1.11) | 0.71 | 0.99 | | (0.89, 1.10) | | 0.85 | 1.05 | (0.85, 1.29) | 0.67 | | 1.07 | (0.83, 1.39) | 0.59 |
|  | 2 | 0.96 | (0.84, 1.10) | 0.56 | 0.97 | | (0.87, 1.08) | | 0.58 | 1.00 | (0.81, 1.24) | 0.98 | | 1.04 | (0.81, 1.35) | 0.75 |
| Higher education level | 1 | 0.95 | (0.83, 1.08) | 0.43 | 0.93 | | (0.83, 1.04) | | 0.20 | 0.85 | (0.69, 1.04) | 0.11 | | 0.90 | (0.71, 1.14) | 0.36 |
|  | 2 | 0.97 | (0.85, 1.11) | 0.65 | 0.95 | | (0.84, 1.06) | | 0.36 | 0.86 | (0.70, 1.05) | 0.13 | | 0.89 | (0.70, 1.12) | 0.32 |
| Lower staff grade | 1 | 0.93 | (0.83, 1.05) | 0.27 | 0.95 | | (0.86, 1.05) | | 0.29 | 1.00 | (0.82, 1.22) | 0.98 | | 1.07 | (0.84, 1.36) | 0.57 |
|  | 2 | 0.94 | (0.83, 1.07) | 0.37 | 0.96 | | (0.86, 1.07) | | 0.44 | 1.03 | (0.84, 1.26) | 0.80 | | 1.09 | (0.85, 1.39) | 0.51 |
| Males | 1 | 0.87 | (0.76, 0.99) | 0.040 | 0.87 | | (0.78, 0.97) | | 0.016 | 0.88 | (0.70, 1.11) | 0.28 | | 1.01 | (0.76, 1.34) | 0.95 |
|  | 2 | 0.88 | (0.77, 1.01) | 0.066 | 0.87 | | (0.78, 0.98) | | 0.018 | 0.84 | (0.68, 1.05) | 0.12 | | 0.96 | (0.73, 1.25) | 0.74 |
| Age group | 1 | 0.76 | (0.66, 0.87) | <0.001 | 0.75 | | (0.66, 0.84) | | <0.001 | 0.71 | (0.58, 0.87) | 0.001 | | 0.94 | (0.73, 1.21) | 0.62 |
|  | 2 | 0.78 | (0.67, 0.89) | <0.001 | 0.76 | | (0.68, 0.86) | | <0.001 | 0.70 | (0.57, 0.87) | 0.001 | | 0.91 | (0.70, 1.17) | 0.46 |

Footnotes to Tables S11 to S14

1. Associations between predictors and outcomes, and change in outcomes over 1 year were estimated using repeated-measures ordered logistic regression; the effects were shown as odds ratios (OR; 95% confidence intervals; P-values), adjusted for each of the predictors shown in the tables; missing data was substituted by multiple imputation. Odds ratios of 1.00 indicate no association; OR more than 1.00 indicate a positive association; OR less than 1.00 indicate a negative association. The odds ratio values shown are the effect of a rise of 1 standard deviation of each of the predictor variables.
2. Two models are presented: 1) the full model applied to the 1,676 respondent, with the duplicates removed (Group 1, light green shading); 2) the full model applied to the 1,815 responses, with the duplicates included (Group 2, light blue shading)
3. The association between predictors and outcomes at the start of the pandemic (1^st^ April 2020) was fixed by the zero value of the time interaction predictor.
4. The change in association between each predictor and outcomes was determined by the time interaction, with the time defined as the number of years from 1^st^ April 2020 to the date each respondent completed each of the surveys. Mean date of survey completion was: Survey 1, 0.1 years on 6/05/2020; Survey 2, 0.29 years on 13/07/2020; Survey 3, 1.1 years 8/05/2021.
