# Supplementary material for: Stressors, manifestations and course of COVID-19 related distress among public sector nurses and midwives during the COVID-19 pandemic first year in Tasmania, Australia
Source: PLoS One. 2022 Aug 9;17(8):e0271824. doi: 10.1371/journal.pone.0271824 (PMC9362919; doi:10.1371/journal.pone.0271824)
Supplement: Attachment — Submitted filename: Comments.docx [file pone.0271824.s004.docx]

**PONE-D-21-36897**

Thank you for your contribution to the PLOS ONE.

This study describes the critical stressors and how they manifest within both the work and larger social environment for nurses and midwives in Australia.

I have following comments to the authors

**Abstract**

There seems to be a lack of definition in the text of organizational burnout. The Manuscript contains few statements on nurses’ burnout. As burnout was not directly measured in this study, it is necessary to provide an additional explanation about the relationship between burnout and psychological distress investigated in this study

Page 3, the full terminology of PTSD is required.

**Method**

Page 6, participants – It needs additional information on how many people were surveyed and what the response rate was.

Page 8, line 196-7 & page 9, line 205-207 – They seem unnecessary as measurement descriptions

The description of qualitative data collection is somewhat lacking, i.e. what questions were used, participants, setting, etc.

Page 10, Qualitative analysis – Please add a reference for the thematic analysis method that you used

**Results**

Overall, a description of the results based on the results presented in the table is required.

Table 1, survey 2 – There is an error on the percentage of group 51+.

Table 2, the classification ranges of ISI and IES-R are different from the description on page 8.

Please cite figures as “Fig 1”, “Fig 2” at the end of explanation, etc. as per PLOS ONE manuscript guideline.

Page 17, line 380-381 box plots 5A…. => 4A….

Figure 5 – Please consider elaborating or deleting the current (and future) financial stress as it seems to lack justification to view them as COVID-19 related psychological distress.

Page 19 – line 402-403 box plots 6A…. => 5A….

Since it seems unnecessary to duplicate tables and figures on the same subject, it is recommended to present only one.

Page 21 – In Theme 1, 2, and 5, it is difficult to guess who the subjects of the content are and whether the content is positive or negative, so it is necessary to modify the theme names so that the direction is presented. In addition, please consider to provide sub-categories therefore it is possible to understand how these themes were derived from the qualitative data.

On page 21, theme #6 (theme #4 on page 27) is missing, and it does not match the theme presented on page 27-29.

Theme 1 – It is little difficult to understand due to lack of explanation in the text on how communication and planning are grouped into one topic.

Page 22 Line 460-462 - It is difficult to see this as a problem due to the lack of advice related to COVID-19.

Page25 line 529- It seems inappropriate to mention the patient's vulnerability and the nurse's vulnerability within the same theme,

Line 533-537, this statement does not appear to be a difficulty with the participant's (nurse) work or COVID-19 related Psychological Distress.

Research questions about qualitative analysis are not described in the research purpose and methods. In the 5-6 derived themes, in particular, planning and accessibility of care do not seem to be the best analysis results as COVID-19 related psychological distress.

**Discussion**

Discussions should be based on the main findings of the study. For example, line 685- on page 31 is not seen as a discussion based on the study results. Line 706-, the information related to mandatory vaccination was not presented in the result section.

Line748- It is not clear which theme is related to burnout in the qualitative analysis results.

**Conclusion** needs be described more compactly..

Line 847- Recommendations for further research – Please describe your specific research proposal in sentence format.

Since the manuscript is generally too long, it needs to be edited, and it seems appropriate to move some of the content to the discussion or delete (eg, line 808-).

It is a meaningful study as it saw the longitudinal effect of COVID-19, but it seems that the advantages of longitudinal data are not sufficiently reflected and sufficiently discussed.

Reference #10, 27, 55 - Organisation Wh => World Health Organisation
