# Supplementary material for: Stressors, manifestations and course of COVID-19 related distress among public sector nurses and midwives during the COVID-19 pandemic first year in Tasmania, Australia
Source: PLoS One. 2022 Aug 9;17(8):e0271824. doi: 10.1371/journal.pone.0271824 (PMC9362919; doi:10.1371/journal.pone.0271824)
Supplement: Attachment — Submitted filename: REBUTTAL_LETTER_PLOS_ONE_REVIEWERS_COMMENTS.docx [file pone.0271824.s005.docx]

Mr Muhammad Shahzad Aslam,	March 25, 2022
Academic Editor
PLOS ONE

Dear Sir

Re: PONE-D-21-36897

Stressors, Manifestations and Course of COVID-19 Related Distress Among Nurses and Midwives in Tasmania

Please find our responses to the Reviewers comments which address each point raised, in blue font.

Kathryn Marsden

Reviewers' comments:

Reviewer's Responses to Questions

**Comments to the Author**

1. Is the manuscript technically sound, and do the data support the conclusions?

The manuscript must describe a technically sound piece of scientific research with data that supports the conclusions. Experiments must have been conducted rigorously, with appropriate controls, replication, and sample sizes. The conclusions must be drawn appropriately based on the data presented.

Reviewer #1: Yes

Reviewer #2: Partly

2. Has the statistical analysis been performed appropriately and rigorously?

Reviewer #1: Yes

Reviewer #2: No

3. Have the authors made all data underlying the findings in their manuscript fully available?

The [PLOS Data policy](https://protect-au.mimecast.com/s/ooR_CL7rNltRXlVoQuAVlC8?domain=aus01.safelinks.protection.outlook.com) requires authors to make all data underlying the findings described in their manuscript fully available without restriction, with rare exception (please refer to the Data Availability Statement in the manuscript PDF file). The data should be provided as part of the manuscript or its supporting information, or deposited to a public repository. For example, in addition to summary statistics, the data points behind means, medians and variance measures should be available. If there are restrictions on publicly sharing data—e.g. participant privacy or use of data from a third party—those must be specified.

Reviewer #1: Yes

Reviewer #2: No

**Author**: All data has been uploaded and called ‘Supplementary File.’

4. Is the manuscript presented in an intelligible fashion and written in standard English?

PLOS ONE does not copyedit accepted manuscripts, so the language in submitted articles must be clear, correct, and unambiguous. Any typographical or grammatical errors should be corrected at revision, so please note any specific errors here.

Reviewer #1: Yes

Reviewer #2: Yes

5. Review Comments to the Author

Please use the space provided to explain your answers to the questions above. You may also include additional comments for the author, including concerns about dual publication, research ethics, or publication ethics. (Please upload your review as an attachment if it exceeds 20,000 characters)

Reviewer #1: PONE-D-21-36897
Thank you for your contribution to the PLOS ONE.
This study describes the critical stressors and how they manifest within both the work and larger social environment for nurses and midwives in Australia.
I have following comments to the authors

Abstract
1. There seems to be a lack of definition in the text of organizational burnout. The Manuscript contains few statements on nurses’ burnout. As burnout was not directly measured in this study, it is necessary to provide an additional explanation about the relationship between burnout and psychological distress investigated in this study

**Author**: Additional information has been included to explain the relationship between burnout and psychological distress.

2. Page 3, the full terminology of PTSD is required
**Author**: Corrected

*Method*

3. Page 6, participants – It needs additional information on how many people were surveyed and what the response rate was

**Author:** A respondent flow diagram has been included illustrating these details

4. Page 8, line 196-7 & page 9, line 205-207 – They seem unnecessary as measurement descriptions **Author:** These have been deleted as suggested.

5. The description of qualitative data collection is somewhat lacking, i.e. what questions were used, participants, setting, etc.

**Author:** The qualitative data section has been expanded to include study design, survey timepoints, methods, participants and test battery information

6. Page 10, Qualitative analysis – Please add a reference for the thematic analysis method that you used

**Author:** Reference added

7. Results
Overall, a description of the results based on the results presented in the table is required.

**Author:** Description added

8. Table 1, survey 2 – There is an error on the percentage of group 51+.

**Author:** This error has been corrected

9. Table 2, the classification ranges of ISI and IES-R are different from the description on page 8.
Please cite figures as “Fig 1”, “Fig 2” at the end of explanation, etc. as per PLOS ONE manuscript guideline.

**Author:** This error has been corrected

10. Page 17, line 380-381 box plots 5A…. => 4A

**Author:** This has been corrected and replaced

11. Figure 5 – Please consider elaborating or deleting the current (and future) financial stress as it seems to lack justification to view them as COVID-19 related psychological distress.

**Author:** The relationship between COVID-19 related psychological distress and the domains of current and future financial stress have been referenced and justified. The purpose of inclusion of the various different predictors of psychological stress is to determine which potential stressors appear to be of greater or lesser importance to the psychological state of nurses and midwives during the first year of the Covid-19 pandemic. The error that the reviewer has identified is our use of the term “COVID-19 related psychological distress” in the title of the manuscript, since Tasmanian nurses appear to have only one psychological persona and not multiple independent personas. The paper is actually about the relative importance of different predictors of psychological distress in the nurses during this time, some of which they may judge to be altered by the pandemic, and some that they might not so judge. There are no specific or a defined list of Covid-specific stressors. The title of the manuscript has been changed, ‘Stressors, Manifestations and Course of Psychological Distress Among Nurses and Midwives during the Covid-19 pandemic first year in Tasmania, Australia.

12. Page 19 – line 402-403 box plots 6A…. => 5A….
Since it seems unnecessary to duplicate tables and figures on the same subject, it is recommended to present only one.

**Author:** All duplicate figures and information have been deleted.

13. Page 21 – In Theme 1, 2, and 5, it is difficult to guess who the subjects of the content are and whether the content is positive or negative, so it is necessary to modify the theme names so that the direction is presented. In addition, please consider to provide sub-categories therefore it is possible to understand how these themes were derived from the qualitative data.

**Author:** Response trajectories have been formed and categorised as ‘positive’, ‘negative’, ‘mixed’, or ‘neutral’. Themes and additional subthemes have been added.

14. On page 21, theme #6 (theme #4 on page 27) is missing, and it does not match the theme presented on page 27-29.

**Author:** Theme names have been corrected and consistent.

15. Theme 1 – It is little difficult to understand due to lack of explanation in the text on how communication and planning are grouped into one topic.

**Author:** Theme redefined with the addition of a subtheme for clarity

16. Page 22 Line 460-462 - It is difficult to see this as a problem due to the lack of advice related to COVID-19.

**Author:** Statement reviewed and recategorized

17. Page25 line 529- It seems inappropriate to mention the patient's vulnerability and the nurse's vulnerability within the same theme.

**Author:** This has been done intentionally to illustrate the permeable boundaries. Theme renamed with the addition of a subtheme to increase clarity

18. Line 533-537, this statement does not appear to be a difficulty with the participant's (nurse) work or COVID-19 related Psychological Distress.

**Author:** This statement exemplifies blurring of the nurse and midwife’s professional and homelife roles and is catalogued under new theme of ‘Occupational risk’ with subtheme ‘blurred role’

19. Research questions about qualitative analysis are not described in the research purpose and methods.

**Author**: Qualitative questions added

20. In the 5-6 derived themes, in particular, planning and accessibility of care do not seem to be the best analysis results as COVID-19 related psychological distress.

**Author:** Theme reorganised. Poor planning now falls under the Theme: Miscommunication and subtheme: inadequate leadership

21. Discussion
Discussions should be based on the main findings of the study. For example, line 685- on page 31 is not seen as a discussion based on the study results

**Author:** Discussion reviewed and corrected

22. Line 706-, the information related to mandatory vaccination was not presented in the result section**.**

**Author:** Statement deleted

23. Line748- It is not clear which theme is related to burnout in the qualitative analysis results**.**

**Author:** Themes reorganised and corrected

24. Conclusion needs be described more compactly.

**Author:** Corrected

25. Line 847- Recommendations for further research – Please describe your specific research proposal in sentence format.

**Author:** corrected

26. Since the manuscript is generally too long, it needs to be edited, and it seems appropriate to move some of the content to the discussion or delete (eg, line 808-).

**Author:** Corrected

27. It is a meaningful study as it saw the longitudinal effect of COVID-19, but it seems that the advantages of longitudinal data are not sufficiently reflected and sufficiently discussed.

**Author:** Longitudinal data added to results and discussed more fully

28. Reference #10, 27, 55 - Organisation Wh => World Health Organisation

Author: corrected

***Reviewer #2:*** *1. The method used to treat missing data in this study is mean substitution, but it tends to reduce variability, increase R^2, and decrease SE by design. According to Dr. Paul Allison at the University of Michigan, “All of these [single] imputation methods suffer from a fundamental problem: Analyzing imputed data as though it were complete data produces standard errors that are underestimated and test statistics that are overestimated. Conventional analytic techniques simply do not adjust for the fact that the imputation process involves uncertainty about the missing values.” It is strongly recommended to use other advanced methods, such as multiple imputation (MI) or full information maximum likelihood (FIML), to handle missing data. FYI, in many situations, these methods can also handle missing data in outcomes as well. Or even listwise deletion might be a better option than mean substitution.*

**Author:** Agreed. The analyses have been repeated using the multiple imputation methodology of Stata. An expanded description of this and other methodological issues were included in an added Supplementary Materials document.

*2. Please provide the missing data information (N valid and invalid for each variable) for all variables used in this study.*

Author: All N data provided for variables

*3. How the outcomes (PHQ-9, GAD-7, ISI, IESR-22) were treated for analysis is unclear. The authors stated that “The four primary outcome scales derived from the battery of psychological tests (PHQ9, GAD7, ISI, IESR 22) are rank ordered with relatively broad ranges but are USUALLY TREATED as continuous interval variables for purposes of statistical analysis.” It seems like they were treated as continuous (Table 3) AND ordinal (Tables 4 and 5), but it is unclear what it means by “usually treated as continuous interval variables”.*

**Author:** The words used in the manuscript were an abbreviated description, with additional unhelpful commentary by us. We agree with the reviewer’s comments and have endeavoured to provide a more coherent analysis and presentation.

We have decided to provide and expanded description in Additional Materials, to accommodate the request in Comment 6 for further information. This has allowed us to write a comprehensive explanation and discussion of the use of the statistical methodologies to achieve the more precise objectives of the statistical analysis. It will also allow us to provide the information requested in this review, with an explanation of what (and why) has been selected for presentation in the main text.

The analysis has been performed using multiple imputation repeated-measures ordered logistic regression, with summary measures presented as medians and interquartile ranges. Testing the assumptions of linear regression demonstrated high degrees of heteroskedasticity, skewness and kurtosis of the regression residuals (see Additional Materials) violating those assumptions, which suggested that it would be unreliable to present the regression analysis as the linear regression methods suggested for this particular set of data. We recognize that it is preferable to use “parametric” methods where possible, but in this case we were not happy to do so.

Stata 16 MP2 (the statistic package available to us for this analysis) does not support multiple imputation versions of multilevel mixed effects ordered logistic regression, which we would have preferred to have used as the reviewer suggested.

*4. How the longitudinal data was analyzed is confusing and unclear. There are three-time points, and the outcomes can be treated as continuous or ordinal. There are four outcomes, hence there should be 8 models (=4 outcomes continuous and ordinal) at least. Then a linear mixed model can be used with a continuous/ordinal outcome and time as a fixed effect controlling for the covariates. In each model, the “trend” can be shown by testing the interaction between time and a predictor. Then marginal predictions can be calculated by using the program based on the model estimates along with estimate/SE/95% CI, not observed values (mean/SD). Figures need to be based on these values. Also, it seems that the data from three times were merged (Tables 4 and 5) for some reason then analyzed, while they could have been handled within a linear mixed model regardless of the nature of the outcome (continuous or ordinal).*

**Author:** The analysis has been presented as suggested. The odds ratios for the association between the individual predictors were estimated for each of the surveys, and as was the change in those associations over time (as the reviewer suggested, using a time interaction term).

*5. Please consider treating the outcomes as either continuous or ordinal, not both. It is a matter of how each outcome needs to be handled to better answer the research questions of the study. It is a technical as well as theoretical question that need to be answered before running any statistical model.*
**Author:** This has been clarified in the Additional Materials document. The summary measures will be presented as medians (IQR) rather than means (SD). The presentation has been made purely rank-ordered (“non-parametric”) in nature.

6. Please provide more details about each linear mixed model, such as model fit (e.g., AIC/BIC), sample size, the statistical method for fitting the model (e.g., ML or REML), the structure of the covariance matrix for the random effects, and the structure of the residual errors within the lowest-level groups. None of these are reported.

The organizing idea behind the analysis of the data is that:

1. The intention was to collect some information that might assist the managers of the health service to lessen the psychological distress that was expected to be produced by the emerging pandemic. The planning of this study occurred necessarily rapidly due to the fast-moving nature of the pandemic. The questionnaire design was undertaken and administered first before access to (somewhat) skilled statistical assistance was available.
2. An anonymous online questionnaire has no possible chance of describing the full range of causes of variation in levels of psychological distress occurring amongst the nursing and other staff affected by the Covid-19 pandemic.
3. What might be possible is to identify the relative strength of those factors we had identified as being able to be assessed at some sort of level. There may have been other factors that could have been assessed that did not occur to us, but that could have been assessed in such a questionnaire. There will be other factors that require in-depth individual evaluation by trained counsellors, which would have important (probably more important) influences on the distress.
4. The level of confounding that can be expected to be present will be quite high. The absence of important causal factors in the study data will render any precise measurement of association somewhat redundant due to the confounding that can be expected: i.e. any absolute numbers describing any association can be expected to be inaccurate.
5. The factors chosen to be included in the questionnaire those that were thought to be relevant to a personnel management response. Many of these factors had no readily available validated measurement tools.
6. Thus, we had adequate measurement tools for the outcome measures of psychological distress, but crude tools for measurement of the predictors of that distress, and the range of those predictors was truncated by managerial considerations.
7. There is no reason to suppose that the predictor measurement tools will be understood and responded to by the respondents to the questionnaire in a consistent manner.
   a. The best that can be hoped is that individual respondent would interpret the predictor questions in a similar way for each of the different predictors. Thus, it is our judgement that the most reliable analysis that can be performed using the study methodology and the resultant data is a comparison of the relative strength of association between the outcomes (4 outcomes analysed separately) and predictors.
   b. The regression coefficients for individual predictor questions need to be estimating zeroed on the “no effect” responses, but without any adjustment for any differences in variability.
8. Experience of the analysis demonstrated substantial violations of the assumptions of linear regression. Therefore it was judged to be more reliable to perform the analysis using rank-ordered statistical tests: i.e. repeated-measures multivariate ordered logistic regression was chosen. Using Stata, we had the choice of multilevel mixed-effects ordered logistic regression (meologit), or repeated-measures multivariate ordered logistic regression with multiple imputation (mi estimate: ologit); Stata 16.1 does not support mi estimate: meologit. Our final choice was mi estimate: ologit, although we will present both as a sensitivity analysis in the Supplementary materials

Clearly, this was not adequately presented in the original manuscript, and we hope that, with your advice, we will improve the clarity or our presentation. The technical details of the analysis are not appropriate for the primary manuscript, since few readers will have the understanding or interest in such technicalities, so will be relegated to the Supplementary Materials section. Imposition of such details will simply impair the ability of the readers who have the responsibility of translating the presentation of this type of study into useful health service practice.

The reviewer may be unfamiliar with the way that Stata works. When using multiple imputation, the post estimation syntax does not allow the sort of model fit analysis Reviewer 2 suggests. The reviewer does not provide any justification for the requested numbers, and they may not be relevant for the fixed effects (only) rank-ordered analysis which has been selected.

Nevertheless, what is being suggested here is addressing the question of whether the estimates of the association would be altered by including more or fewer variables in the regression models. This question was tested by presenting the results of a full multiple imputation repeated-measures ordered logistic regression model for each of the four outcomes (including all the relevant predictor variables available) and a more limited model (with the removal of predictor variables with below-threshold significance values). This has been presented in the Additional Materials. This shows that the inclusion or exclusion of the low-significance variables resulted in only trivial differences in the odds ratios and 95% confidence intervals of those: i.e. the numbers of variables included were not changing the estimates of the ORs of the variables that appeared to be having the greatest effects.

As I understand it, the primary purpose of AIC/BIC estimates is to help choose the model to be fitted with the fewest effective numbers of predictors for inclusion in the final model. This makes sense when the optimum absolute prediction of the outcomes from predictors is the objective of the analysis. As indicated in the discussion of Comment 7 above, that is not the objective of the analysis. We are interested in the relative effect of all of the measured predictors, in order to guide behaviour in the messy real world. What seems to be being recommended involves selective reporting of risks by assigning inaccurately zero effect to a proportion of the predictor variables: we cannot know whether there are in fact zero effects of those variables; that is simply an assumption of the procedural methodology. We understand the dangers of over-specification inherent in this approach, but the combination of relatively large numbers of respondents, and performance of sensitivity analysis should minimize such risk. Nevertheless we will try and accommodate this request.

*7. Study hypotheses are implied but not clearly stated at all in the introduction.*

**Author:** Corrected and now clearly stated

8. On line 21, please provide the details about “3 timepoints over 12-month”: baseline / 3-month / 12-month.

**Author:** Additional information and details around the 3 timepoints is addressed in Study Timelines section and has been clarified by dates.

9. The terms used to describe the statistical model on line 26-27 and 215-216, “mixed effects linear regression and linear mixed model analyses” and “mixed effects linear regression and mixed effects ordered logistic regression”, are very confusing and somewhat incorrect. The statistical method used for analysis is a linear mixed model (aka multilevel model) with continuous and nominal outcomes. Or it can be specified as “multivariate mixed effects ordered logistic regression” as the authors specified on line 223-224.

**Author:** Corrected and clarified. Due to previous comments, we have re-analysed using repeated measures ordered logistic regression with missing data substituted by multiple imputation.

10. On line 144-146, please discuss how the exclusion of the North West region hit hard by the pandemic would have affected the data and the results. They seem to be the one that might have been most impacted by the pandemic but missing from the data.

**Author:** Issue discussed in the discussion and conclusion sections

11. Please add more details about how demographic and “other relevant” (please specify) predictor variables were measured (line 149-157). For example, it is unclear how “education level” or “community exposure” was measured by the survey. They are somewhat described in tables, but the authors should provide much more details about the variables used for analysis with text.

**Author:** Additional details provided around the predictor variables in Additional Materials Table A3.

12. Please specify what measure (e.g., Cronbach’s alpha) was used for “internal consistency”.

Author: There were seven statements of “internal consistency”, each quoting an alpha value, which referred to Cronbach’s alpha. This is now specified in the Additional Materials

13. Line 210-211 is not necessary. Please consider removing the sentence.

**Author:** agreed line removed

14. Please add the total N for each variable in Table 1.

**Author:** Table 1 has been revised, with this issue corrected.

15. In Tables 4 and 5, the outcome variables are listed as predictors. Please reorganize the tables.

**Author:** Corrected

6. PLOS authors have the option to publish the peer review history of their article ([what does this mean?](https://protect-au.mimecast.com/s/fMheCMwv0mfq9vDozu9bJnt?domain=aus01.safelinks.protection.outlook.com)). If published, this will include your full peer review and any attached files.


If you choose “no”, your identity will remain anonymous but your review may still be made public.


**Do you want your identity to be public for this peer review?** For information about this choice, including consent withdrawal, please see our [Privacy Policy](https://protect-au.mimecast.com/s/D7iRCNLwPnf09wJnjC7tLSx?domain=aus01.safelinks.protection.outlook.com).

Reviewer #1: No

Reviewer #2: No
