# Supplementary material for: Stressors, manifestations and course of COVID-19 related distress among public sector nurses and midwives during the COVID-19 pandemic first year in Tasmania, Australia
Source: PLoS One. 2022 Aug 9;17(8):e0271824. doi: 10.1371/journal.pone.0271824 (PMC9362919; doi:10.1371/journal.pone.0271824)
Supplement: Attachment — Submitted filename: PONE-D-21-36897-REBUTTAL_LETTER_REVIEWERS_COMMENTS.docx [file pone.0271824.s006.docx]

Additional Editor Comments:

Please correct the mistakes given by reviewer and resubmit for further review

[Note: HTML markup is below. Please do not edit.]

Reviewers' comments:

Reviewer's Responses to Questions

**Comments to the Author**

1. If the authors have adequately addressed your comments raised in a previous round of review and you feel that this manuscript is now acceptable for publication, you may indicate that here to bypass the “Comments to the Author” section, enter your conflict of interest statement in the “Confidential to Editor” section, and submit your "Accept" recommendation.

Reviewer #1: All comments have been addressed

Reviewer #2: (No Response)

2. Is the manuscript technically sound, and do the data support the conclusions?

The manuscript must describe a technically sound piece of scientific research with data that supports the conclusions. Experiments must have been conducted rigorously, with appropriate controls, replication, and sample sizes. The conclusions must be drawn appropriately based on the data presented.

Reviewer #1: Yes

Reviewer #2: Yes

3. Has the statistical analysis been performed appropriately and rigorously?

Reviewer #1: Yes

Reviewer #2: Yes

4. Have the authors made all data underlying the findings in their manuscript fully available?

The [PLOS Data policy](https://protect-au.mimecast.com/s/fsRMCnxyDVH7gz5qjUK4PsE?domain=aus01.safelinks.protection.outlook.com) requires authors to make all data underlying the findings described in their manuscript fully available without restriction, with rare exception (please refer to the Data Availability Statement in the manuscript PDF file). The data should be provided as part of the manuscript or its supporting information, or deposited to a public repository. For example, in addition to summary statistics, the data points behind means, medians and variance measures should be available. If there are restrictions on publicly sharing data—e.g. participant privacy or use of data from a third party—those must be specified.

Reviewer #1: Yes

Reviewer #2: Yes

5. Is the manuscript presented in an intelligible fashion and written in standard English?

PLOS ONE does not copyedit accepted manuscripts, so the language in submitted articles must be clear, correct, and unambiguous. Any typographical or grammatical errors should be corrected at revision, so please note any specific errors here.

Reviewer #1: Yes

Reviewer #2: (No Response)

6. Review Comments to the Author

Please use the space provided to explain your answers to the questions above. You may also include additional comments for the author, including concerns about dual publication, research ethics, or publication ethics. (Please upload your review as an attachment if it exceeds 20,000 characters)

Reviewer #1: Since this manuscript is rather lengthy for the reader to read, it is recommended to make the manuscript more compactly.

*Reviewer #2: I really appreciate time and effort to address the comments and suggestions with details. The manuscript has a lot of valuable and precious information for a variety of readers in the context of pandemic. A few additional comments and suggestions.

I agree that not many will be interested or have understanding in the technicalities of the statistical analysis used for this manuscript. However, I believe it is important to report technical details as much as possible since it is a peer-reviewed academic manuscript. And the manuscript is already very technical with many numbers to interpret. I see that a lot of information is provided as supplement, but it would be very informative if some key information at least can be added in the main text. For example, key details about multiple imputations, such as number of imputations (and justification) and auxiliary variables used if used any. How some assumptions were violated, what command was used in Stata, and/or what was not available in the Stata version you used, are all important but not essential, hence they can be placed in the supplement or briefly mentioned in text in my opinion.*

AUTHOR COMMENT: In the original analysis, 20 imputations were made, using all the outcome and predictor variables measured as determinants of the imputation (except for the answer to Question 28, which produced a majority of responses as “not applicable”, suggesting that respondents had not idea what to make of the question, or consider it irrelevant). The Multivariate Chained Monte Carlo method was used for the imputations, as implemented by Stata. This information has been included in the main manuscript.

As to the justification of the use of 20 imputations, my previous awareness of the state of understanding of multiple imputation methodology was that a small number (4 or 5) was considered adequate, but that I used large numbers just in case. Having examined the more recent statistical literature in light of the Reviewer 2 comment requesting justification, I was able to identify methodology that would be appropriate to supply an answer. Unfortunately, the methodology required complicated analysis and judgements about each of the outcome and predictor variables that I did not feel confident about making: the calculations require judgements about minimum absolute effect sizes that would require further empirical data collection. As a result, the main analyses were repeated using 200 imputations, a pragmatic guess about the balance between analytic time and analytic precision. The concern about numbers of imputations appear to revolve around lack of precision when too few imputations are performed. Inspection of the numbers produced by 200 versus 20 imputations revealed only trivial differences, and that these differences were small compared to the 95% confidence intervals.

*Removing or including variables in the model is more of a matter of theoretical considerations, not significance, in my opinion. The author(s) reasonably explained the decision about variable choices. It is impossible for observational data to measure everything for sure, and the author(s) appropriately went through various technical and theoretical considerations in depth.*

AUTHOR COMMENT: There are a large number of practical and theoretical considerations surrounding the conduct of study data analyses. There will be a large variation in the emphasis that different professionals will place on the different considerations involved. It is incumbent on a reviewer to be clear about their choice of what is important, what is the meaning of their queries and objections, and what they think may be a more appropriate response with appropriate justification for those suggestions. Reviewer 2 has been consistently opaque in their comments, failing to explain or justify their comments. Hence, we have been forced to make an extensive response that we think might be covering what was meant, without knowing for certain that we have been interpreting the comments as they were intended.

*In general, -2LL, AIC, and BIC are the trios of model fit when it comes to longitudinal data analysis, and they do not always agree. FYI, AIC weight number of parameters more compared to BIC while BIC takes N into the calculation as well as number of parameters. My suggestion is reporting the range of model fits based on the analysis results with the multiple imputation datasets. Perhaps, the model fits without using MI might be preferred to range. Yes, no command is available yet when multiple imputation command (mi) was used in Stata, but it does not mean that no model fit needs to be reported.*

AUTHOR COMMENT: We have attempted to comply with the spirit of this request. The suggestion of how this might be accomplished is not viable, since running the regression analyses without substitution of missing data results in about 50% loss of respondent contribution to the case numbers: a loss of a single value in a multiple regression models causes that case to drop from the analysis being conducted. Instead, the mean of the imputations for each outcome and predictor variable were calculated, and that value was used when the data point was missing. The regression analyses were then run on this pseudo-imputed version of the data. This will not be a true reflection of the model fit statistics, but it seems the best we can do to satisfy your request. These were then appended to the tables in the main manuscript.

We make no interpretation of these model fit statistics.

*Please explain why time was treated as numeric (.1, .29, and 1.1 years) instead of categorical. There are only three time points. How time was treated in the current models assume a linear trend over time.*

AUTHOR COMMENT: There appears to have been a misunderstanding about the conduct of this study, probably because of the use by us of the inaccurate term “Timepoint”. This term implies that all surveys were completed on the same day. Each survey was open nominally for 21 days, although in practice the respondents were able to access the survey for longer periods:

Survey 1: Responses between Day 4 (5/04/2020) and Day 62 (2/06/2020) (95% by Day 48)

Survey 2: Responses between Day 95 (5/07/2020) and Day 222 (9/11/2020) (95% by Day 130)

Survey 3: Responses between Day 390 (26/04/2021) and Day 449 (24/06/2021) (95% by Day 440)

Thus, “timepoint” is an inaccurate description of the time variable, hence the use of the time as a numerical variable. The time of completion of the survey was the only completely reliable form of data (time stamp was generated by the online software), and it was felt that there was useful data available here.

We also would disagree with the conversion of the time of survey completion from an interval data value to a categorical data value. Actually, categorical in this context is an imprecise shorthand for the appropriate mathematical concept, since what is being done by this conversion is to turn the interval data into nominal data. (Data is classified mathematically as ratio, interval, rank-ordered or nominal, each with its own recognised properties. Categorical data is not normally a term used in this classification system, since it can imply either a nominal or rank-ordered entity, depending on the context of use, and whether the categorical data is outcome or predictor). This is important because of the way nominal predictor data is handled by regression software. With interval data, the numbers 1, 2, 3 are treated as an exact sequence with exact order and value differences. With nominal data, the numbers 1, 2, 3 are treated as names with no implied order or value differences. If you change the labelling of nominal data so that 1 becomes 3, 3 becomes 2 and 2 becomes 1, it makes no difference to the resulting regression coefficients (i.e. the means and mean differences estimated for each time period). It is not correct to conclude that the differences between the outcome estimates across time from nominal time-predictors accurate reflect what is actually happening.

Further, since the time intervals between the three survey periods were different, (Survey 1 to Survey 2 was about 3 months, whilst Survey 2 to Survey 3 was about 9 months), conversion of interval data to nominal data will lose this time difference. This will make interpretation of any differences found less clear.

Presumably, the thinking behind this suggestion is that the change with time is not linear. There is likely to be some sort of time relationship, but it would be more appropriate to explore the use a non-linear form of association within the regression model, than to completely abandon the attempt to identify that relationship. However, given the uncertainties surrounding the accuracy of most of the survey measurement tools (especially for the predictor variables), and the relatively wide 95% confidence intervals of the estimates being made, it would seem unlikely that a definitive non-linear relationship could be found for each of the predictor variables (there is no reason to believe that the optimum non-linear relationships for each of the predictors would be similar). Therefore, it seems to us that the linear relationship, applied to all of the predictors, is a reasonable choice in these circumstances. The means at each mean survey completion time are shown in the tables, and each reader can then make their own judgement about the optimum relationship format.

*Please explain how the online data collection was done with details. It is unclear if the study used a online data collection platform, such as REDCap, Qualtrics, QuestionPro, or else, or a document was attached to the email sent and participants sent the completed surveys back to the researchers.*

AUTHOR COMMENT: The online survey was conducted using Survey Monkey. Alteration to manuscript made.

*Please consider merging “Survey timepoints” with “Study design”.*

AUTHOR COMMENT: Agreed, and merged

*Please be consistent with the terms for the surveys. It is unclear if “survey one or survey 1”, “survey two or survey 2”, and “survey three or survey 3” mean “survey” at baseline, 3-month, and 12-month, respectively.*

AUTHOR COMMENT: Agreed, and corrected

*The author(s) explained that Survey one was only distributed to Southern Tasmanian region, and Survey two was distributed only to the Southern and Northern regions, while Survey three was distributed to Southern, Northern, and North-western regions. It seems like only those who were in the Southern region could complete the surveys at all three time points, which means that 1) the participants from the other two regions have missing data for the survey at baseline (Survey one), and that 2) the participants from the North-western region have missing data for the surveys at baseline and 3-month. It would be very information if the author(s) can share some insight about how this might have impacted the representativeness of the data compared to the general population of nurses and midwives in Australia.*

AUTHOR COMMENT: It was intended that the survey respondents be representative of nurses in Tasmania. Covid-19 did not spread in a linear or predictable path across Tasmania, instead we saw sporadic flux; periods of outbreaks.

The prohibition of survey distribution to the nurses and midwives at Survey 1 and 2 has potentially caused underrepresentation of this group of nurses and midwives at these study periods, however authors deemed it important to be as inclusive as possible enabling those who wanted to share their experience to do so at Survey 3, once restrictions had been eased. Although the impact of being unable to offer the survey to the northern regions is unknown, it mirrors the course of the COVID response across Tasmania. Authors have included discussion surrounding this in the ‘Discussion’ section.

The reviewer makes a point about “missing data”. We are not sure what the reviewer means by this. If anything, this would be a failure to recruit through the survey period (one year), or a changing sample-frame. Missing data implies data not collected from subjects who have been recruited, and would be subject to substitution using multiple imputation to correct this lack of complete data.

Is there an implication that the respondents who completed the later surveys should be subject to a multiple imputation process to substitute the data for the earlier survey that they did not complete? As reported in the manuscript, one form of data that cannot be substitute is the coded identification of the respondents that would allow linkage of more than one survey if they responded more than once. To impute a connection between unidentified surveys would be a completely inappropriate procedure, as would imputing data for surveys that have not been completed at all. All multiple imputation procedures require some minimum amount of data, otherwise the cases without data are completely (and properly ignored).

*In Figure 1, please add the month of the first survey (April).*

AUTHOR COMMENT: Corrected

7. PLOS authors have the option to publish the peer review history of their article ([what does this mean?](https://protect-au.mimecast.com/s/J-lYCoVzE9Hr5M4LZiNIaTR?domain=aus01.safelinks.protection.outlook.com)). If published, this will include your full peer review and any attached files.


If you choose “no”, your identity will remain anonymous but your review may still be made public.


**Do you want your identity to be public for this peer review?** For information about this choice, including consent withdrawal, please see our [Privacy Policy](https://protect-au.mimecast.com/s/wASSCp8AG6fnqGDNKCBerqn?domain=aus01.safelinks.protection.outlook.com).

Reviewer #1: No

Reviewer #2: No
