# Supplementary material for: Stressors, manifestations and course of COVID-19 related distress among public sector nurses and midwives during the COVID-19 pandemic first year in Tasmania, Australia
Source: PLoS One. 2022 Aug 9;17(8):e0271824. doi: 10.1371/journal.pone.0271824 (PMC9362919; doi:10.1371/journal.pone.0271824)
Supplement: Attachment — Submitted filename: PONE-D-21-36897_RESUBMISSION3_REBUTTAL_Reviewers_Comments.docx [file pone.0271824.s007.docx]

**From:** [em.pone.0.7c20bb.fffdef87@editorialmanager.com](mailto:em.pone.0.7c20bb.fffdef87@editorialmanager.com) <[em.pone.0.7c20bb.fffdef87@editorialmanager.com](mailto:em.pone.0.7c20bb.fffdef87@editorialmanager.com)> on behalf of PLOS ONE <[em@editorialmanager.com](mailto:em@editorialmanager.com)>
**Sent:** Tuesday, June 21, 2022 4:13:27 AM
**To:** Marsden, Kathryn M (Clinical Educator) <[kathryn.marsden@ths.tas.gov.au](mailto:kathryn.marsden@ths.tas.gov.au)>
**Subject:** PLOS ONE Decision: Revision required [PONE-D-21-36897R2] - [EMID:ab690cc1d4420cc0]

PONE-D-21-36897R2

Stressors, Manifestations and Course of COVID-19 Related Distress Among Public Sector Nurses and Midwives during the COVID-19 Pandemic First Year in Tasmania, Australia

PLOS ONE

Dear Dr. Marsden,

Thank you for submitting your manuscript to PLOS ONE. After careful consideration, we feel that it has merit but does not fully meet PLOS ONE’s publication criteria as it currently stands. Therefore, we invite you to submit a revised version of the manuscript that addresses the points raised during the review process. Please submit your revised manuscript by 20th July 2022. If you will need more time than this to complete your revisions, please reply to this message or contact the journal office at [plosone@plos.org](mailto:plosone@plos.org). When you're ready to submit your revision, log on to [https://www.editorialmanager.com/pone/](https://protect-au.mimecast.com/s/tMg5CNLwPnf01gPLOc46341?domain=editorialmanager.com/) and select the 'Submissions Needing Revision' folder to locate your manuscript file.

Please include the following items when submitting your revised manuscript:

- A rebuttal letter that responds to each point raised by the academic editor and reviewer(s). You should upload this letter as a separate file labeled 'Response to Reviewers'.
- A marked-up copy of your manuscript that highlights changes made to the original version. You should upload this as a separate file labeled 'Revised Manuscript with Track Changes'.
- An unmarked version of your revised paper without tracked changes. You should upload this as a separate file labeled 'Manuscript'.

If you would like to make changes to your financial disclosure, please include your updated statement in your cover letter. Guidelines for resubmitting your figure files are available below the reviewer comments at the end of this letter.

If applicable, we recommend that you deposit your laboratory protocols in protocols.io to enhance the reproducibility of your results. Protocols.io assigns your protocol its own identifier (DOI) so that it can be cited independently in the future. For instructions see: [https://journals.plos.org/plosone/s/submission-guidelines#loc-laboratory-protocols](https://protect-au.mimecast.com/s/fCmeCP7yRptKqLNgjIjvmmn?domain=aus01.safelinks.protection.outlook.com). Additionally, PLOS ONE offers an option for publishing peer-reviewed Lab Protocol articles, which describe protocols hosted on protocols.io. Read more information on sharing protocols at [https://plos.org/protocols?utm_medium=editorial-email&utm_source=authorletters&utm_campaign=protocols](https://protect-au.mimecast.com/s/2ApICROAWrhv830YMConnSj?domain=aus01.safelinks.protection.outlook.com).

We look forward to receiving your revised manuscript.

Kind regards,

Muhammad Shahzad Aslam, Ph.D.,M.Phil., Pharm-D
Academic Editor
PLOS ONE

Journal Requirements:

Please review your reference list to ensure that it is complete and correct. If you have cited papers that have been retracted, please include the rationale for doing so in the manuscript text, or remove these references and replace them with relevant current references. Any changes to the reference list should be mentioned in the rebuttal letter that accompanies your revised manuscript. If you need to cite a retracted article, indicate the article’s retracted status in the References list and also include a citation and full reference for the retraction notice.

[Note: HTML markup is below. Please do not edit.]

Reviewers' comments:

Reviewer's Responses to Questions

**Comments to the Author**

1. If the authors have adequately addressed your comments raised in a previous round of review and you feel that this manuscript is now acceptable for publication, you may indicate that here to bypass the “Comments to the Author” section, enter your conflict of interest statement in the “Confidential to Editor” section, and submit your "Accept" recommendation.

Reviewer #2: All comments have been addressed

Reviewer #3: (No Response)

2. Is the manuscript technically sound, and do the data support the conclusions?

The manuscript must describe a technically sound piece of scientific research with data that supports the conclusions. Experiments must have been conducted rigorously, with appropriate controls, replication, and sample sizes. The conclusions must be drawn appropriately based on the data presented.

Reviewer #2: (No Response)

Reviewer #3: Yes

3. Has the statistical analysis been performed appropriately and rigorously?

Reviewer #2: (No Response)

Reviewer #3: Yes

4. Have the authors made all data underlying the findings in their manuscript fully available?

The [PLOS Data policy](https://protect-au.mimecast.com/s/mugkCVAG1yhx1pkBWUWp5UU?domain=aus01.safelinks.protection.outlook.com) requires authors to make all data underlying the findings described in their manuscript fully available without restriction, with rare exception (please refer to the Data Availability Statement in the manuscript PDF file). The data should be provided as part of the manuscript or its supporting information, or deposited to a public repository. For example, in addition to summary statistics, the data points behind means, medians and variance measures should be available. If there are restrictions on publicly sharing data—e.g. participant privacy or use of data from a third party—those must be specified.

Reviewer #2: (No Response)

Reviewer #3: Yes

5. Is the manuscript presented in an intelligible fashion and written in standard English?

PLOS ONE does not copyedit accepted manuscripts, so the language in submitted articles must be clear, correct, and unambiguous. Any typographical or grammatical errors should be corrected at revision, so please note any specific errors here.

Reviewer #2: (No Response)

Reviewer #3: Yes

6. Review Comments to the Author

Please use the space provided to explain your answers to the questions above. You may also include additional comments for the author, including concerns about dual publication, research ethics, or publication ethics. (Please upload your review as an attachment if it exceeds 20,000 characters)

Reviewer #2: I appreciate the time and effort the author(s) have spent to address my comments. I have no further comments.

Reviewer #3:

1. Please provide numbers for participants in each wave in the section on participants. It is not clear in that section if the same people responded to each wave, or new participants were in each wave. It appears to be more of a panel series design rather than a longitudinal design, which follows the same participants across time.
2. Please state PHQ 9 as PHQ 2 is commonly used in clinical studies.
3. Provide the range of the Likert scales on line 178. In the section on qualitative data analysis, please provide the questions here.
4. How was grounded theory methodology modified? (line 201). Was a theory created?
5. Please explain what an enrolled nurse is and the nurse's educational training for this role. Line 259 is missing a noun, most likely 'data' or 'responses'.
6. Please explain what Hospital certificate and TAFE certificate means and the educational level for them.
7. Did you observe or measure the levels of pandemic prevalence during the waves, and did they correspond to the stress experienced by the nurses?

**Response to Reviewer Comments:**

Reviewer #3:

1. *Please provide numbers for participants in each wave in the section on participants. It is not clear in that section if the same people responded to each wave, or new participants were in each wave.*

There appears to be a difference in the cultural application of the general method for reporting of scientific studies between different scientific disciplines, and the discipline of Reviewer 3. This paper has been written under the auspices of the healthcare scientific community, as systematized by the CONSORT, STROBE and other related guidelines, as brought together by the EQUATOR Network. In this system, the methods sections of a paper describe the plan and intention for conduct of the study, whilst the results section describes what actually happened: i.e. the intended target for the survey is described in the methods section, and the numbers of respondents are described in the results section.

The plan for the study was to invite members of a sample frame (in this case, nurses and midwives in Tasmania, whose contact details were contained in the Tasmanian Health Services contact lists). The numbers who responded are shown in the results section, as prescribed by the EQUATOR guidelines for study reporting.

Since a large number of nurses and midwives were invited to respond to an anonymous online survey, which was to be repeated on a number of occasions, there was no way to control how many would actually cooperate and respond to the survey. As the survey was anonymous, to protect the respondents from repercussions from the heath service managers, or others the respondents may wish to protect themselves from, it was not possible (by design) to identify who had responded, and difficult to identify those who responded to the repeated surveys. The method originally chosen (asking for the respondents to create a personal code from middle name and phone number) proved to be ineffective, and it is possible that different respondents happened to choose the same code.

A possible solution might have been for the online survey corporation to include an algorithm that allocated a randomly-generated, unique alphanumeric code to each respondent, blocking further allocation of this code to any new respondent to this particular survey. The respondents would be asked whether they had responded to the survey before, asking them to enter any previously-allocated code. The respondents would be asked to record the code in their mobile phones. This process would require the cooperation of the surveyor, the online corporation, and the respondents: such cooperation could occur if suggested. However, no such algorithm was available at the time the surveys were conducted. Any statement about repeated responses by the same individual would have been inaccurate by an unknown amount. This had been acknowledged in the study limitations section.

2. *It appears to be more of a panel series design rather than a longitudinal design, which follows the same participants across time.*

The consensus amongst researchers appears to be that a panel series design is a variety of longitudinal design: e.g. Wikipedia: “A **longitudinal study** (or **longitudinal survey**, or **panel study**) is a research design that involves repeated observations of the same variables (e.g., people) over short or long periods of time (i.e., uses longitudinal data). It is often a type of observational study, although it can also be structured as longitudinal randomized experiment.^[1]^”.

A panel study design appears to require that the “panel” of potential respondents has been fully identified at the outset of the study, at least their identity. This was not possible in our study, except in the broadest sense. It might have been that the inclusion of the term “cohort” in our manuscript might have caused confusion. This term has been removed for clarity.

*3. Please state PHQ 9 as PHQ 2 is commonly used in clinical studies.*

This study used the PHQ-9 which measures depression severity. The PHQ-9 total score for 9 items ranges from 0-27. The PHQ-2 however, consists of the first 2 items of the PHQ-9 and scores range from 0-6. Therefore Authors will use the full correct name for the actual tool they employed, with the manuscript now modified accordingly.

4. *Provide the range of the Likert scales on line 178.*

There are several different response scales that correspond to the Likert description. We have now referred to the actual survey, now included in Additional Materials.

*5. How was grounded theory methodology modified? (line 201). Was a theory created?*

Using the term, ‘modified grounded theory’ was used in error and Authors have defaulted to the term, ‘thematic analysis’ to describe the process of qualitative analysis, as the development of theory was not the aim of this research. Authors employed a thematic analysis framework to systematically examine qualitative data to identify themes or patterns, using both semantic and latent levels of analysis to review participants’ responses. By employing this method the researcher can interpret and understand the information given in data collection and not just organise the responses.

6. *Please explain what an enrolled nurse is and the nurse's educational training for this role. Please explain what Hospital certificate and TAFE certificate means and the educational level for them*.

Australian nurses have a two tier regulation system as promulgated by the Australian Health Practitioners Regulatory Authority (AHPRA) and administered by the Nursing and Midwifery Board of Australia. An Enrolled Nurse completes a vocational training program (Technical And Further Education [TAFE] level certificate) and on completion is able to work under the direction of a Registered Nurse. The Registered Nurse has completed an academic program equivalent to a minimum of three years of full time study and to a Bachelor Degree.

The Scope of Practice allows for differences in the provision of patient care with the Registered Nurse accountable for the patients nursing care plan and leadership.

The academic program commenced in the 1980’s prior to which nurses were trained using an apprenticeship model in a hospital-based certificate-program system.

*7. Line 259 is missing a noun, most likely 'data' or 'responses'.*

Corrected

8. *Did you observe or measure the levels of pandemic prevalence during the waves, and did they correspond to the stress experienced by the nurses?*

A new figure (labelled Fig 2, line 264, page 13) has been added in response to this comment, describing the date of occurrence of new cases of Covid-19, and the date of the individual responses to the surveys. As can be seen, most of the survey responses were made after the initial wave of Covid-19 cases; in Tasmania, it proved to be possible, by lockdown, border closure, quarantine, and test-and-trace methods to eliminate community transmission during most of 2020 and 2021.

Substantial numbers of cases have occurred in Tasmania since Tasmania and Australia in general removed the restrictions on cross-border movement of people. This rise in infections occurred outside the time-frame of the surveys. Incidentally, it does show that elimination of virus spread was possible in this geographically-isolated and isolatable community. Widespread acceptance of Covid-19 vaccination limited the morbid impact of this later large 2022 spike in cases once that spike occurred.

As the initial Tasmanian case wave of infection occurred before most of the nurses and midwives had responded to the survey, there was no change in the temporality between the wave and their responses to the sequential surveys. They would have been aware of what was happening elsewhere in the world, gaining an understanding of what might have happened or might happen in the future. It would not be possible to distinguish actual events from the processing and understanding of the events by the nurses and midwives in the way that this comment implies.

It might have been possible that a series of surveys conducted in jurisdictions affected by recurrent waves of infection, such as in Melbourne, Australia could answer the question being asked by this Reviewer, but those who might have conducted such surveys are likely to have had more immediate work to do than to conduct such surveys. Thus this comment falls outside the scope of this survey series.
